# Overcoming traps and pitfalls leading to misinterpretation of normal EEG variants and variation of the background activity

**DOI:** 10.1007/s00415-024-12440-y

**Published:** 2024-05-18

**Authors:** Philippe Gélisse, Selim R. Benbadis, Arielle Crespel, William O. Tatum

**Affiliations:** 1 Epilepsy Unit, Hôpital Gui de Chauliac, 80 Avenue Fliche, 34295 Montpellier Cedex 05, France; 2 Research Unit (URCMA: Unité de Recherchef sur les Comportements et Mouvements Anormaux), INSERM, U661, Montpellier, France; 3 Department of Neurology, University of South Florida, Tampa, FL USA; 4 Department of Neurology, Mayo Clinic College of Medicine and Health Sciences, Jacksonville, FL USA

**Keywords:** EEG, Benign EEG variants, Epileptiform, Wicket spikes, Interictal epileptiform discharges

## Abstract

Normal EEG variants, especially the epileptiform variants, can be challenging to interpret because they often have sharp contours and may be confused with “epileptic” interictal activities. However, they can be recognized by the fact that “*most spikes or sharp wave discharges of clinical import are followed by a slow wave or a series of slow deflections*” (Maulsby, 1971). If there is no wave after the spike, electroencephalographers should be suspicious of artifacts and normal EEG variants. Most normal EEG variants display a single rhythm with the same frequency within the pattern and the morphology remains stable throughout the entire EEG recording with repetition of the same pattern. In case of doubt or difficulties with a standard EEG, it is recommended to undergo an EEG that includes sleep stages with or without sleep deprivation. Finally, epileptiform is an ambiguous term corresponding to an electroencephalographic trait. Epileptiform does not imply a pathological condition, including epilepsy. The clinical context remains the most paramount in the diagnosis of epilepsy. In this article, we propose a set of rules and guidelines to identify normal EEG variants in EEG tracings and normal variation of the background activity. It is not easy to accurately assign a specific/precise name to all EEG activity, but with an orderly approach to EEG that involves using a set of criteria, nonepileptic activity can be identified**.**

**Supplementary Information:**

The online version contains supplementary material available at 10.1007/s00415-024-12440-y.

## Introduction

Epileptiform is an ambiguous term initially proposed to describe something that resembles epilepsy or has some of its characteristics [9]. Definitions of epilepsy include at least two unprovoked seizures occurring > 24 h apart or one unprovoked seizure with a high probability of recurrence [7] that may be the presence of interictal epileptiform discharges on EEGs [34]. Epileptiform patterns refer to transients distinguishable from background activity [17]. They correspond to an electroencephalographic trait denoted by duration (spikes 20–70 ms and sharp waves 70–200 ms). The ACNS Subcommittee on Research Terminology for Continuous EEG revised the EEG terminology and aimed to avoid “epileptiform” when describing specific EEG patterns because of its clinical connotation [13, 14].

To describe a situation that involves seizures or epilepsy, the term “epileptiform” should always be used with the systematic addition of the words “interictal” or “abnormal”, i.e., interictal epileptiform discharges (IEDs) or abnormal epileptiform discharges. Indeed, some waveforms in the EEG are characterized by an “epileptiform” appearance and resemble pathological discharges [30]. Normal EEG variants represent a potential challenge serving as a pitfall for accurate interpretation of EEG for clinical purposes. Normal (benign) EEG variants can be classified based on one of three specific characteristics [16]. The “epileptiform” variants include wicket spikes (WS), small sharp spikes (SSS), 14- and 6-Hz positive bursts, and 6-Hz spike-and-slow-waves. The rhythmic variants include mu rhythm (may have a “spiky” form), rhythmic mid-temporal theta burst of drowsiness (may have a notched morphology) (RMTD), midline theta rhythm (Ciganek’s rhythm), and subclinical rhythmic electrographic discharge of adults (SREDA). Posterior variants include lambda waves and positive occipital sharp transients of sleep (POSTs). Normal variations of the background activity include fast/slow alpha variants and posterior slow waves of youth. In addition, artifacts can mimic epileptiform activity.

In the literature, a large variability in the prevalence of EEG variants can be found (Table 1). This discrepancy can first be explained by significant methodological differences, mostly due to the duration of an EEG recording with a sufficient amount of both NREM and REM sleep. Second, the identification of certain EEG variants depends upon the position and number of scalp electrodes, the condition of EEG recordings (e.g., a well-lit room for lambda waves) and the age of the population. Some EEG variants such as POSTs and 14- and 6-Hz positive bursts have an age-dependent pattern, occurring more frequently in young people and becoming less frequent with advancing age, while SREDA is most common over 50 [36]. Except for the mu rhythm, the most frequent EEG variants, such as POSTs, WS, lambda waves and 14- and 6-Hz positive bursts are reported more frequently in non-epileptic patients rather than epileptic patients [21]. Being well-acquainted with the morphology of EEG variants is thus important for ensuring adequate interpretation [3–5, 23].

**Table 1:** Prevalence of EEG variants. Literature data and comparison

	Radhakrishnan et al., 1999 [27]	Santoshkumar et al., 2009 [29]	Macorig et al., 2021 [22]
Population	1778 pat. EEG with both awake and sleep recording for at least 20 min	35,249 pat. Standard EEG which contained awake, drowsiness, and sleep tracing	1163 pat. Long-term EEG monitoring between 24 h to 8 days
Wicket spikes	0.96%	0.037%	15%
14–6-Hz positive bursts	5.68%	0.52%	8.3%
SSS	8.16%	1.85%	3.3%
RMTD	0.79%	0.122%	2.1%
6-Hz SW	2.79%	1.021%	0.1%
SREDA	0%	0.074	0%

*SSS* small sharp spikes, *RMTD* rhythmic midtemporal theta (burst) of drowsiness, *6-Hz SW* 6-Hz spike and wave bursts, *SREDA* subclinical rhythmic electrographic discharge of adults

Distinguishing EEG variants from interictal epileptiform pattern is crucial to prevent overreading and, by extension, over-diagnosing epilepsy in clinically doubtful cases [5, 19, 31] as well as to avoid an incorrect diagnosis of the epilepsy type [6]. In this article, we propose a set of rules and guidelines to help identification of EEG variants (Table 2). It is not easy to assign a specific/precise name to all unusual rhythms, but with a set of criteria, these challenging waveforms should allow identification of a transient as “nonepileptic.”

**Table 2:** The rules and recommendations for the assessment of normal EEG variants and variation of the background activity

	Rules		Recommendations
1	When the alpha rhythm is ample, all physiological and unusual waveforms are accordingly ample	→ 1	When the alpha rhythm is ample, EEGers must not be misled by the EEG pattern’s unusual amplitude
2	Absence of slow after-wave in the majority of normal variants and variation of the background activity	→ 2	If there is no wave after the spike, EEGers should be suspicious of artifacts and normal EEG variants/variations of the background activity
3	Normal EEG variants display a single non-evolving rhythm. The morphology is stable, and the same pattern repeats throughout the entire EEG recording and subsequent EEGs	→ 3	EEGers should be suspicious of EEG variants when the pattern appears monomorphic and repeats itself identically throughout the recording
4	Phase reversals merely indicate localization, not epilepsy	→ 4	Use the phrase “phase reversal” in an EEG report only to describe the localization of the pattern
5	IEDs and EEG background variations differ in their EEG reactivity patterns	→ 5	EEG patterns that react similarly to the alpha rhythm correspond to alpha-harmonic or posterior slow waves of youth
6	Drowsiness and NREM sleep facilitate the occurrence of IEDs	→ 6	When the activity decreases or stops at drowsiness or sleep onset, EEGers need to be vigilant to artifacts, variations of the background activity and Ciganek’s rhythm
7	WS are best identified during sleep	→ 7	Obtain an EEG that includes sleep stages in case of doubt and difficulties
8	REM sleep is relatively protective against IEDs and epileptic seizures	→ 8	EEGers should be suspicious of EEG variants when activity increases in REM sleep or when rhythmic discharges are observed without arousal
9	Seizures usually disrupt sleep patterns	→ 9	Consider electrode artifact, RMTD, and SREDA when the discharge occurring at drowsiness or during sleep is strictly subclinical
10	Epilepsy is and must remain a clinical diagnosis	→ 10	EEG must remain a tool used to confirm clinical hypotheses

*IEDs* interictal epileptiform discharges, *EEGers* electroencephalographers, *NREM* NonRapid eye movements, *REM* rapid eye movements

## The essential rules to know

### First rule: when the alpha rhythm is ample, all physiological and unusual waveforms are accordingly ample

The assessment of the background activity is a fundamental question when interpreting an EEG. Alpha rhythm appears in children at the age of three, mixed with posterior slow waves of youth. The alpha rhythm is ample in children and adolescents and may appear “spiky” or “apiculate” mimicking abnormal epileptiform activity, especially when it breaks down with drowsiness. The amplitude tends to decrease with age. As a first rule, when the alpha rhythm is ample, all physiological rhythms are ample in wakefulness and sleep (V waves, sleep spindles, K complexes). This fundamental rule can be applied to EEG variants. The more ample and well organized the alpha rhythm, the more ample the other physiological rhythms and, therefore, unusual rhythms.

### Second rule: absence of slow after-wave in the majority of normal variants and in normal variation of the background activity

Maulsby (1971) proposed a set of guidelines for the assessment of spikes and sharp waves in EEG tracings. As Maulsby states: “Most spike or sharp wave discharges of clinical import are followed by a slow wave or series of slow deflections. If it does not have a slow after-wave, be more suspicious of artifact or of a sudden alteration in voltage of physiological background rhythms” [24]. Most EEG variants are characterized by the absence of a slow after-wave, leading to a succession of regular waveforms with the exception of 6-Hz spike-and-slow-waves and to a lesser degree to SSS.

#### Wicket spikes

**Fig. 1:**
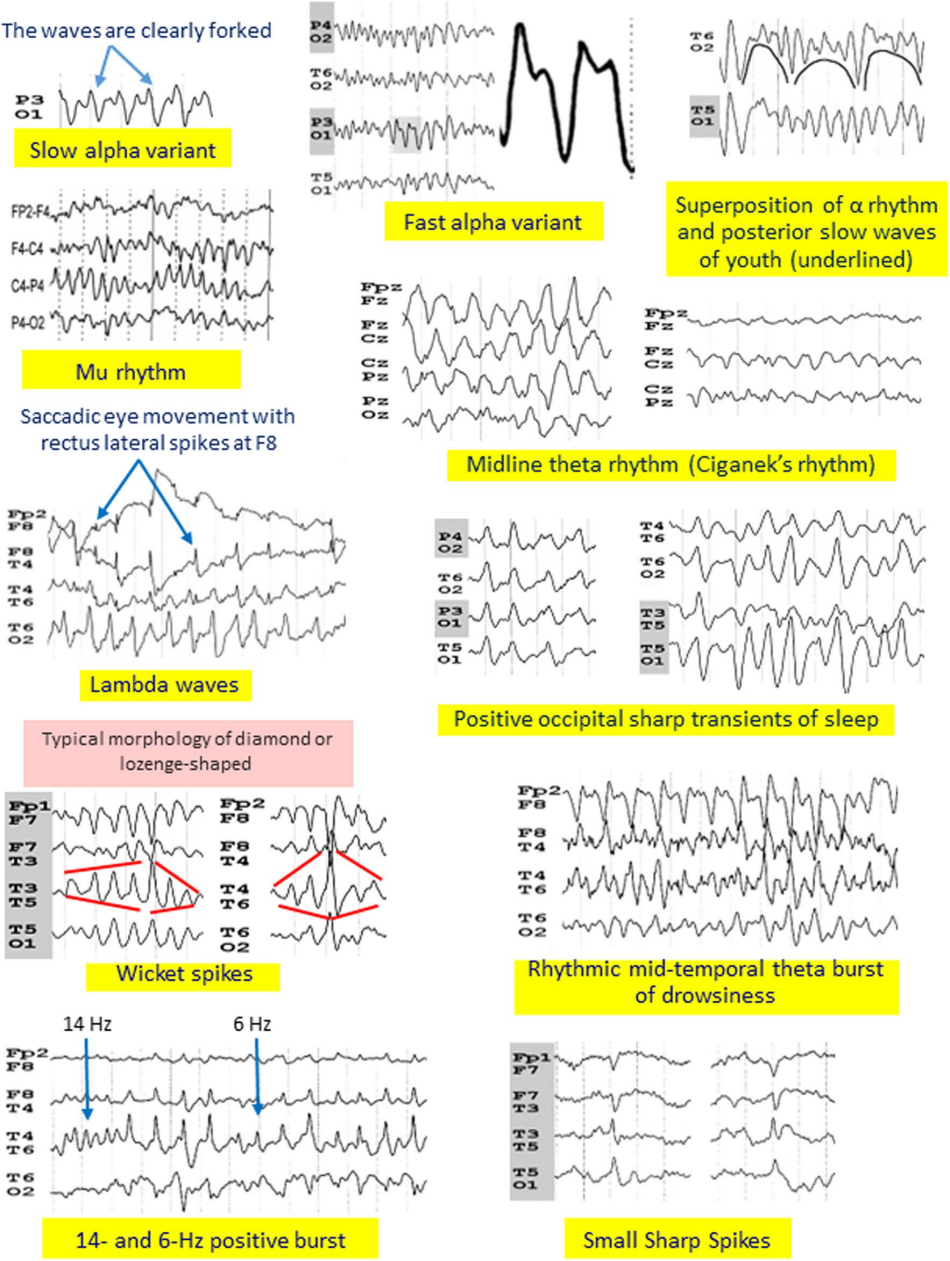
Different examples of normal variation of the background activity and normal EEG variants

WS are the best example of a burst of sharply contoured waves without slow after-wave to identify them as a normal variant*.* The prevalence of WS was initially reported as around 1%, but considerably higher in studies that used long-term EEG recordings, ranging between 10 and 15% [22] (Table 1), making WS probably the mostly misinterpreted benign variants [3–5]. From 3050 consecutive EEG recordings from 2319 patients, sharp transients, including WS, were observed at 19% [37]. WS correspond to monophasic arciform waves that occur over the temporal regions, either bilaterally or unilaterally. They appear most often in bursts with a typical morphology of diamond or lozenge-shaped (Fig. 1; Supplementary), but occasionally singly, in which case they resemble an epileptiform activity. In these situations, it is necessary to compare them with longer runs in the same tracing. The morphology of this sharply contoured wave is identical to the morphology of the most sharply contoured waveforms in the runs (Supplementary). WS are usually seen during drowsiness and NREM sleep, but also in REM sleep [22].

#### Rhythmic mid-temporal theta burst of drowsiness

RMTD or rhythmic midtemporal discharges (previously known as “psychomotor variant”) are less frequent than WS, observed as around 2% of people undergoing EEG (Table 1) [22]. RMTD show sequence of rhythmic theta waves without slow after-wave (Fig. 1; Supplementary). They share the same location as WS but occur in longer runs, with waveforms that are usually notched but may also appear rounded or sharply contoured. RMTD are observed at drowsiness, in light NREM sleep, but also in REM sleep [11, 22].

#### Fourteen and 6-Hz positive bursts

Fourteen and 6-Hz positive bursts occur during drowsiness and light sleep, but are mainly seen during REM sleep [22]. Their topography is posterior temporal best seen using a reference montage. However, using a lower temporal line that covers the inferior part of the temporal lobe helps to detect the bursts (Supplementary) [35]. This pattern, as well as POSTs, varies with age, being less common with advancing age [35]. Fourteen and 6-Hz positive bursts consist of short trains of low-voltage waveforms with a positive polarity present at a rate of 6 Hz or less often at 14 Hz [22]. The two frequencies may be present in the same run (Fig. 1; Supplementary). Here again, there is no sequence of slow after-wave in the bursts.

#### Other normal EEG variants without slow after-wave

The rule “that most spike or sharp wave discharges of clinical import are followed by a slow wave or series of slow deflections” [24] can also be applied to mu rhythm, breach rhythm, lambda waves, spiky beta, and POSTs. Lambda waves and POSTs are monophasic or diphasic waves. POSTs may occur in repetitive wave trains in young people (Fig. 1; Supplementary). Some aspects of breach rhythms bring them closer to normal variants. The EEG activity in the area of the skull defect, or near it, is different from the remaining background activity. Waveforms are composed of higher amplitude and are sharper especially when a skull defect includes the temporal region; there can appear as runs of epileptiform waves with a morphology comparable to WS (Supplementary). Breach rhythms are considered a normal feature appropriate for the context of the recording that is usually associated with a craniotomy.

#### Normal variation of the background activity

The rule of the absence of slow after-wave can also be applied to normal variations of the background activity. The alpha rhythm can sometimes fragment at a slower frequency of 4–5 Hz, corresponding to a slow alpha variant. This is also known as a subharmonic of the alpha rhythm. Subharmonics reflect theta waves and typically have a notched morphology but without the sequence of sharp-and-wave (Fig. 1; Supplementary). Slow alpha variants can be mistaken for IEDs or seizures. More rarely, the background activity can be twice the frequency of the alpha rhythm, which constitutes a fast alpha variant. Waveforms can appear sinusoidal or notched (Fig. 1) [10]. Fast alpha variants are usually not misinterpreted as epileptiform, as they are often misinterpreted as low amplitude of the alpha rhythm.

#### Exception to the rule: small sharp spikes and 6-Hz spike-and-slow-waves

The rule of absence of slow after-wave may not apply to SSS, as they occasionally have a slow wave after the spike [17, 31] and, by definition, with 6-Hz spike-and-slow-waves. This pattern was initially termed “phantom spike and wave bursts” because of the low amplitude of the spike in contrast to the slow-wave component. However, it is unclear if this pattern must be included inside normal variants or if it indicates a trait of idiopathic (genetic) generalized epilepsy (IGE). Indeed, these bursts appear more often associated with epilepsy, especially when they predominate over the anterior rather than the posterior head regions [15]. Their interpretation depends on the clinical context. In cases where IGE is suspected, this pattern is often considered a marker of the epileptic syndrome, but in non-epileptic patients, typically when EEGs are conducted after a loss of consciousness corresponding to a vasovagal syncope, it would be considered a normal variant. In a tertiary center for epilepsy, among 1163 consecutive patients who underwent a continuous EEG for at least 24 h, Macorig et al. (2021) identified only one patient with 6-Hz spike-and-slow-wave (0.1%) [22] considerably lower compared to initial studies: 1.021% [27] and 2.79% [29] (Table 1).

### Third rule: normal EEG variants display a single non-evolving rhythm. The morphology is stable, and the same pattern repeats throughout the entire EEG recording and subsequent EEGs

Most EEG variants, such as WS, RMTD, mu rhythm, and midline theta rhythm repeat regular waveforms. They appear with the same frequency within the pattern and have non-evolving characters. However, because of the slow after-waves, patterns of 6-Hz spike-and-slow-waves and, to a lesser degree, SSS consist of two rhythms. SREDA and 14- and 6-Hz bursts may also exhibit two rhythms. In the case of 14- and 6-Hz positive bursts, these occur at a rate of either 14 Hz or 6 Hz, but it is quite usual to have the two frequencies in the same burst (Fig. 1; Supplementary). SREDA is a very rare pattern [3, 36]. It can be observed during wakefulness and activated by hyperventilation and photic stimulation, but it can also be observed in NREM and REM sleep [8, 10] (Supplementary). The pattern frequency ranges from 4 to 7 Hz and can last from a few seconds to 40–80 s [36]. SREDA can be bilateral or unilateral. The discharge onset may consist of the sudden appearance of repetitive waves or a buildup of slow waves gradually occurring at shorter intervals (Supplementary). Even if there is an evolution of the pattern, unlike an epileptic seizure, the topographic distribution of SREDA shows no buildup and the aspect remains stable throughout the entire EEG recording. Furthermore, there are no abnormal clinical findings associated.

Interictal abnormalities often fluctuate in morphology, amplitude, and topography, especially during NREM sleep. In focal epilepsies, “epileptic” discharges tend to diffuse. A new focus can appear. By definition, an epileptic seizure is represented by a low voltage cerebral activity that increases in amplitude, decreases in frequency, and spreads to adjacent regions of the brain detected on the scalp. Normal EEG variants appear monomorphic often appearing intermittently throughout the EEG tracing. They remain localized in the same brain region, and their morphology is stable repeating the same pattern throughout the entire EEG recording and often appearing in subsequent EEGs.

### Fourth rule: phase reversals merely indicate localization, not epilepsy

**Fig. 2:**
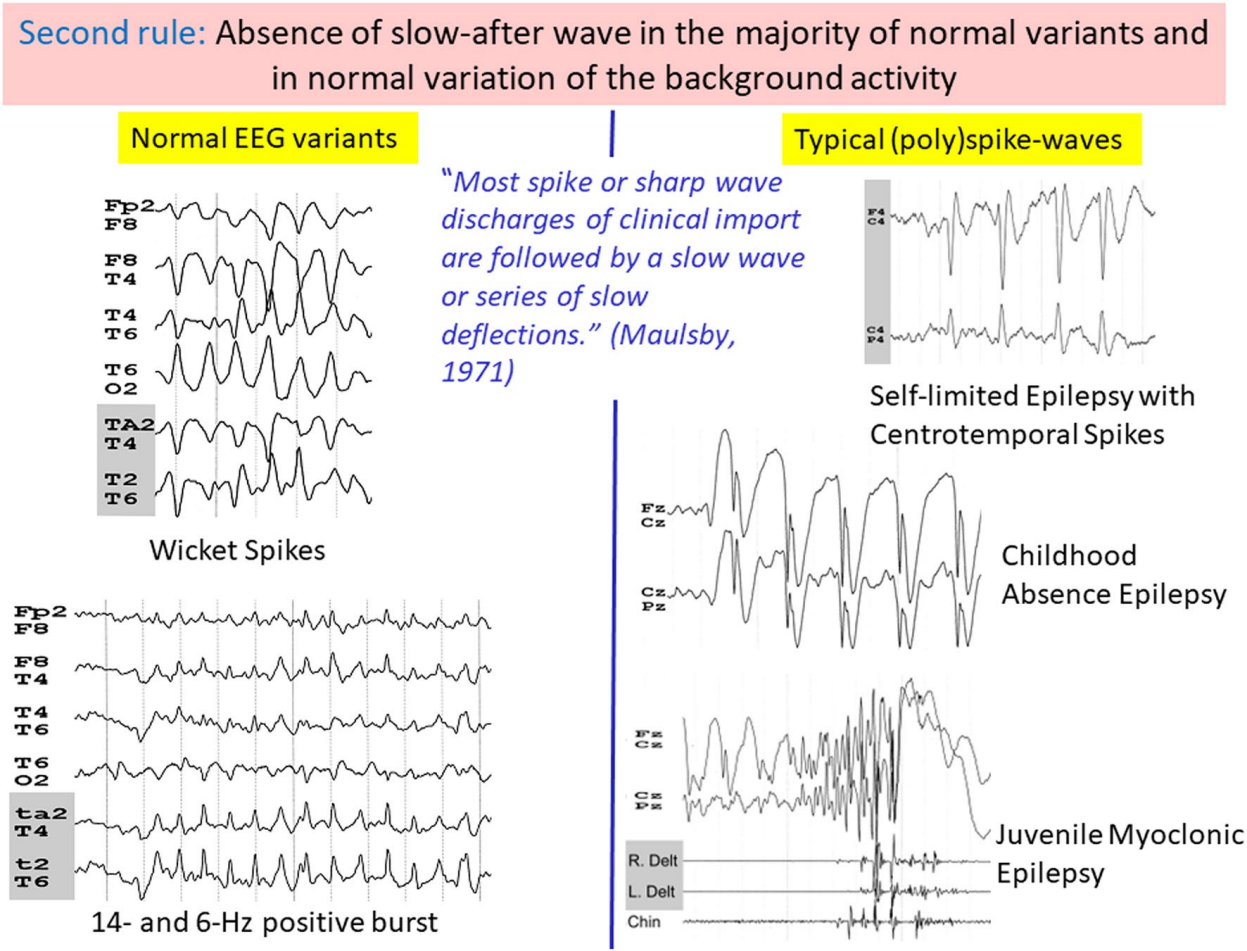
If there is no wave after the spike, neurophysiologists should be suspicious of artifacts or normal EEG variants or normal variation of the background activity (Recommendation 2)

In bipolar montages, a simultaneous deflection in opposite directions from adjacent channels is called a phase reversal. This phase reversal indicates that the electrodes are located near the generator source and help identify the localization in focal epilepsies. Because most epileptic discharges are surface-negative, negative phase reversals are one of the major pitfalls in EEG interpretation, commonly (and erroneously) believed to suggest epileptic activity [18, 23, 38]. Phase reversals are also seen with electrode artifacts and lateral eye movements with lateral rectus spikes at F7/F8 (Fig. 2). Many normal variants cause typical phase reversal. Among them, WS, RMTD, and SSS can be confused for epileptiform activity due to their temporal lobe localization.

### Fifth rule: interictal epileptiform discharges and normal EEG background variations differ in their EEG reactivity patterns

Reactivity upon eye opening and closing must be systematically tested. EEG patterns with an occipital topography that react like alpha (eye opening/closing) but also disappearing at drowsiness (see below) should be considered as a variation of the background activity. The alpha rhythm is present when the eyes are closed, but it disappears when the person is asked to open their eyes. Similarly, slow and fast alpha variants block with eye opening as well as unilateral enhanced alpha rhythm due to a skull defect. In children of 3–10 years, posterior slow waves may be intermixed with the alpha rhythm, sometimes resembling spike-waves, which can be misleading (Fig. 1; Supplementary). But, as with the alpha rhythm, posterior slow waves of youth are blocked when the eyes are open and disappear at drowsiness. There are very few epileptic syndromes in which abnormalities can react to eye opening (childhood occipital visual epilepsy and less frequently in some cases of self-limited epilepsy with autonomic seizures and epilepsy with eyelid myoclonia). In these situations, the epileptic nature of the pattern can be easily recognized with the sequence of slow after-wave.

Lambda waves occur in the occipital regions in wakefulness, when individuals are visually scanning a picture in a well-illuminated room, or when reading with scanning eye movements (Fig. 1). Their occurrence may be elicited by watching television [1] or reading on tablet [12]. They disappear when the room is not as bright or when patients stare at a fixed point (Supplementary). The mu rhythm is also reactive and commonly occurs in the central regions. It does not block with eye opening but blocks unilaterally upon contraction of the contralateral hand (Supplementary) or upon movement of the contralateral foot if the rhythm is near the vertex. Midline theta rhythm resembles mu rhythm but it does not react similarly. It consists of sinusoidal or arciform 4–7 Hz activity occurring over the vertex region (Fig. 1; Supplementary). Cognitive tasks requiring concentration may lead to the occurrence of midline theta rhythm [3] (Supplementary).

### Sixth rule: drowsiness and NREM sleep facilitate the occurrence of interictal epileptiform discharges

Sleep facilitates the occurrence of IEDs [25, 28]. This effect can be noted even for short-duration sleep. When epileptiform activity decreases or stops at drowsiness or sleep onset, electroencephalographers should consider this atypical response as uncommon for epilepsy. Muscle, tremor, or other movement artifacts disappear with drowsiness, along with the alpha rhythm and its variants, the posterior slow waves of youth. Midline theta rhythm (Ciganek’s rhythm) may be observed during wakefulness or drowsiness, but this activity disappears at sleep onset.

### Seventh rule: wicket spikes are best identified during sleep

During wakefulness, WS may be hard to identify because they have an irregular theta morphology. Even with these difficulties, there is no spike-and-wave sequence. WS are usually typical during drowsiness and sleep. The sequence of irregular theta waves over the temporal lobes during wakefulness that evolves into typical WS during sleep highlights the importance of sleep recordings in case of any doubt. The observation of typical WS during sleep allows us to associate the theta waves in wakefulness with unusual but nonpathological activity (Supplementary).

### Eighth rule: REM sleep is relatively protective against interictal epileptiform discharges and epileptic seizures

IEDs are maximally activated during NREM sleep but tend to decrease during REM sleep, offering a better localization of the discharges in focal epilepsies [25, 26]. Contrary to IEDs, 14- and 6-Hz positive bursts are more frequent during REM sleep than in NREM sleep in patients who underwent recordings of their overnight sleep [22]. This pattern can be only present in REM sleep; the bursts allow identification of REM sleep in association with its typical EEG characteristics (i.e., low-voltage tracing with sawtooth waves, beta rhythms over the anterior regions, muscle twitches, and rapid eye movement artifacts) [22].

RMTD may be observed in REM sleep but in smaller runs that cannot be confused with an epileptic seizure. SREDA, which resembles seizure activity, can be observed in REM sleep as well as in NREM sleep [8, 10] (Supplementary). The phenomenon is highly reproducible, as SREDA occurs several times during the sleep recording without clinical signs reflecting a nonepileptiform variant.

### Ninth rule: epileptic seizures usually disrupt sleep patterns

Subclinical epileptic seizures may occur in sleep (Supplementary). However, epileptic seizures usually cause arousal. In case of no behavioral arousal, electroencephalographers should look for subtle changes in heart rate, respiration, and alpha rhythm recovery after the discharge ends. If there is no arousal, be more suspicious of electrode artifacts and among normal EEG variants, RMTD and SREDA, especially when physiological elements of sleep such as K complexes are present inside the discharge (Supplementary). RMTD may resemble a focal temporal seizure but unlike an epileptic seizure, which would disrupt drowsiness, patients fall asleep after RMTD and do not wake up if they are sleeping (Supplementary). SREDA occurring in NREM or REM sleep also does not disrupt sleep (Supplementary).

### Tenth rule: epilepsy is and must remain a clinical diagnosis

EEG is foundational in diagnosing and managing patients with epilepsies [33]. In the absence of semiological evidence, a patient should not be diagnosed as “epileptic” based solely on observation of epileptiform abnormalities. Misdiagnosing patients with epilepsy has serious consequences including adverse effects on driving and transportation, employment, financial implications, and adverse psychosocial ramifications. Before attributing pathological significance to normal EEG features that are benign, it is advisable to be conservative when in doubt. Complementary assessment with video-EEG recording including EEG that contains sleep may resolve questionable features from standard EEG recordings. In many situations, recording smartphone videos can be helpful for accurate diagnosis, especially in distinguishing epileptic seizures from nonepileptic events [2, 32]. As previously stated in the introduction, epileptiform does not imply a pathological condition, including epilepsy. Furthermore, typical focal or generalized spike-waves may be observed even in the absence of epilepsy (Supplementary). The clinical context remains the most paramount in the diagnosis of epilepsy.

## Recommendations for the assessment of normal EEG variants and variation of the background activity

When the alpha rhythm is ample, all physiological and unusual waveforms are accordingly ample (**Rule 1**). In consequence, when reading EEGs, neurophysiologists must not be misled by the EEG pattern’s unusual amplitude (**Recommendation 1**). Providing accurate diagnosis requires remaining vigilant in the face of high-epileptiform waves when the alpha rhythm is ample.

“Most Spike or sharp wave discharges of clinical import are followed by a slow wave or series of slow deflections”. This fundamental rule to distinguish IEDs from artifacts can also be applied to epileptiform variants (**Rule 2**). The International Federation of Clinical Neurophysiology proposed a set of six operational criteria to identify IEDs [17, 20]. One of them includes the presence of a slow wave after epileptiform transients. This fundamental rule of slow after-wave allows us to understand that the typical pattern of epilepsy is the spike-and-wave. If there is no wave after the spike, neurophysiologists should be suspicious of artifacts or normal EEG variants/normal variations of the background activity (**Recommendation 2**).

Normal EEG variants display a single non-evolving rhythm. The morphology is stable with repetition of the same pattern throughout the entire EEG recording and subsequent EEGs (**Rule 3**). Therefore, neurophysiologists should be suspicious of possible EEG variants when the pattern appears monomorphic and repeats itself identically throughout the recording (**Recommendation 3**).

Phase reversals merely indicate localization, not epilepsy (**Rule 4**). Electroencephalographers must know the basic principles of polarity in EEG and source localization. The phrase “phase reversal” in an EEG report should be only used to describe the localization of the pattern and should not imply epileptogenicity or abnormality (**Recommendation 4**).

IEDs and EEG background variations differ in their EEG reactivity patterns (**Rule 5**). Patterns that react similarly to the alpha rhythm correspond to alpha-harmonic or posterior slow waves of youth (**Recommendation 5**).

Drowsiness and NREM sleep facilitate the occurrence of IEDs (**Rule 6**). Neurophysiologists need to be vigilant to artifacts (muscle, movement) or normal variation of the background activity and Ciganek's rhythm when the activity decreases or stops at drowsiness or sleep onset (**Recommendation 6**).

WSs are best identified during sleep (**Rule 7**). Therefore, sleep EEGs play a crucial role in resolving challenging problems. In case of doubt or difficulties, we recommend obtaining an EEG that includes sleep stages (**Recommendation 7**).

REM sleep is relatively protective against IEDs and epileptic seizures (**Rule 8**). Electroencephalographers should be suspicious of normal variants (14- and 6-Hz positive bursts) when activity increases in REM sleep or when rhythmic discharges are observed in REM sleep without arousal (**Recommendation 8**).

Seizures may disrupt sleep patterns (**Rule 9**). In case of subclinical discharges at drowsiness and during sleep, neurophysiologists must consider electrode artifacts, RMTD, and SREDA (**Recommendation 9**).

Finally, epilepsy is and must remain a clinical diagnosis (**Rule 10**). Therefore, EEG must remain a tool used to confirm clinical hypotheses (**Recommendation 10**).

## Conclusion

Distinguishing normal EEG variants from other epileptiform abnormalities is crucial to avoid overinterpretation and subsequently overdiagnosing epilepsy in clinically doubtful cases. Their identification mainly depends on the recording techniques, the length of the sleep recording, and the team's level of expertise. It is not easy to list all EEG activities accurately, but they can be recognized by their monorhythmic feature (single rhythm), the repetition of the same pattern throughout the entire EEG recording and by the fact that interictal epileptiform abnormalities are followed by a slow after-wave (spike-and-wave). In case of difficulties, repeating a sleep EEG or prolonged overnight recording may play a crucial role in resolving challenging waveforms.

### Supplementary Information

Below is the link to the electronic supplementary material.

Supplementary file1 (PDF 19606 KB)

## Data Availability

Not applicable.
