# Supplementary material for: Overcoming traps and pitfalls leading to misinterpretation of normal EEG variants and variation of the background activity
Source: J Neurol. 2024 May 18;271(7):3869–78. doi: 10.1007/s00415-024-12440-y (PMC11233371; doi:10.1007/s00415-024-12440-y)
Supplement: Supplementary file 1 — Supplementary file1 (PDF 19606 KB) [file 415_2024_12440_MOESM1_ESM.pdf]

Traps and Pitfalls Leading to Misinterpretation of Normal EEG Variants and Variation of the Background Activity

Philippe Gélisse[1,2], Selim Benbadis[3], Arielle Crespel[1,2], and William O. Tatum[4]

[1]Gui de Chauliac Hospital, Epilepsy Unit, Montpellier, France.

[2]Research Unit (URCMA: Unité de Recherche sur les Comportements et Mouvements Anormaux), INSERM, U661, Montpellier, France

[3]University of South Florida, Department of Neurology, Tampa, Florida, USA.

[4]Department of Neurology, Mayo Clinic College of Medicine and Health Sciences, Jacksonville, Florida, USA.

Additional electrodes

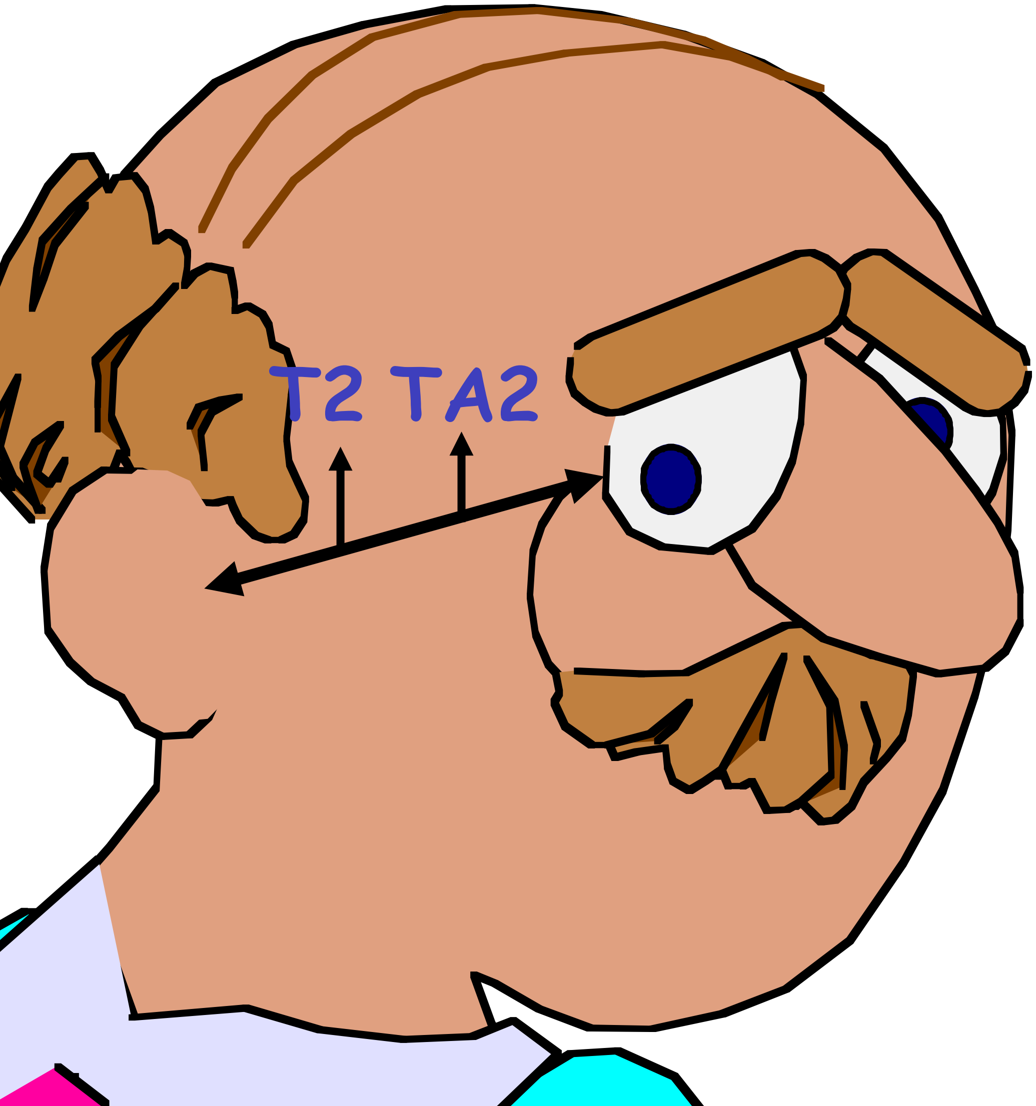

- The lower temporal line is measured from the external auditory canal to the lateral canthus of the eye.
- The line is divided by three.
- T1 and T2 are marked at one-third from the external auditory canal.
- TA1 and TA2 are marked at one-third from the lateral canthus of the eye.
- The electrodes are placed one centimeter above these points.
- T1 and T2 are the zygomatic electrodes.
- TA1 and TA2 are the anterior lower temporal electrodes (Temporal Anterior).

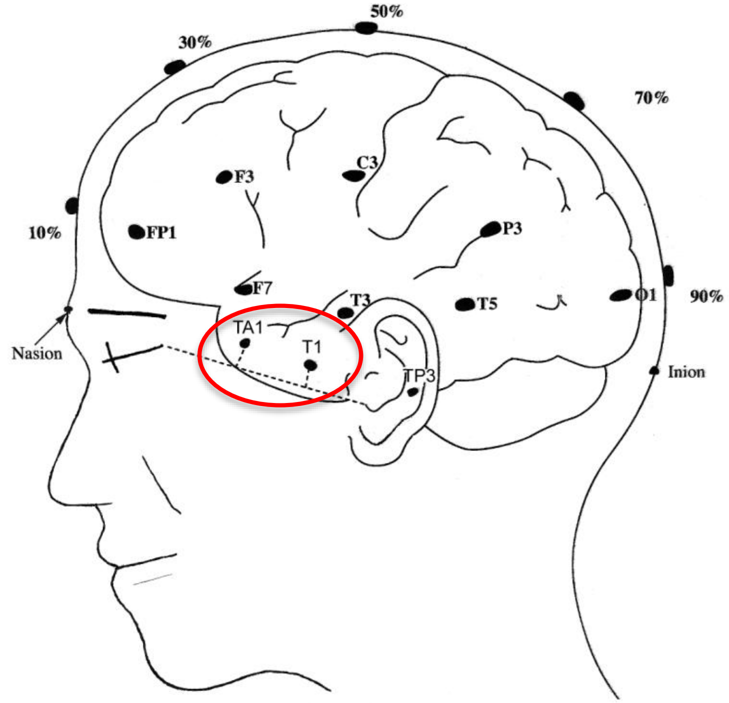

Right suprasylvian electrodes →

Fp2 F4
F4 C4
C4 P4
P4 O2

Right temporal lobe →

Fp2 F8
F8 T4
T4 T6
T6 O2

Right lower temporal electrodes →

TA2 T4
T2 T6

Vertex →

Fz Cz
Cz Pz

Left lower temporal electrodes →

TA1 T3
T1 T5

Left suprasylvian electrodes →

Fp1 F3
F3 C3
C3 P3
P3 O1

Left temporal lobe →

Fp1 F7
F7 T3
T3 T5
T5 O1

ECG →

ECG

The Context of Recording EEG

Frequency (Hz)	Normal	Abnormal
**0.0 - 0.5 (Infraslow activity)**	Artifacts	Ictal onset focal seizures
**0.5 - 3.5 (Delta)**	PSWY, HV, N3 Sleep, elderly	Encephalopathy, white matter lesion
**>3.5 - <8.0 (Theta)**	Drowsiness, children, elderly	Encephalopathy, white matter lesion
**8 – 13 (Alpha)**	PDR, mu rhythm, "third" rhythm	Ictal rhythm in seizure, alpha coma
**13 – 30 (Beta)**	Medication (i.e., barbiturates & benzodiazepines, drowsiness	Breach rhythm, drug overdose, ictal rhythm
**30 – 80 (Gamma)***	Voluntary motor movement, learning & memory	Ictal onset focal seizures- intracranial EEG
**80 – 250 (Ripples)***	Cognitive processing & memory consolidation	Ictal onset focal seizures, possibly reflects epileptogenesis
**250 – 500 (Fast ripples)***	?	Ictal onset focal seizures- intracranial
**500 – 1000 (very fast ripples)***	Acquisition of sensory information	Ictal onset focal seizures- research

* = Expanded frequencies currently under investigation; HV= hyperventiliation; PSWY= posterior slow waves of youth; PDR= posterior dominant rhythm. Adapted from Tatum WO, Freund B, Feyissa A. EEG in Epilepsy September2023. https://www.medlink.com/articles/eeg-in-epilepsy

Rules and recommendations

First rule: When the alpha rhythm is ample, all physiological and unusual waveforms are accordingly ample.

→ *First recommendation: When the alpha rhythm is ample, electroencephalographers must not be misled by the EEG pattern's unusual amplitude.*

Second rule: Absence of slow after-wave in the majority of normal variants and variation of the background activity.

→ *Second recommendation: If there is no wave after the spike, electroencephalographers should be suspicious of artifacts and normal EEG variants/variations of the background activity.*

Third rule: Normal EEG variants display a single non-evolving rhythm. The morphology is stable with repetition of the same pattern throughout the entire EEG recording and subsequent EEGs.

→ *Third recommendation: Electroencephalographers should be suspicious of possible EEG variants when the pattern appears monomorphic and repeats itself identically throughout the recording*

Fourth rule: Phase reversals merely indicate localization, not epilepsy.

→ *Fourth recommendation: The phrase "phase reversal" in an EEG report should be only used to describe the localization of the pattern and should not imply epileptogenicity or abnormality.*

Fifth rule: Interictal epileptiform discharges and EEG variations differ in their EEG reactivity patterns

→ Fifth recommendation: *EEG patterns that react similarly to the alpha rhythm correspond to alpha-harmonic or posterior slow waves of youth.*

Sixth rule: Drowsiness and NREM sleep facilitate the occurrence of IEDs.

→ Sixth recommendation: *When the activity decreases or stops at drowsiness or sleep onset, electroencephalographers need to be vigilant to artifacts, variations of the background activity and Ciganek's rhythm.*

Seventh rule: Wicket spikes are best identified during sleep.

→ Seventh recommendation: *Obtain an EEG that includes sleep stages in case of doubt and difficulties.*

Eighth rule: REM sleep is relatively protective against interictal epileptiform discharges and epileptic seizures.

→ Eighth recommendation: *Electroencephalographers should be suspicious of EEG variants when activity increase in REM sleep or when rhythmic discharges are observed without arousal.*

Ninth rule: Seizures usually disrupt sleep patterns.

→ Ninth recommendation: *Consider electrode artifact, RMTD, and SREDA when the discharge occurring at drowsiness or during sleep is strictly subclinical.*

Tenth rule: Epilepsy is and must remain a clinical diagnosis.

→ Tenth recommendation: *EEG must stay a tool used to confirm clinical hypotheses.*

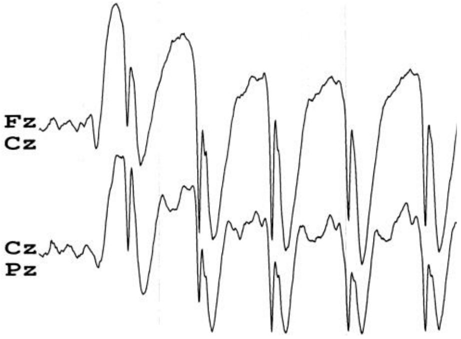

Spike-waves.

Childhood absence epilepsy.

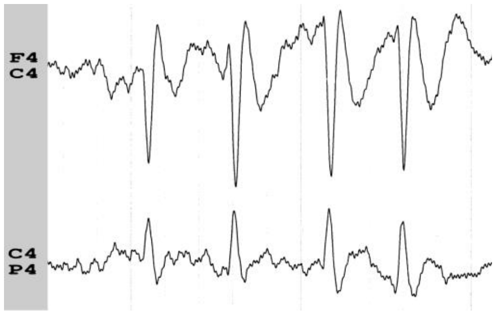

Spike-waves.

Self-limited epilepsy with centrotemporal spikes.

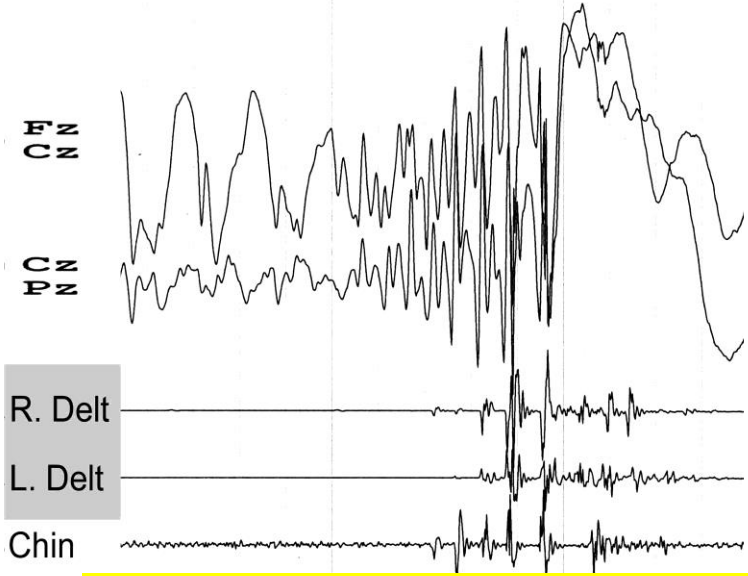

Spike-waves and polyspike-waves.

Juvenile myoclonic epilepsy,

- *"Most spike or sharp wave discharges of clinical import are followed by a slow wave or series of slow deflections.*
- *If it does not have a slow after-wave, be more suspicious of artifact or of a sudden alteration in voltage of physiological background rhythms"* .

Maulsby RL. Some guidelines for assessment of spikes and sharp waves in EEG tracings. Am J EEG Technol 1971; 11:3-16. http://dx.doi.org/10.1080/00029238.1971.11080808

Second rule: Absence of slow-after wave in the majority of normal variants and in normal variation of the background activity

Normal EEG variants

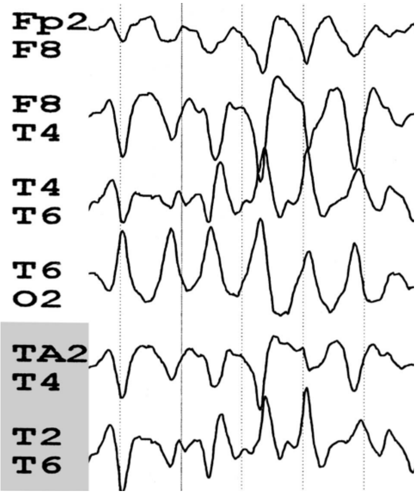

Wicket spikes

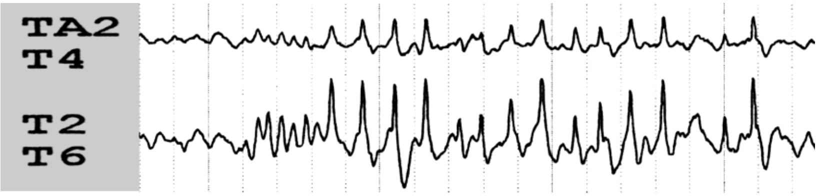

14- and 6-Hz burst

"Most spike or sharp wave discharges of clinical import are followed by a slow wave or series of slow deflections."

Typical spike-waves

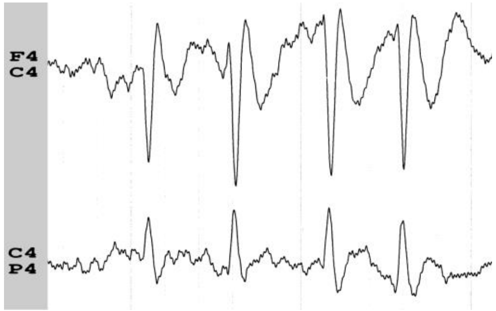

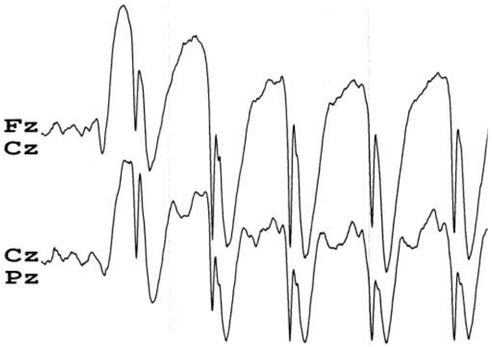

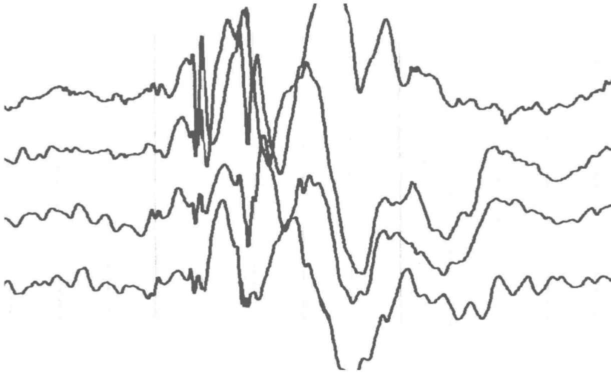

Posterior slow waves of youth

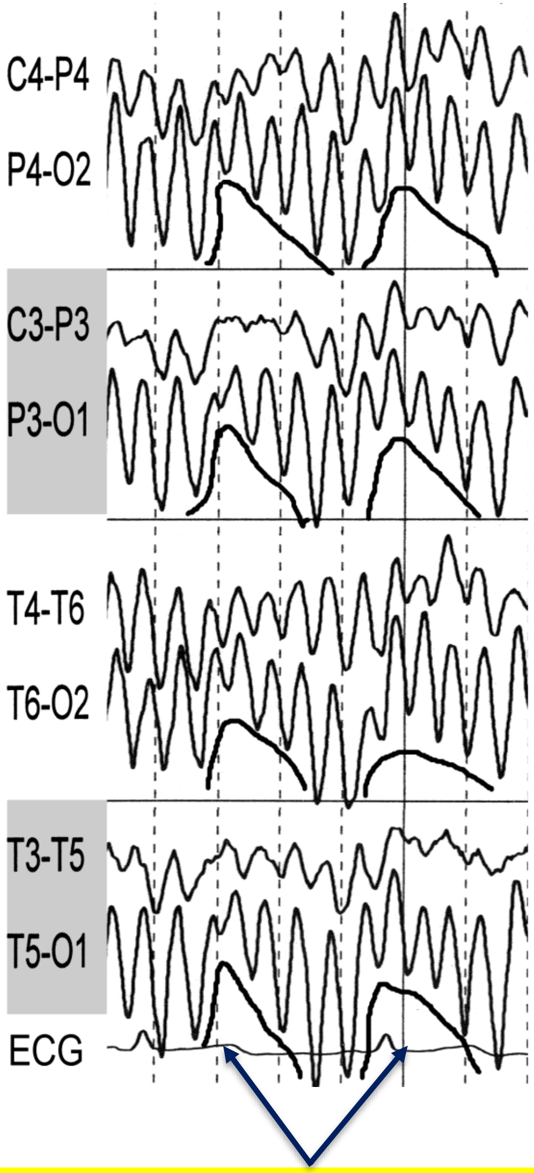

Superposition of alpha rhythm and posterior slow waves of youth (underlined)

- Bilateral
- Intermixed with the alpha rhythm
- Blocked when eyes are open
- Disappear at drowsiness
- Increase during hyperventilation

Posterior slow waves of youth

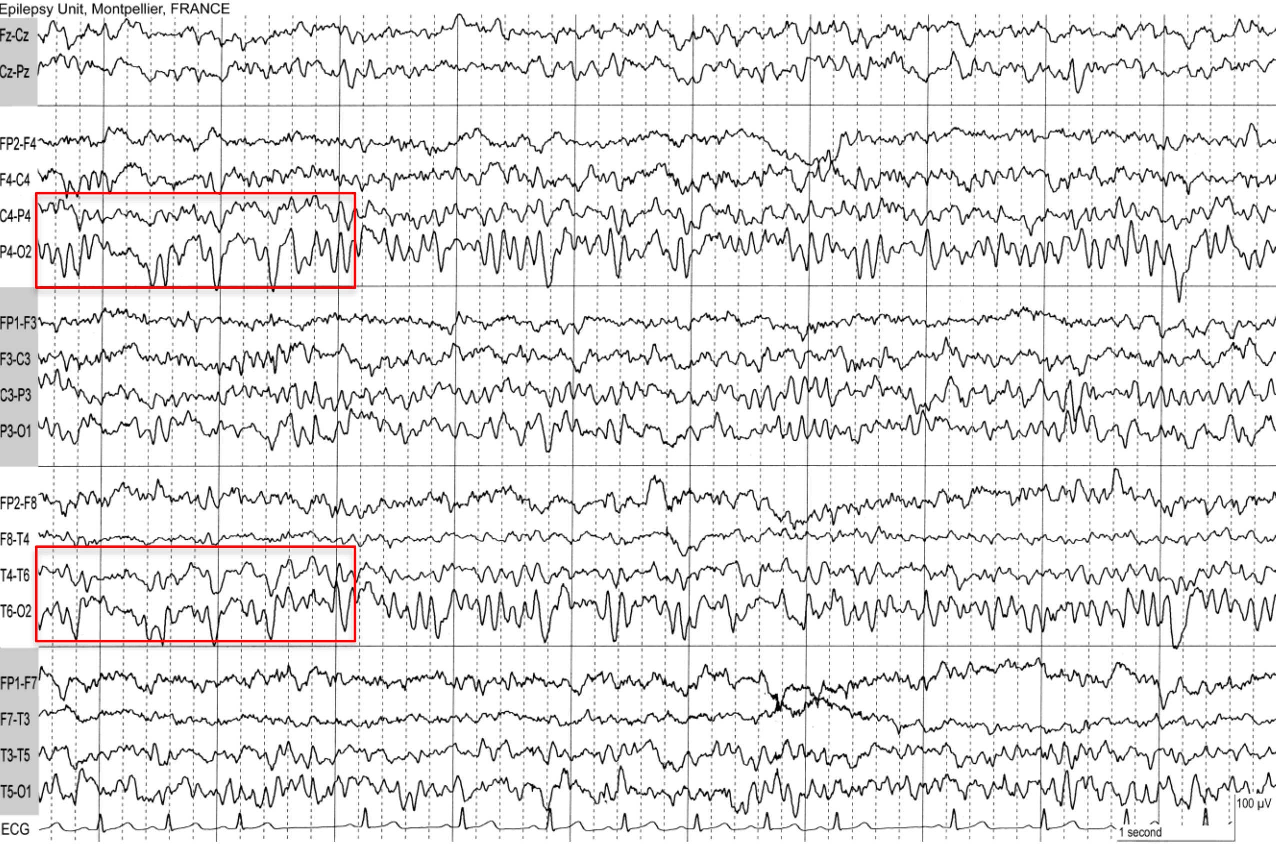

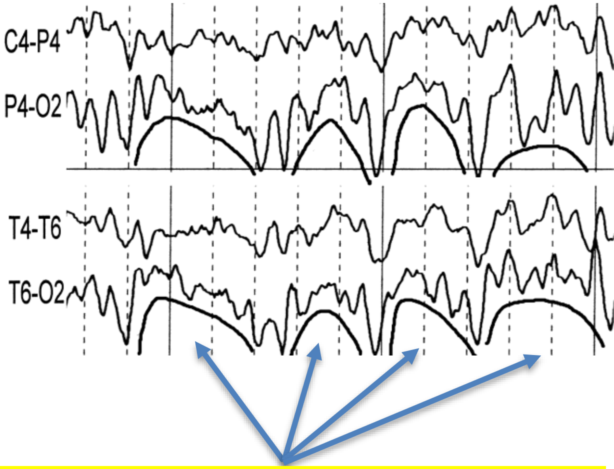

Superposition of alpha rhythm and posterior slow waves of youth (underlined)

Posterior slow waves of youth share the same reactivity with the alpha rhythm. They are blocked when eyes are open and disappear at drowsiness

Background activity: children versus adults

9-year-old child

Hyperventilation

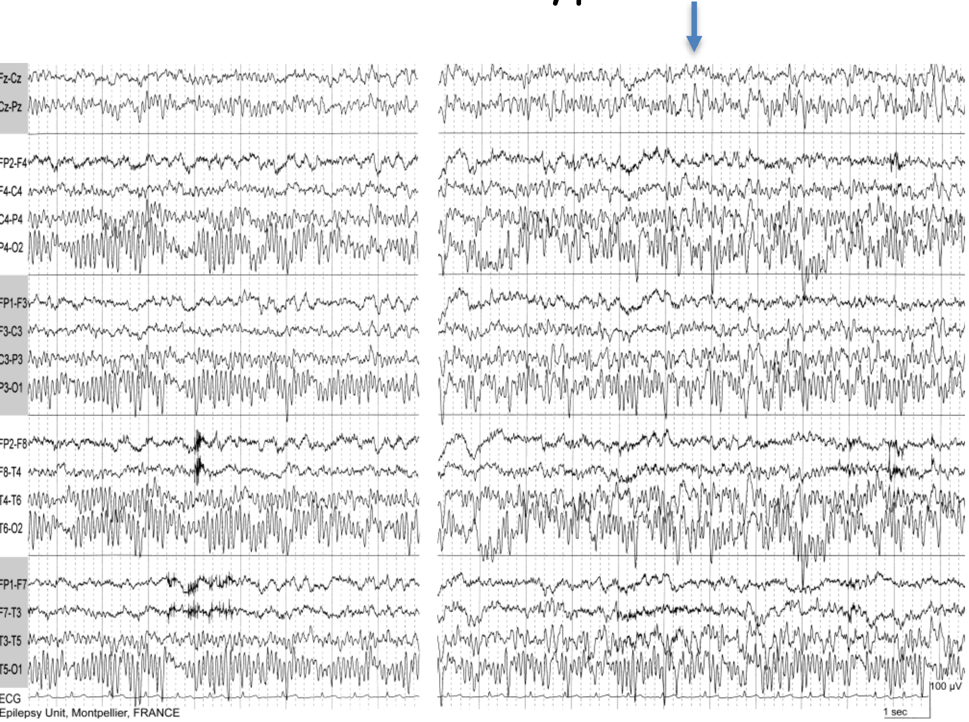

43-year-old woman

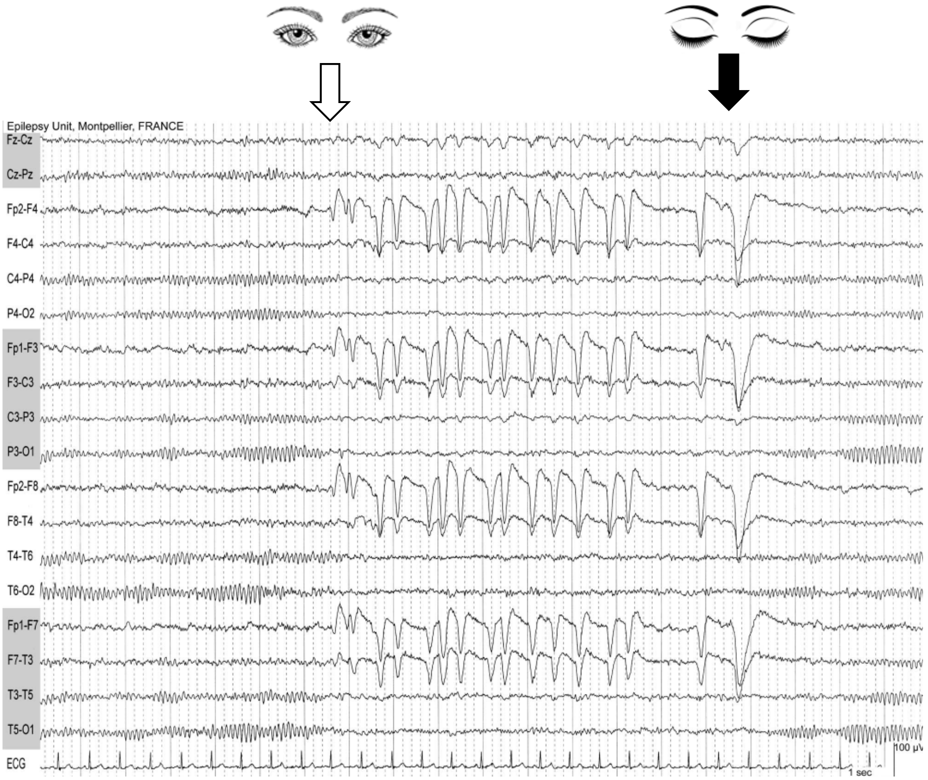

Alpha rhythm intermixed with slow posterior slow waves presenting sharply-contoured waves that interrupt the sinusoidal pattern of the alpha rhythm.

Posterior slow waves are significantly enhanced by hyperventilation

Note the reactivity of the alpha rhythm upon eye opening and eye closure

Slow alpha variant

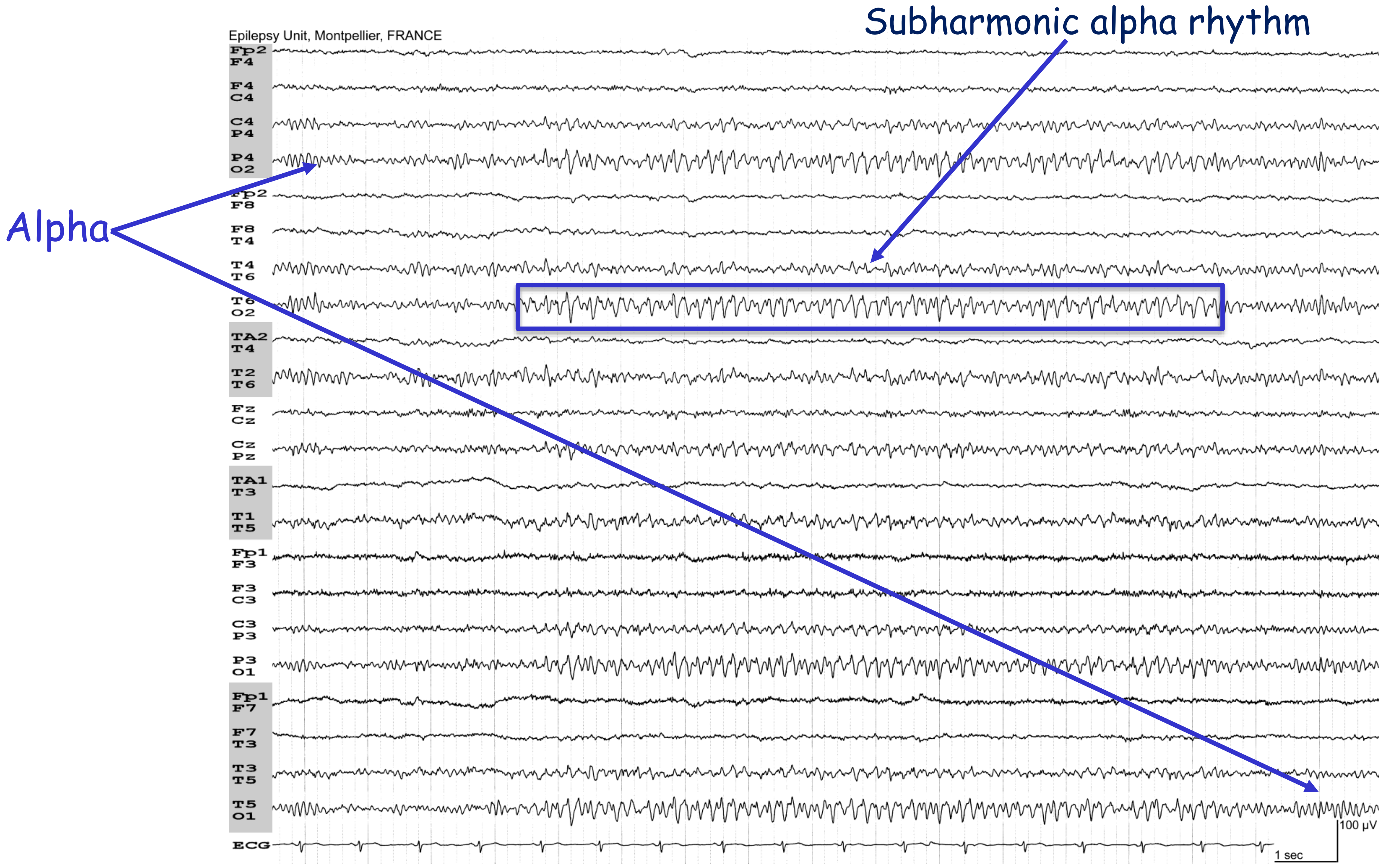

49-year-old woman

Same EEG panel at an EEG speed of 30 mm/s

Slow alpha variant

49-year-old woman

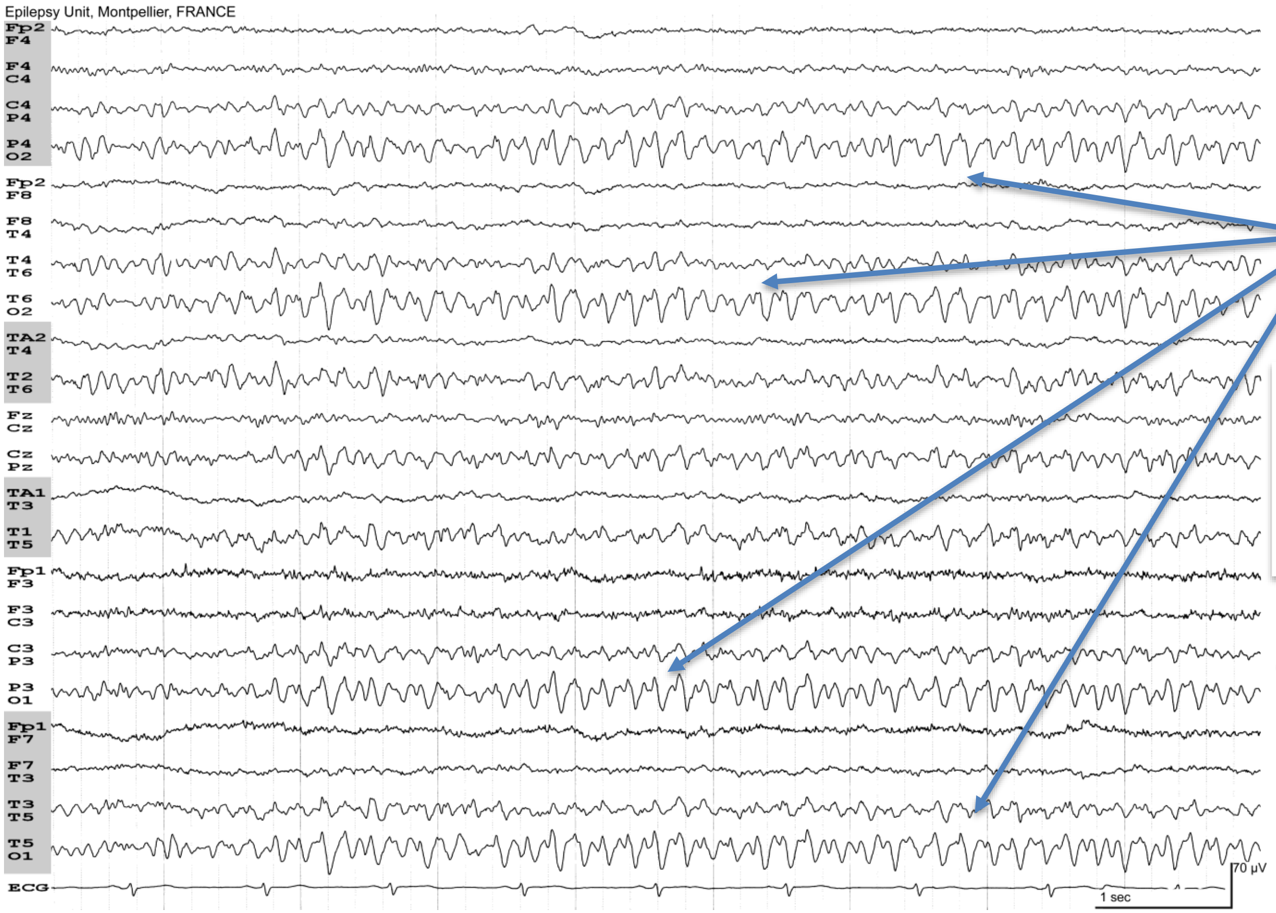

Subharmonic alpha rhythm

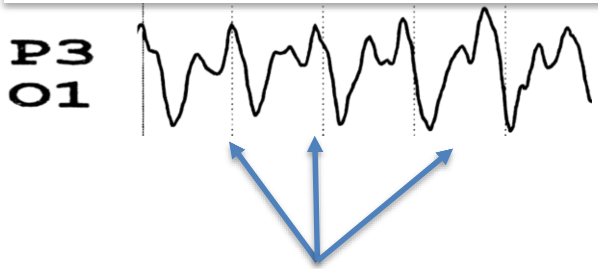

The waves are clearly forked

Slow alpha variant shares the same reactivity with the alpha rhythm. It is blocked when eyes are open and disappear at drowsiness

Slow alpha variant

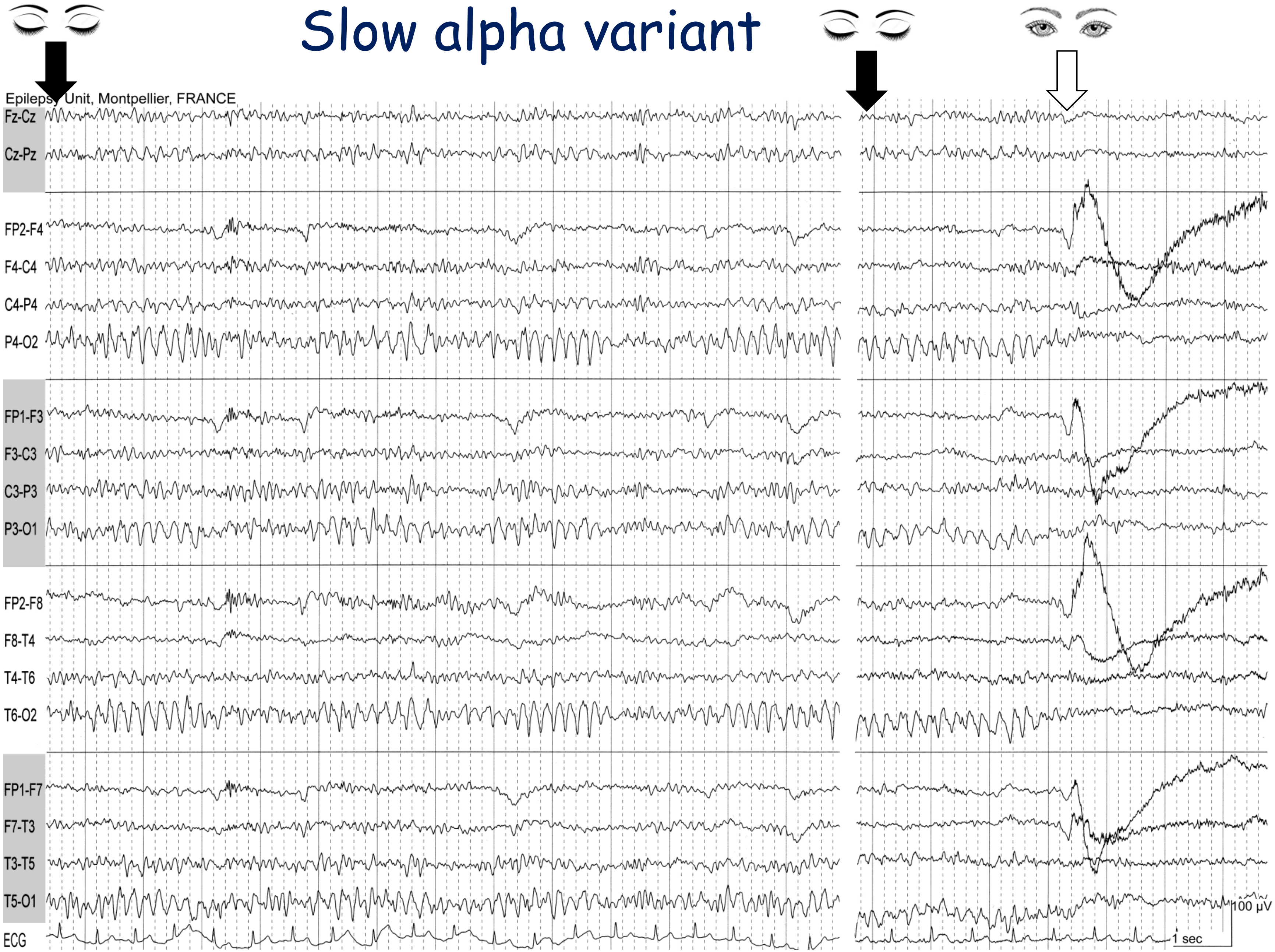

52-year-old woman

The rhythm disappears upon eye opening

52-year-old woman

Slow alpha variant

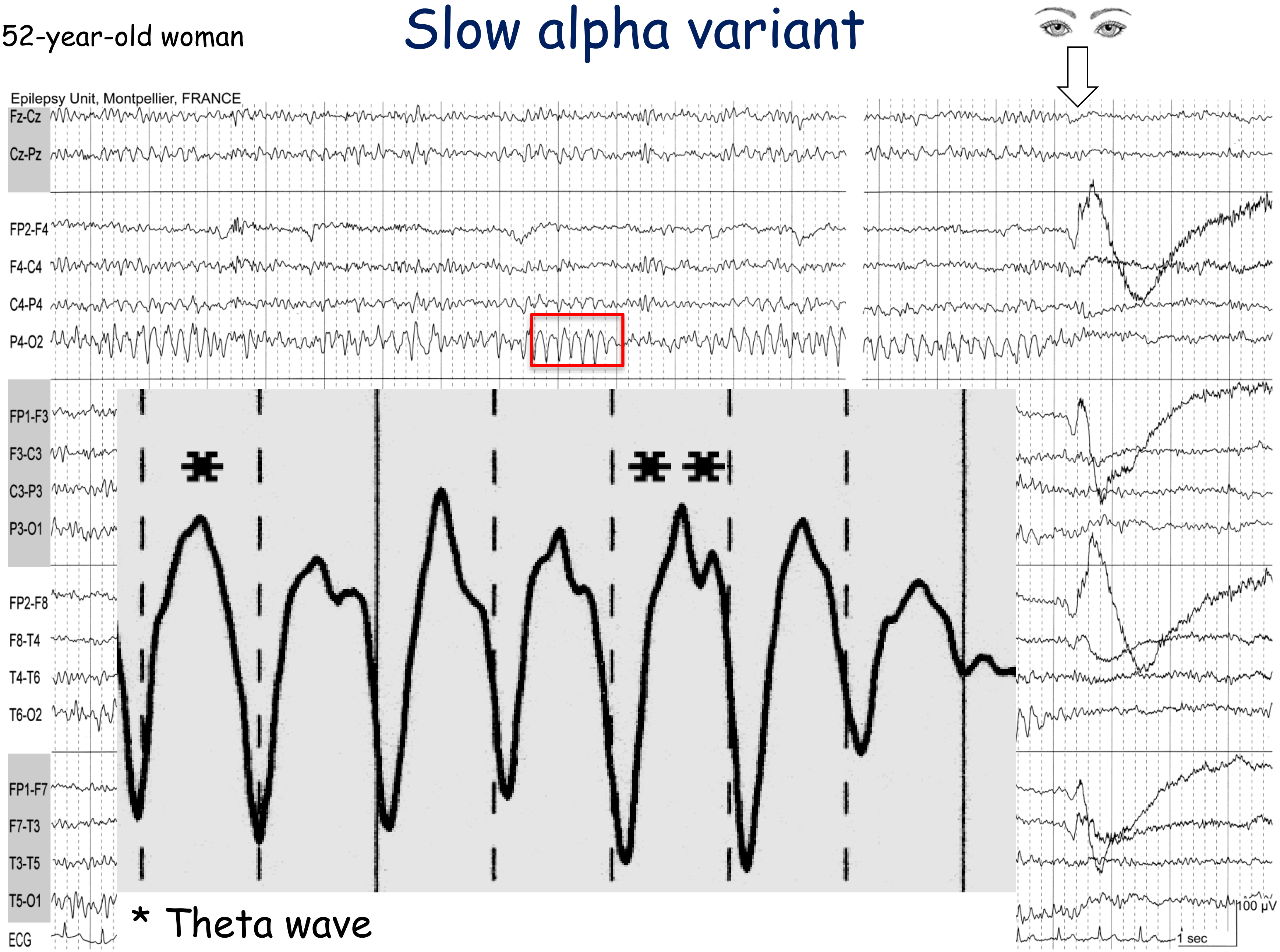

* Theta wave

**Notched theta wave (bifid aspect)

The rhythm disappears upon eye opening

Mu rhythm

- Observed during resting wakefulness but can also be observed during sleep, especially REM sleep.
- Mu rhythm commonly occurs over the central region at 7-11 Hz.
- Mu rhythm blocks unilaterally upon contraction of the contralateral hand, or upon movement of the contralateral foot if the rhythm is near the vertex.

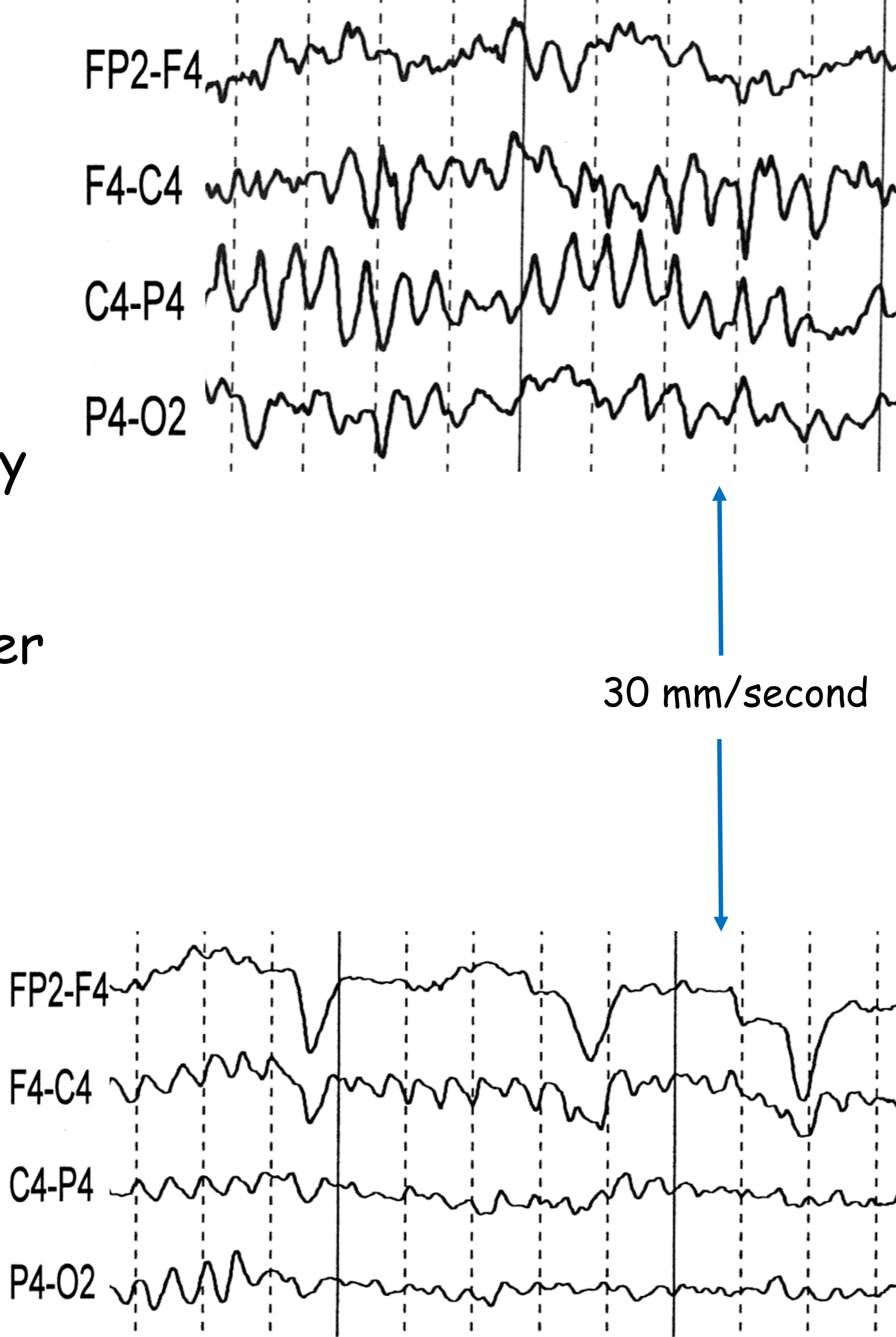

Mu rhythm

12-year-old woman. Migraine

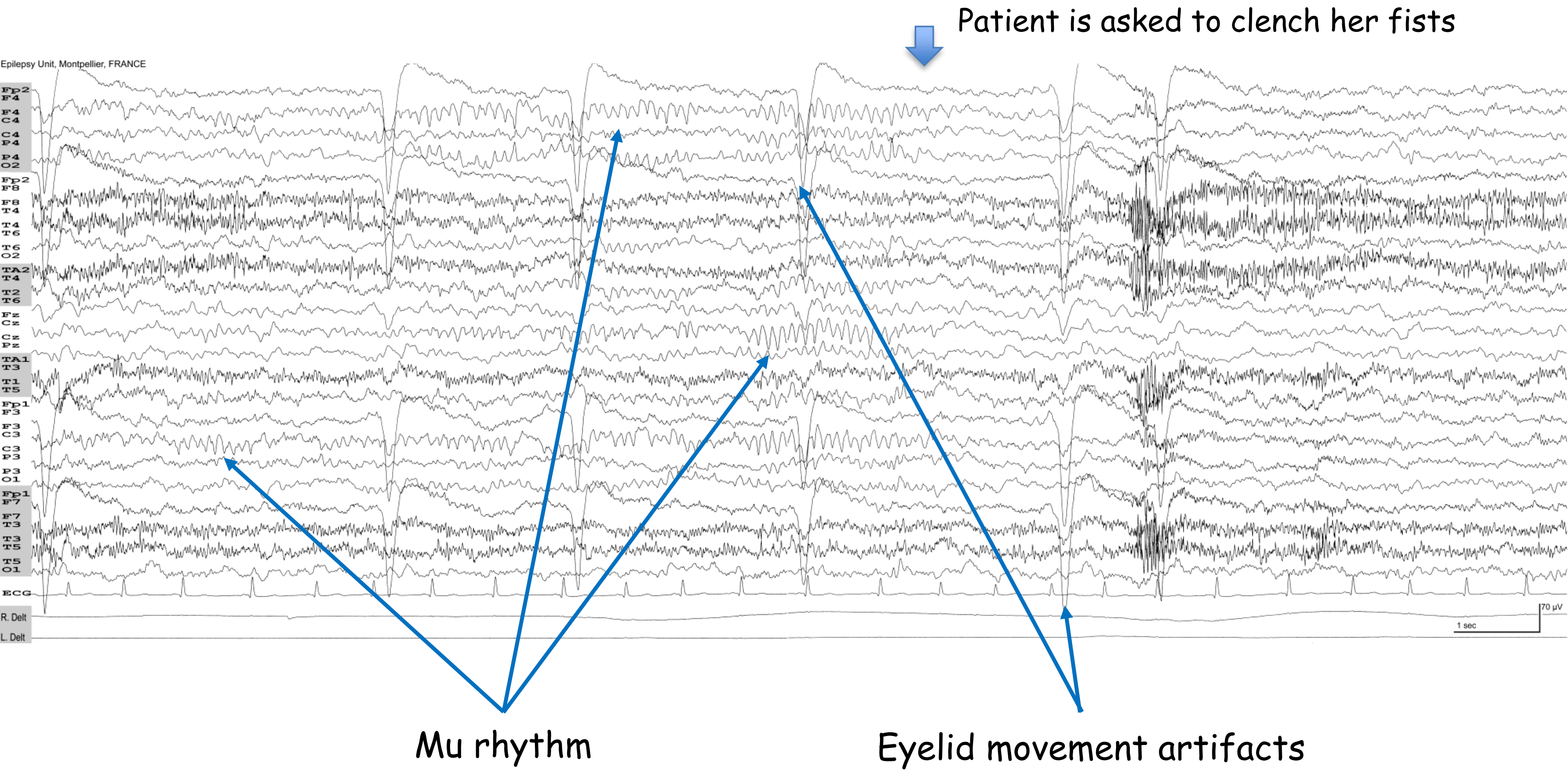

Patient is asked to clench her fists

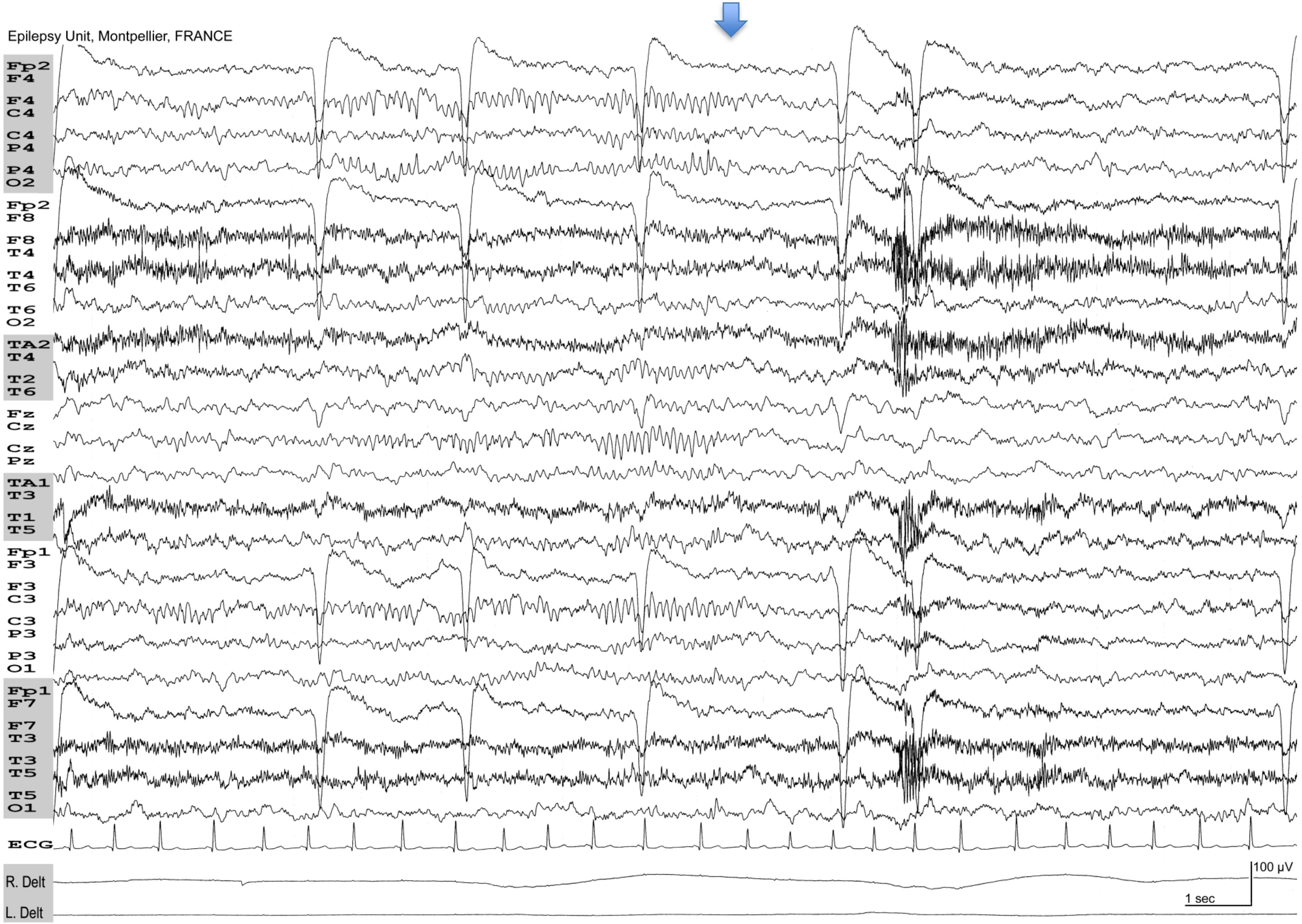

Same EEG panel at an EEG speed of 15 mm/s

Mu rhythm in REM sleep

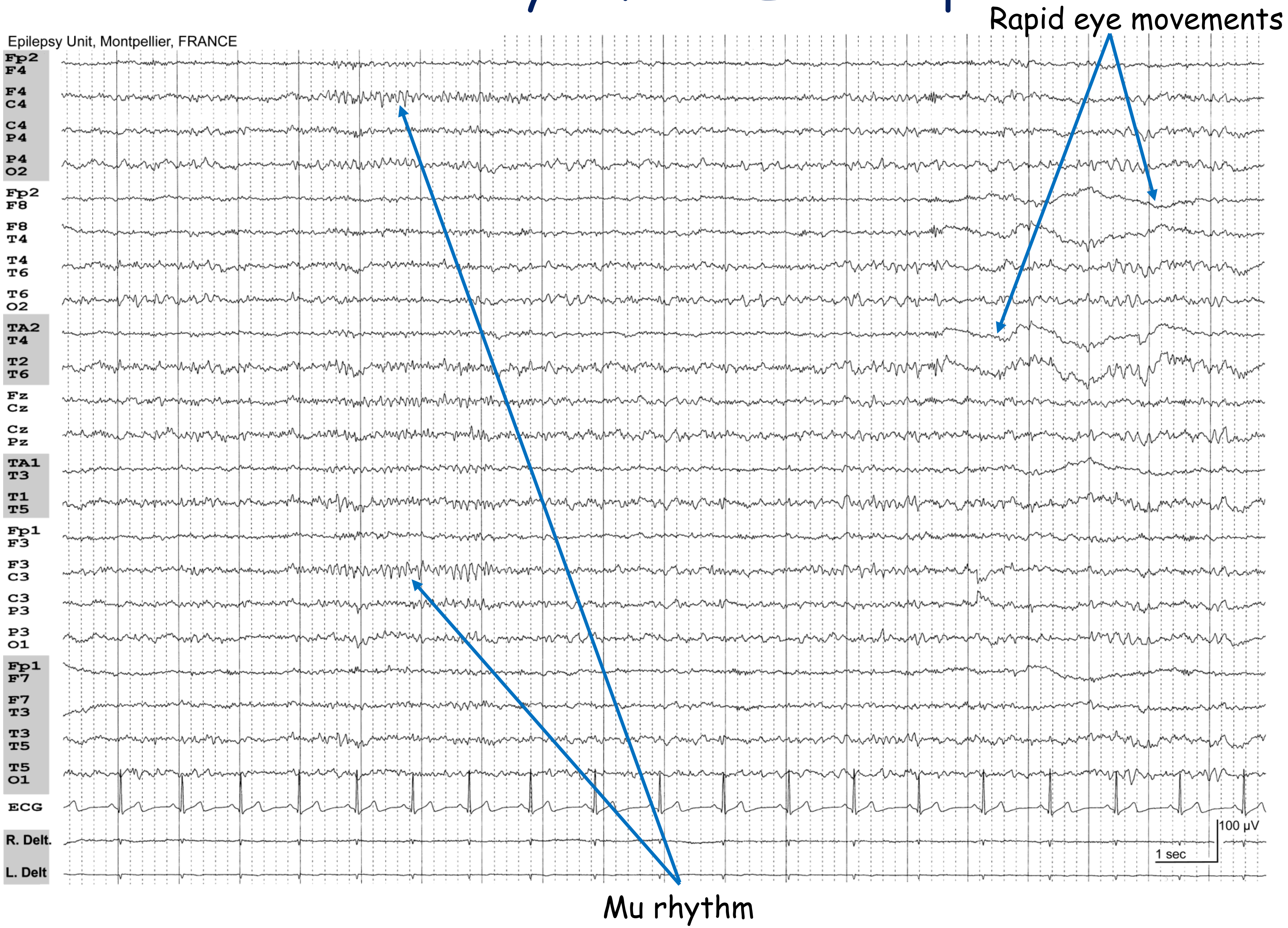

42-year-old woman

Slow mu variant

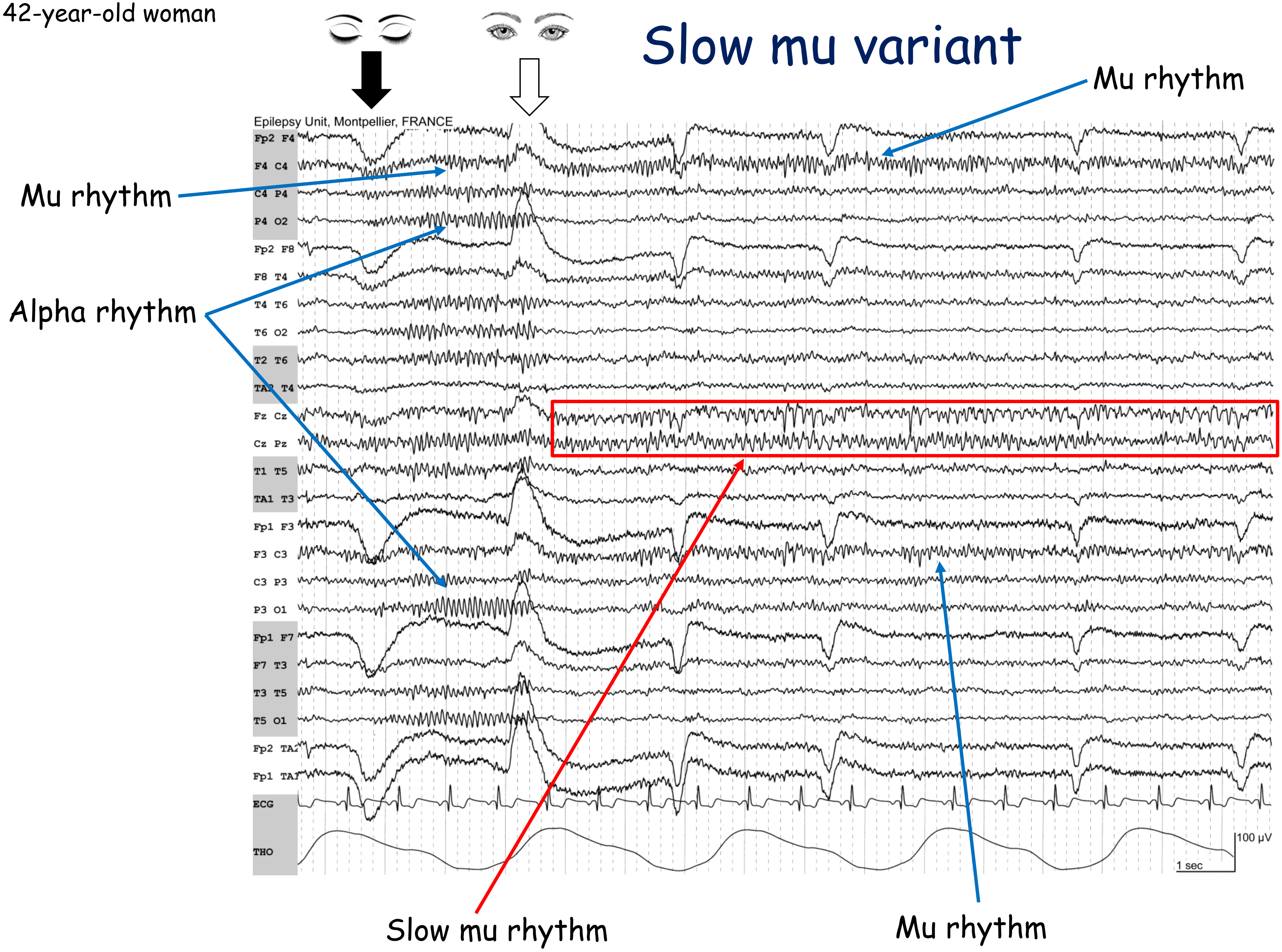

Midline theta rhythm (Cigánek rhythm)

- Observed during wakefulness or drowsiness.
- Sinusoidal or arciform 4-7 Hz activity.
- This rhythm occurs predominantly over the vertex (Cz).
- Sometimes, this rhythm resembles mu rhythm but it is not similarly reactive.

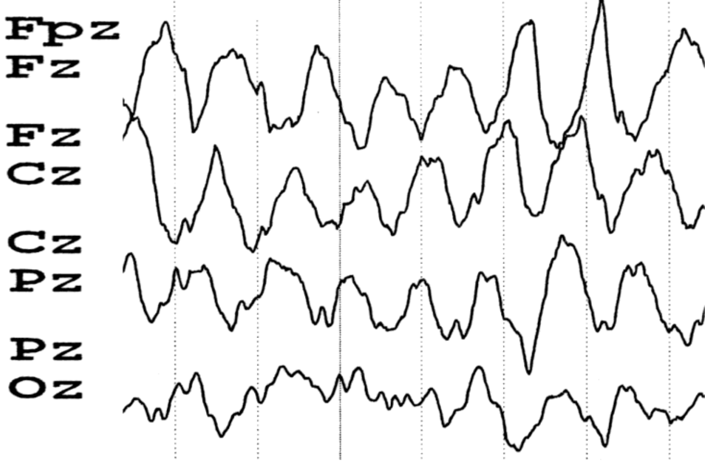

30 mm/second

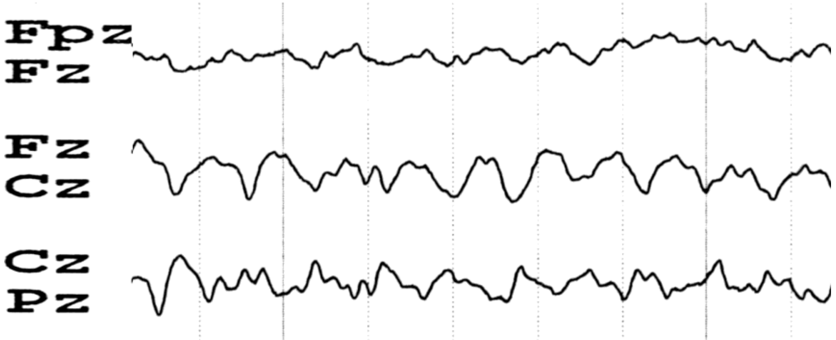

Midline theta rhythm (Cigánek rhythm)

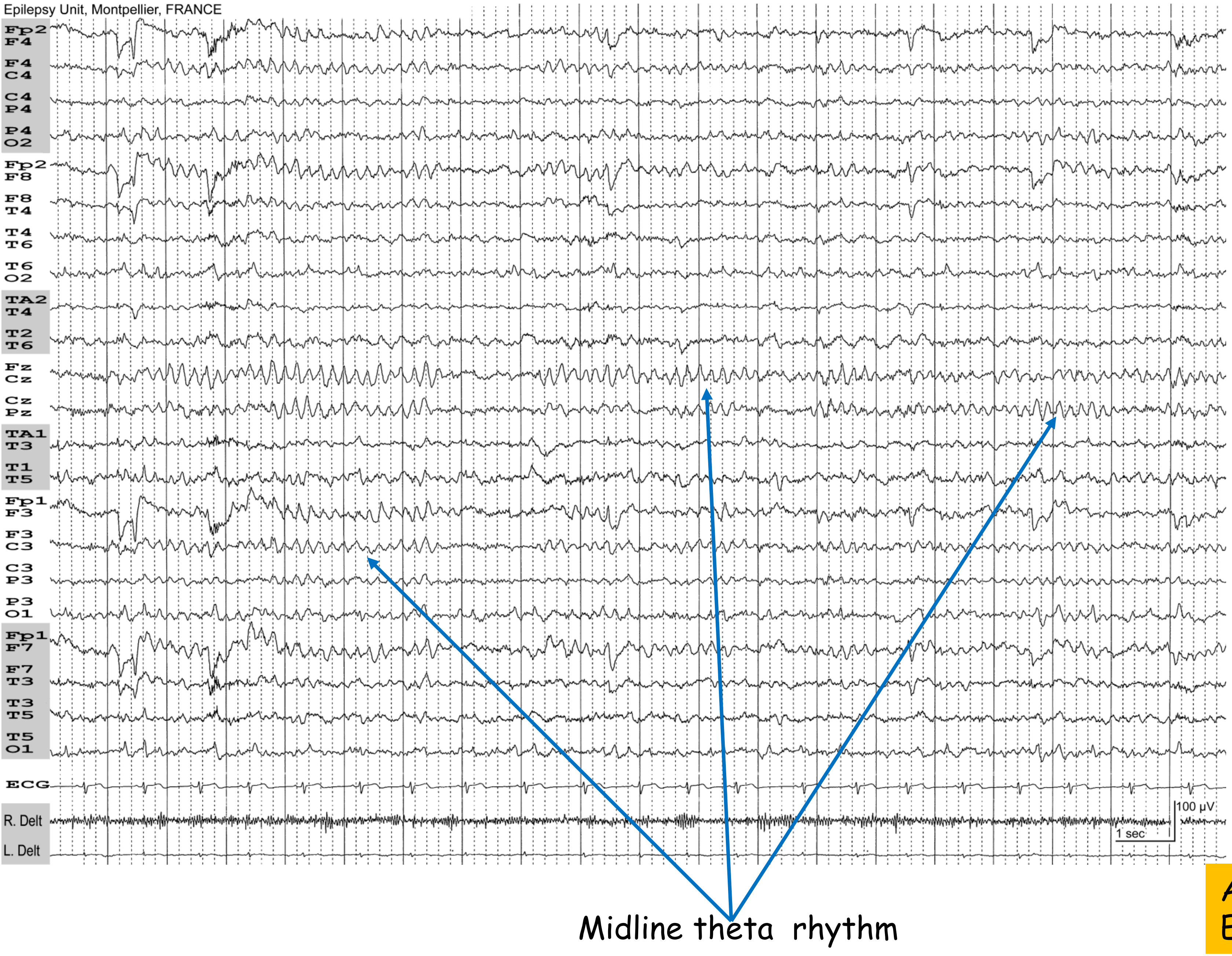

Awake
Eyes open

Midline theta rhythm and mu rhythm in the same patient

Awake
Eyes open

18-year-old woman.
Parasomnia

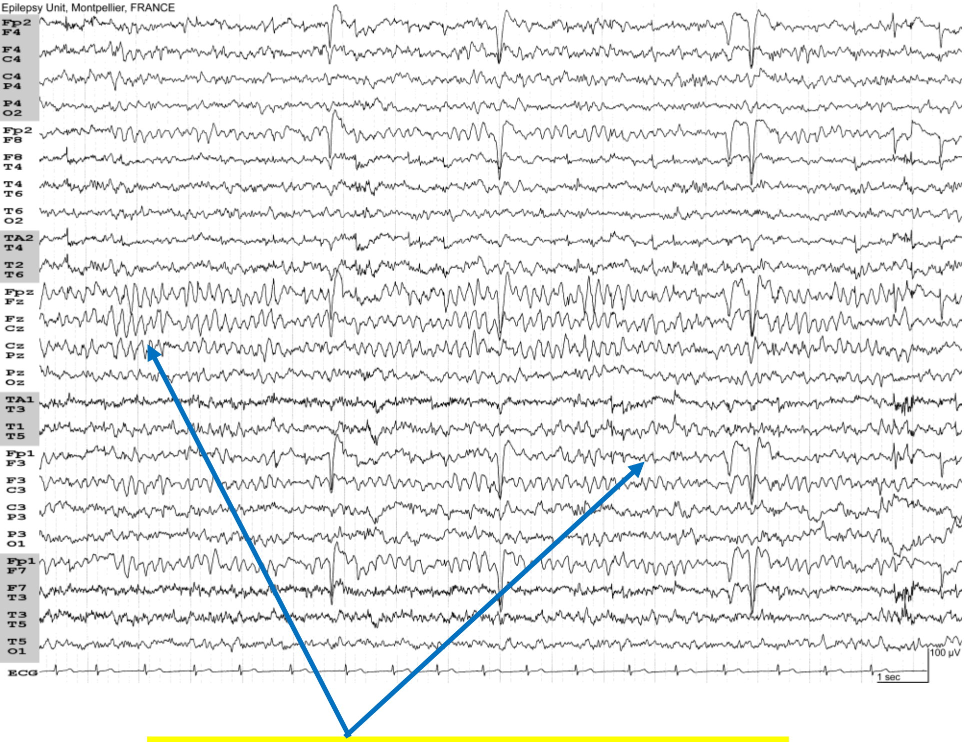

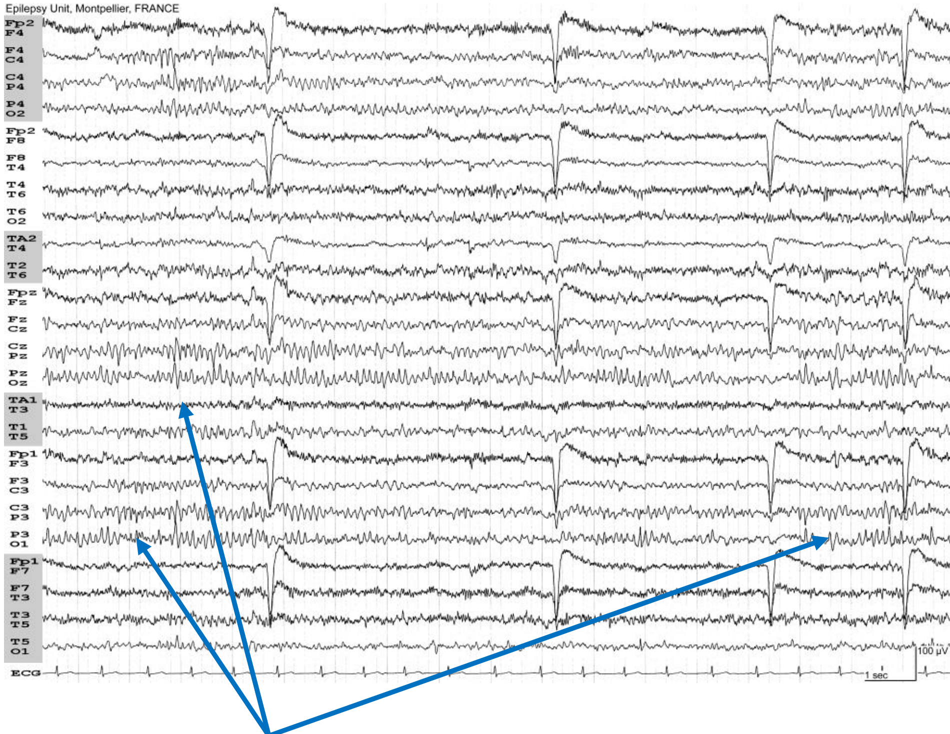

Midline theta rhythm
This rhythm may resemble to mu rhythm but it is not similarly reactive and occurs on the vertex region at 4-7 Hz.

Mu rhythm
Note the topographic distribution of the mu rhythm. To test the reactivity, it would have been necessary to have the patient move her right feet due to its localization near the vertex

Midline theta rhythm (Cigánek rhythm)

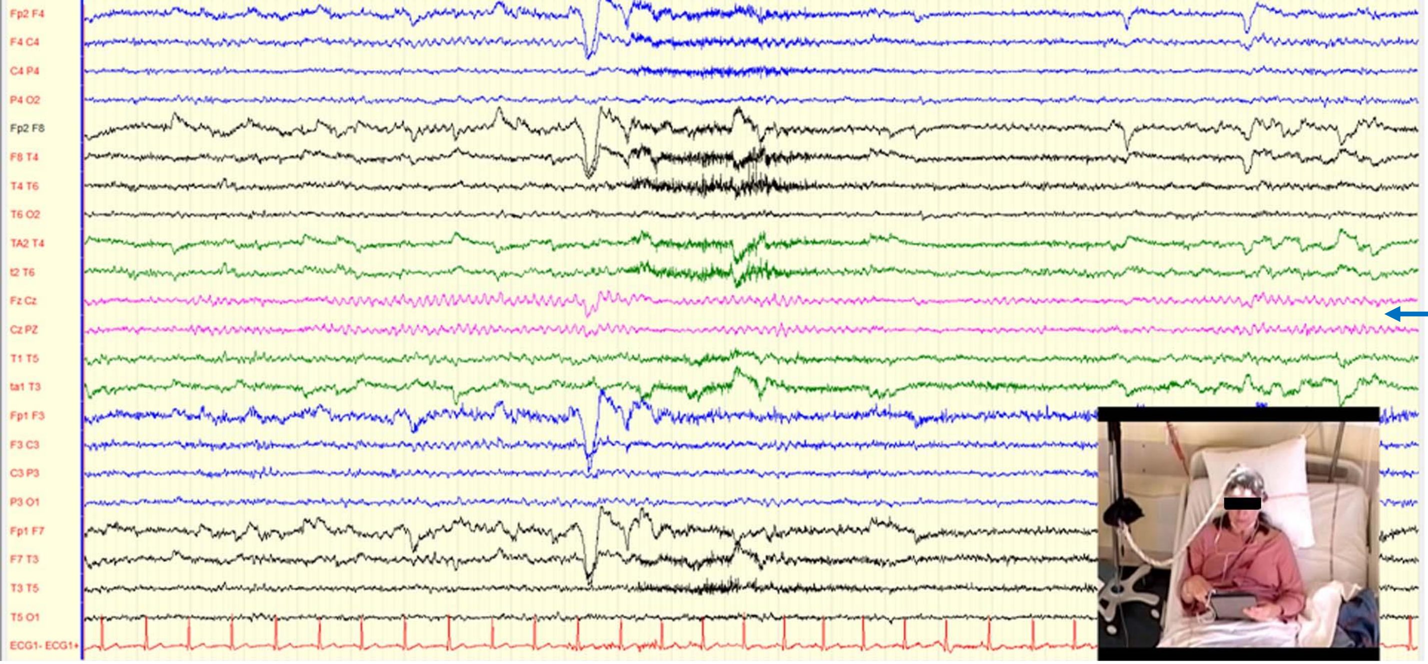

Midline theta rhythm while she was reading on her tablet.

15 mm/s
100 µV/cm

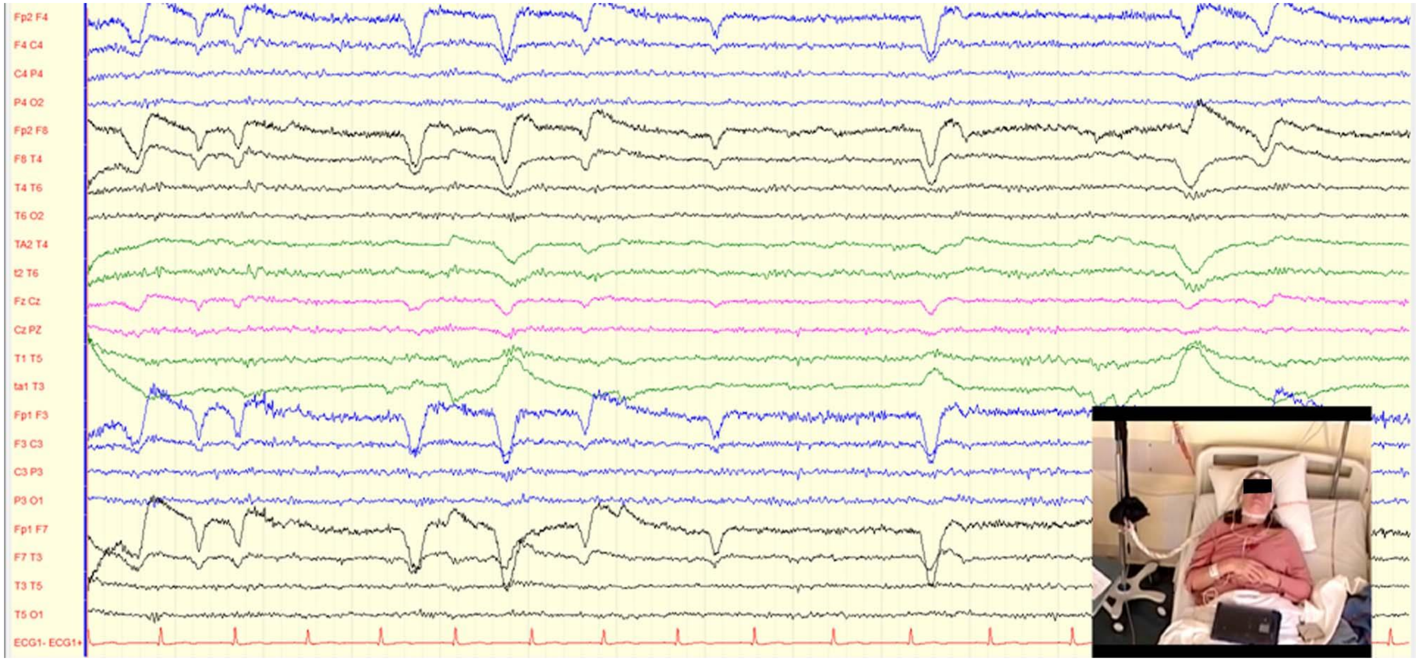

Eyes open.
No cognitive task.
She was looking up at the ceiling.

Lambda waves

- Lambda waves are observed during wakefulness when individuals are visually scanning a picture in a well-illuminated room.
- They are often associated with saccadic eye movements.
- They occur in the occipital regions.
- Lambda waves are often bilateral.
- Lambda waves disappear when the patient stares at a point and when the room is not as bright.

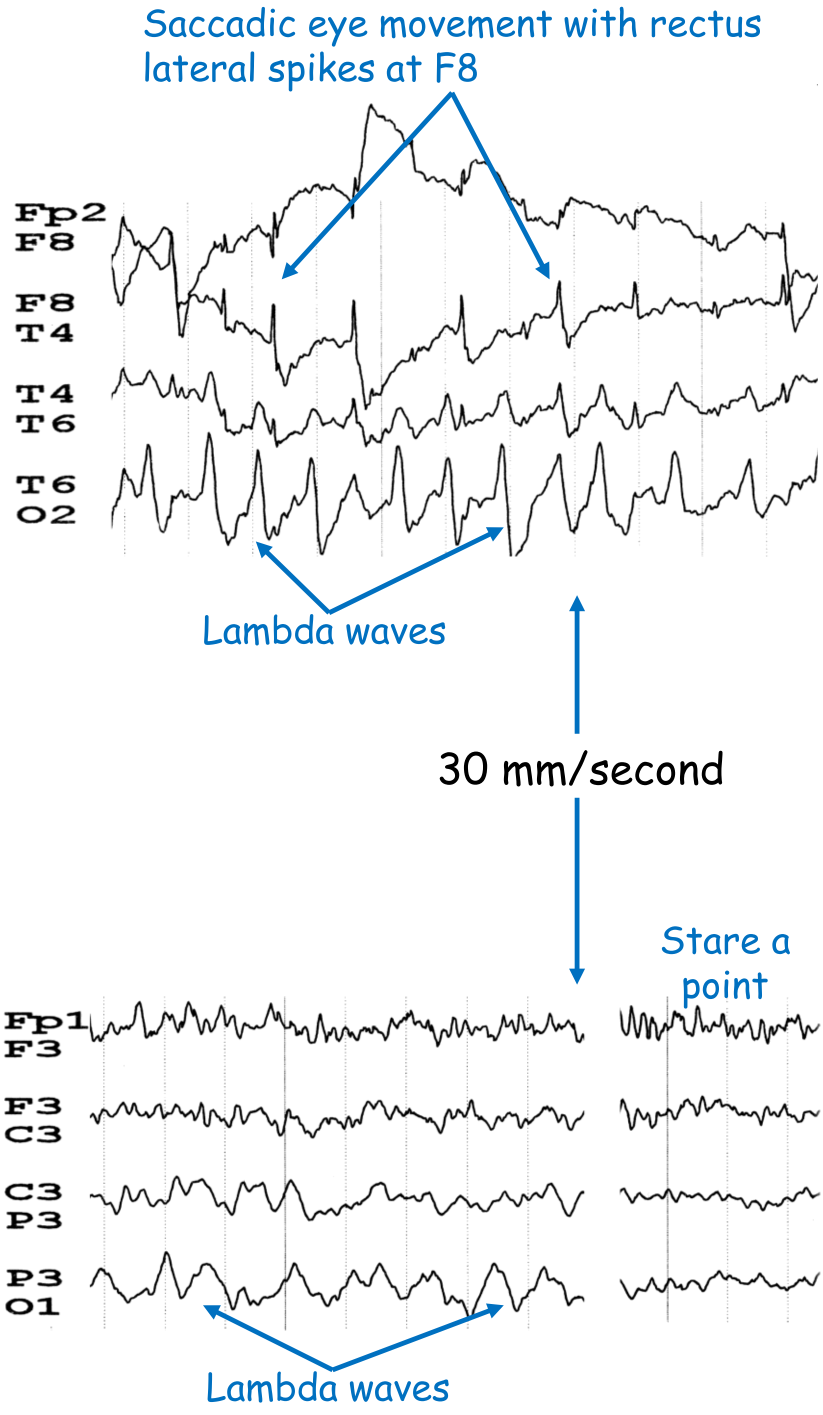

Eyes open.
She was reading a book

Lambda waves

40-year-old woman.
Left left lateral temporal lobe epilepsy

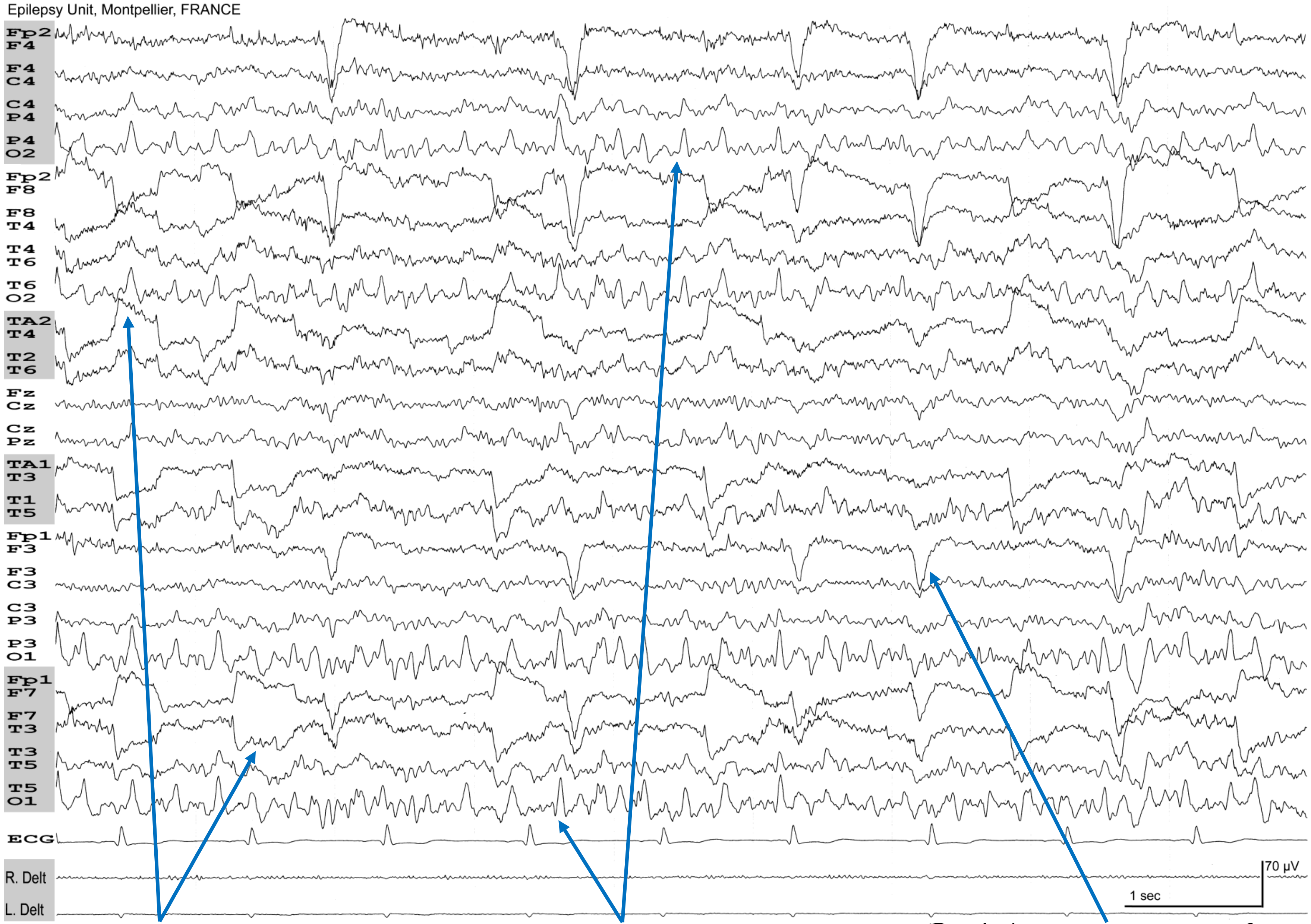

Saccadic eye movements Lambda waves Eyelid movement artifact

Same EEG panel at an EEG speed of 15 mm/s

Lambda waves

40-year-old woman.
Left lateral temporal lobe epilepsy

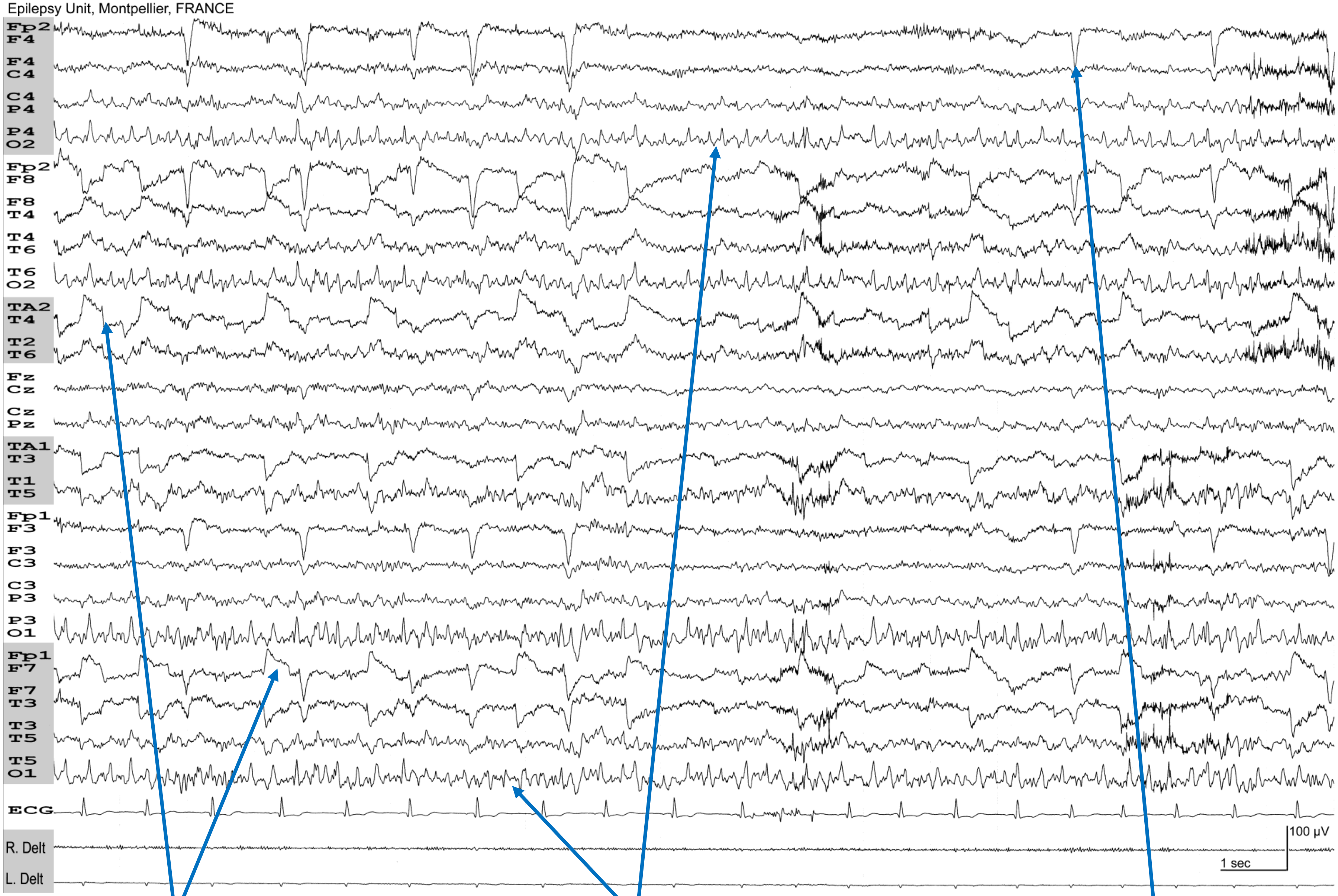

Saccadic eye movements

Lambda waves

Eyelid movement artifact

Same patient
Comparison when she was reading a book and reading on her tablet with a powerful activation of lambda waves and inversion of polarity.

Lambda waves

40-year-old woman.
Left lateral temporal lobe epilepsy

Epilepsy Unit, Montpellier, FRANCE

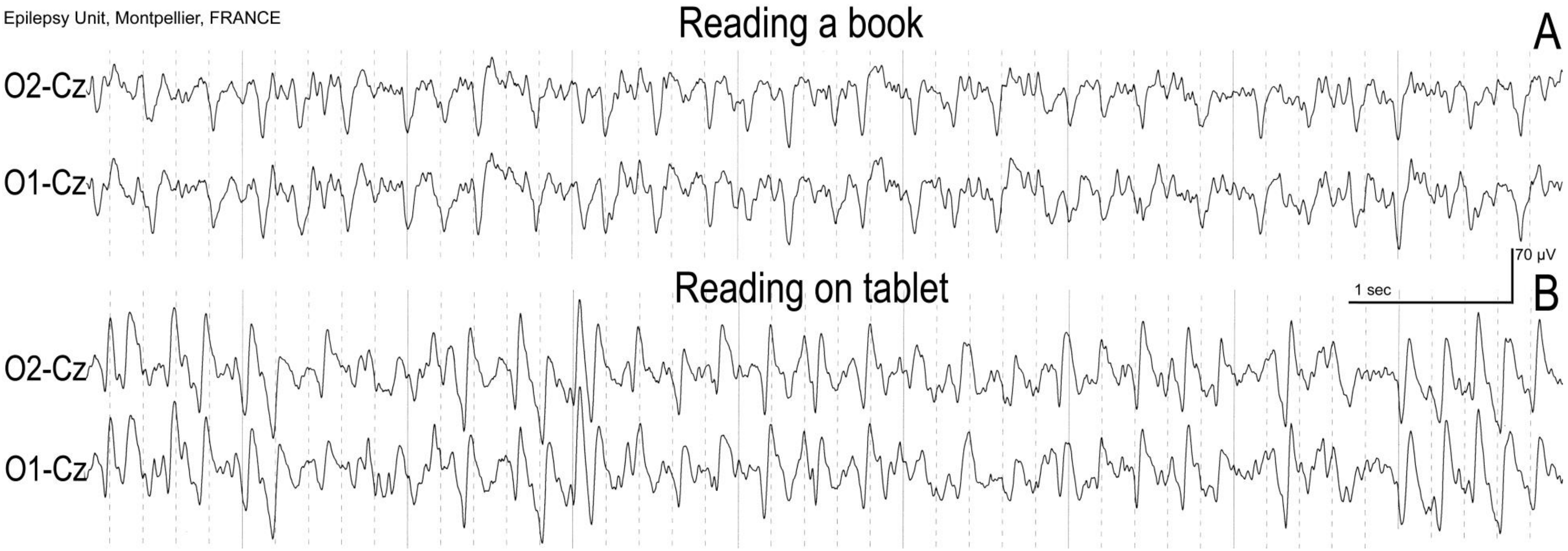

A. Patient reading a book. Typical lambda waves. The predominant phase is positive in occipital electrode O2 and O1.

B. Patient reading on tablet. Di- or triphasic lambda waves. The predominant phase is negative in occipital electrode O2 and O1.

Gélisse P, Crespel A. Powerful activation of lambda waves with inversion of polarity by reading on tablet. Epileptic Disord. 2024. doi: 10.1002/epd2.20197. OPEN ACCESS

Lambda waves

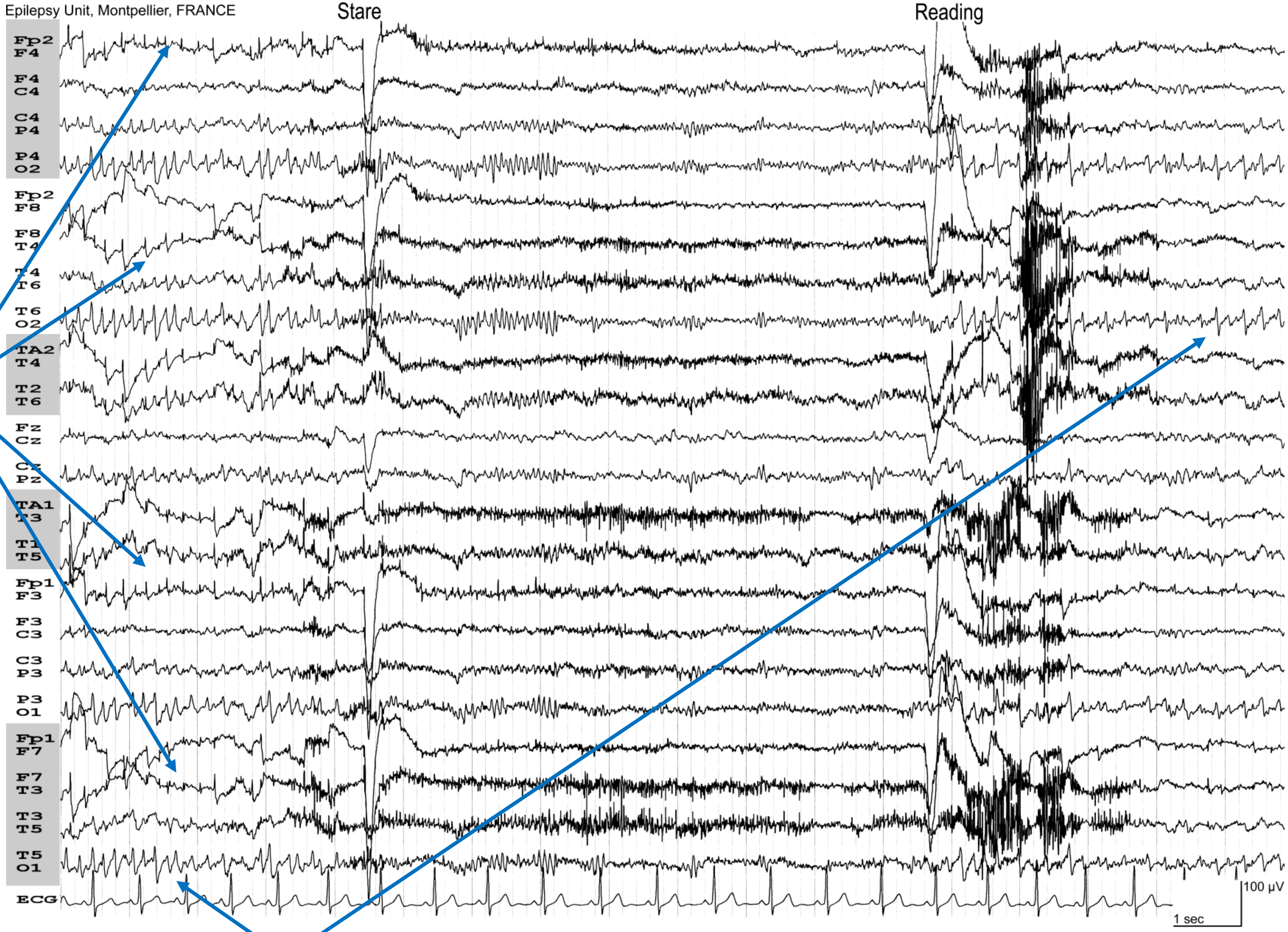

Rectus lateral spikes

24-year-old woman

Lambda waves

Lambda waves

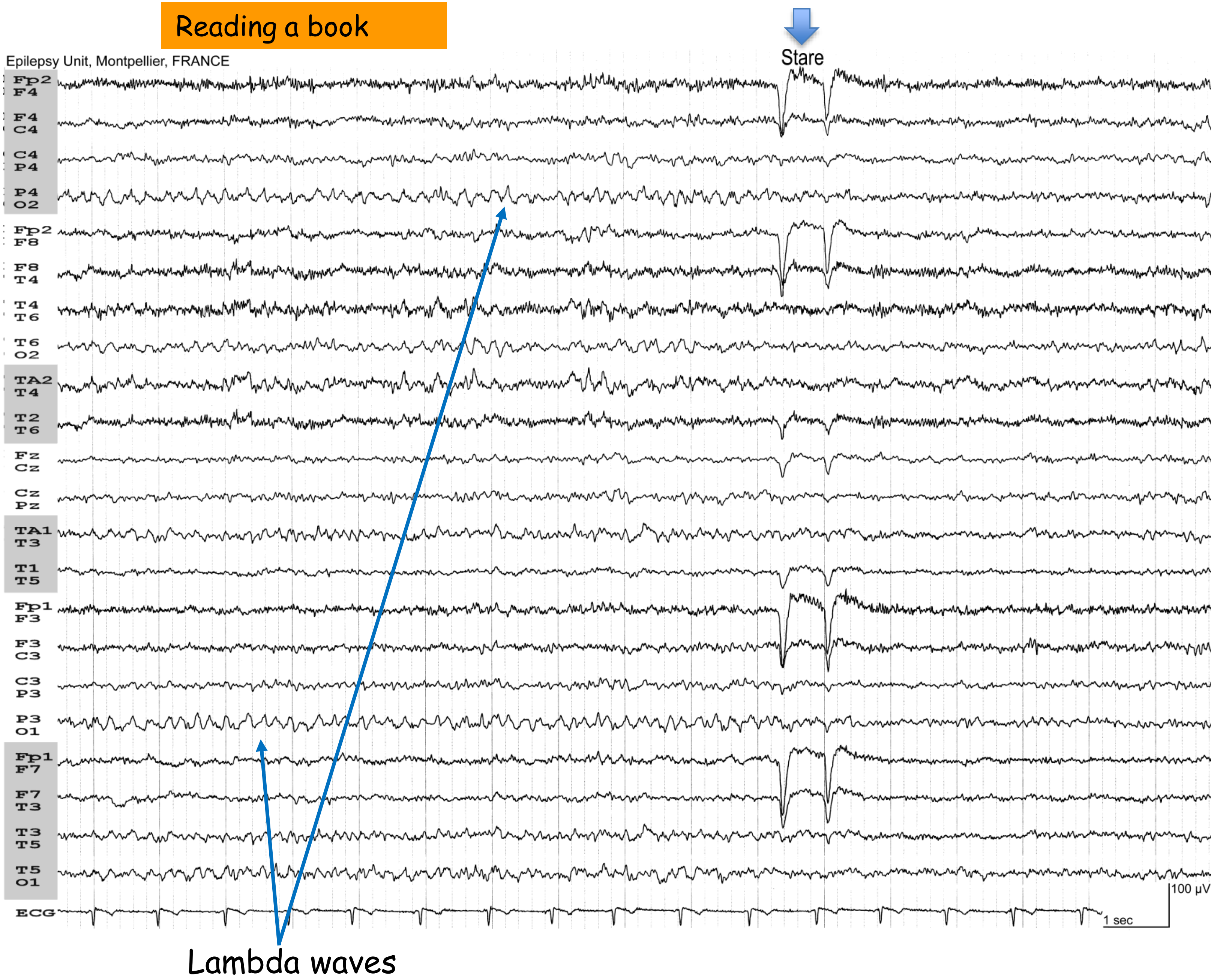

37-year-old woman

Positive occipital sharp transients of sleep (POSTs)

- POSTs occur, either symmetrically or not, on the occipital areas.
- POSTs are frequent in adolescents and young adults, and have no pathological significance.
- They are monophasic or diphasic
- POSTs generally occur in stage N2 and they may persist in stage N3.
- They disappear during REM sleep.

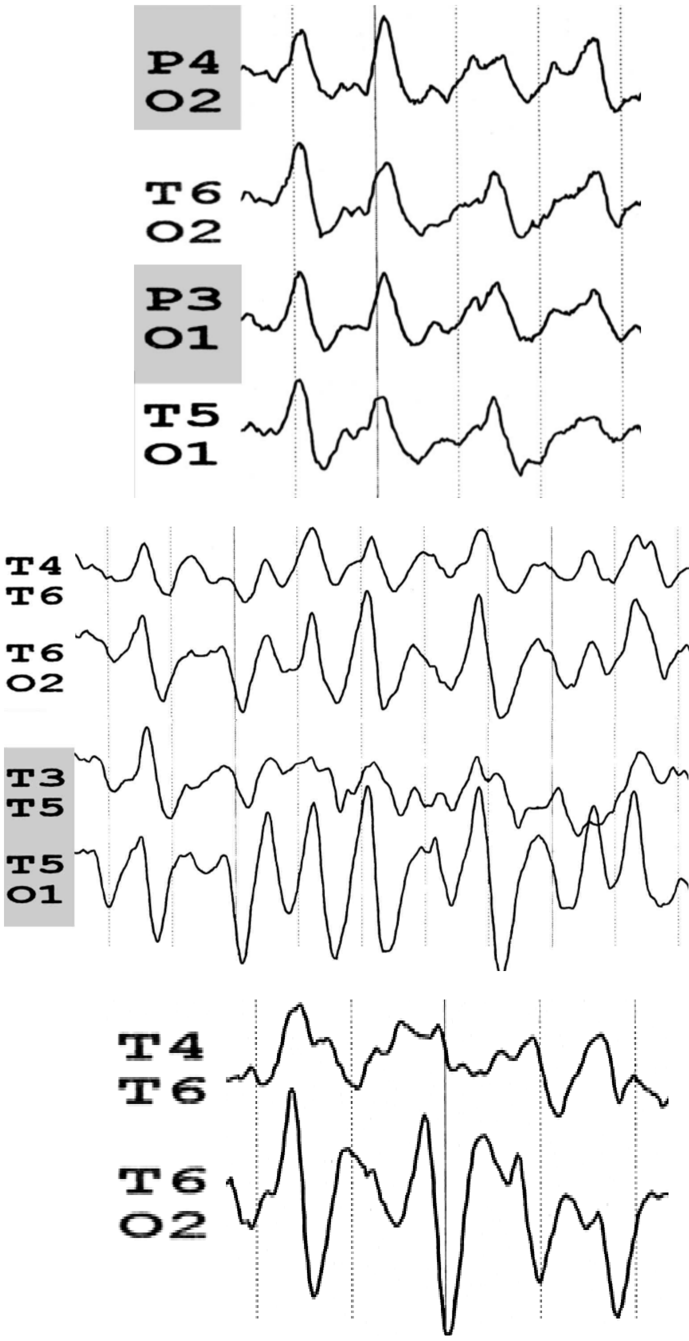

30 mm/second

Positive occipital sharp transients of sleep (POSTs) in NREM sleep stage 2

37-year-old woman with left temporal lobe epilepsy

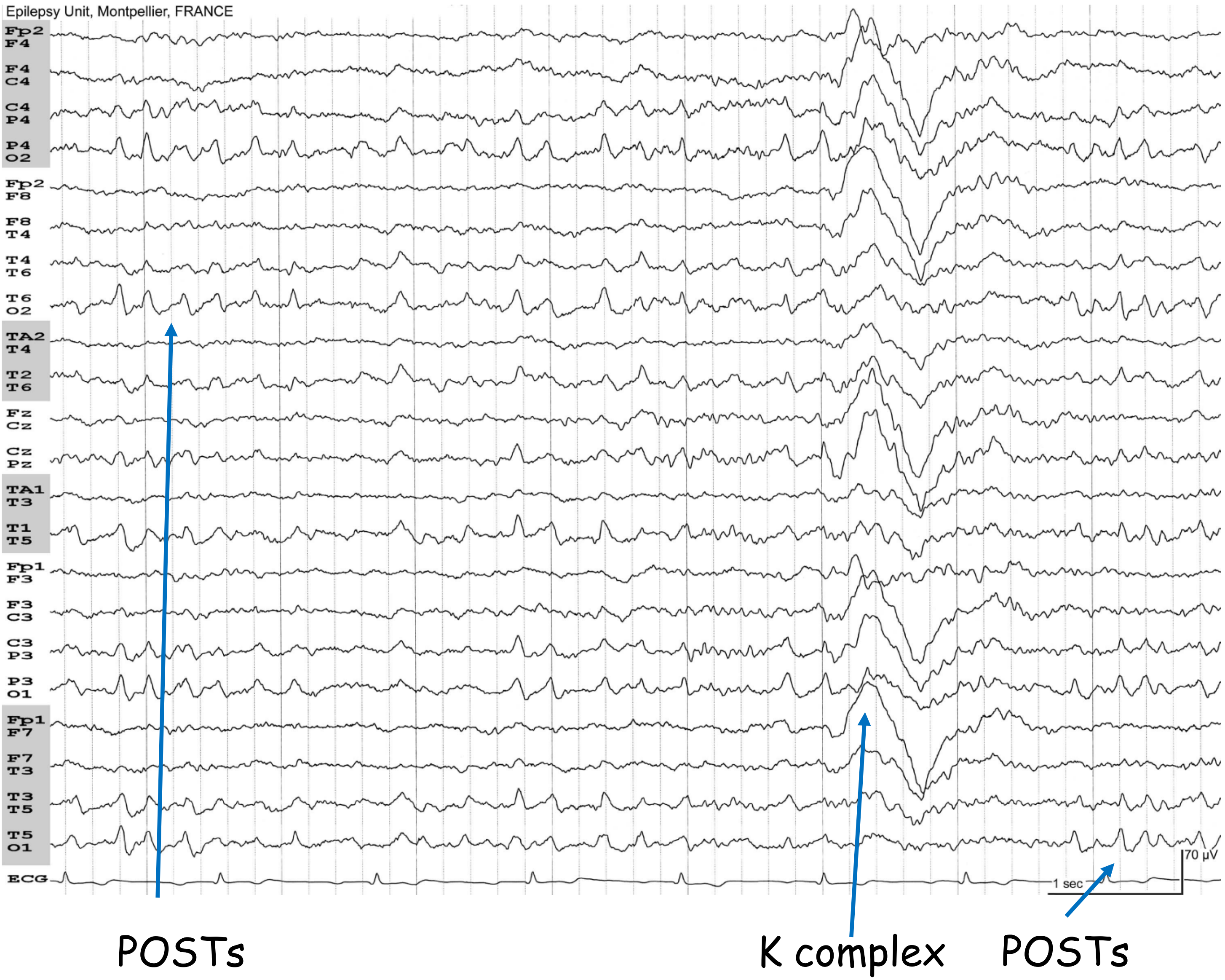

Positive occipital sharp transients of sleep (POSTs) in NREM sleep stage 2

19-year-old woman with juvenile myoclonic epilepsy

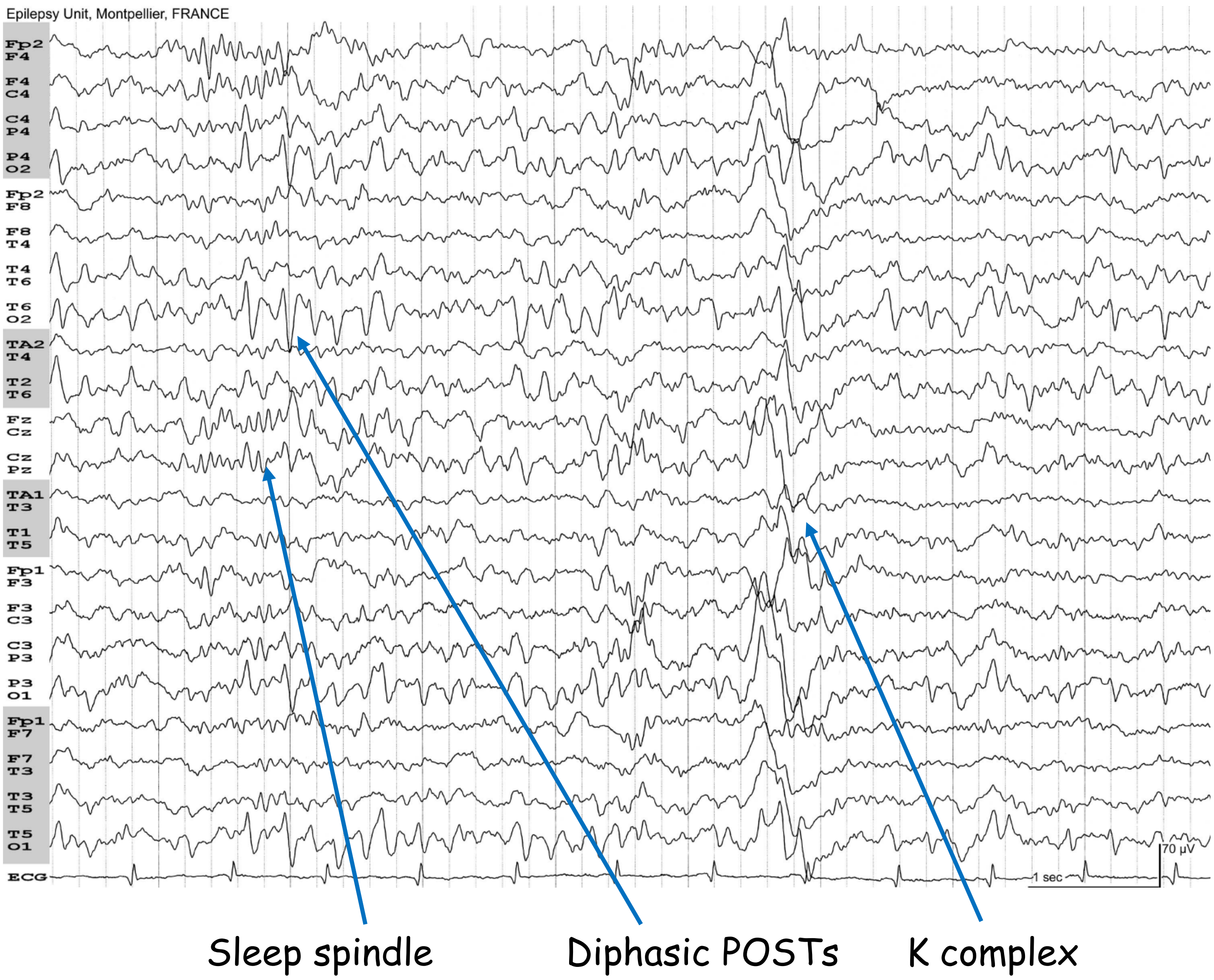

Positive occipital sharp transients of sleep (POSTs) in NREM sleep stage 2

27-year-old woman with cryptogenic left temporo-parieto-occipital junction epilepsy.

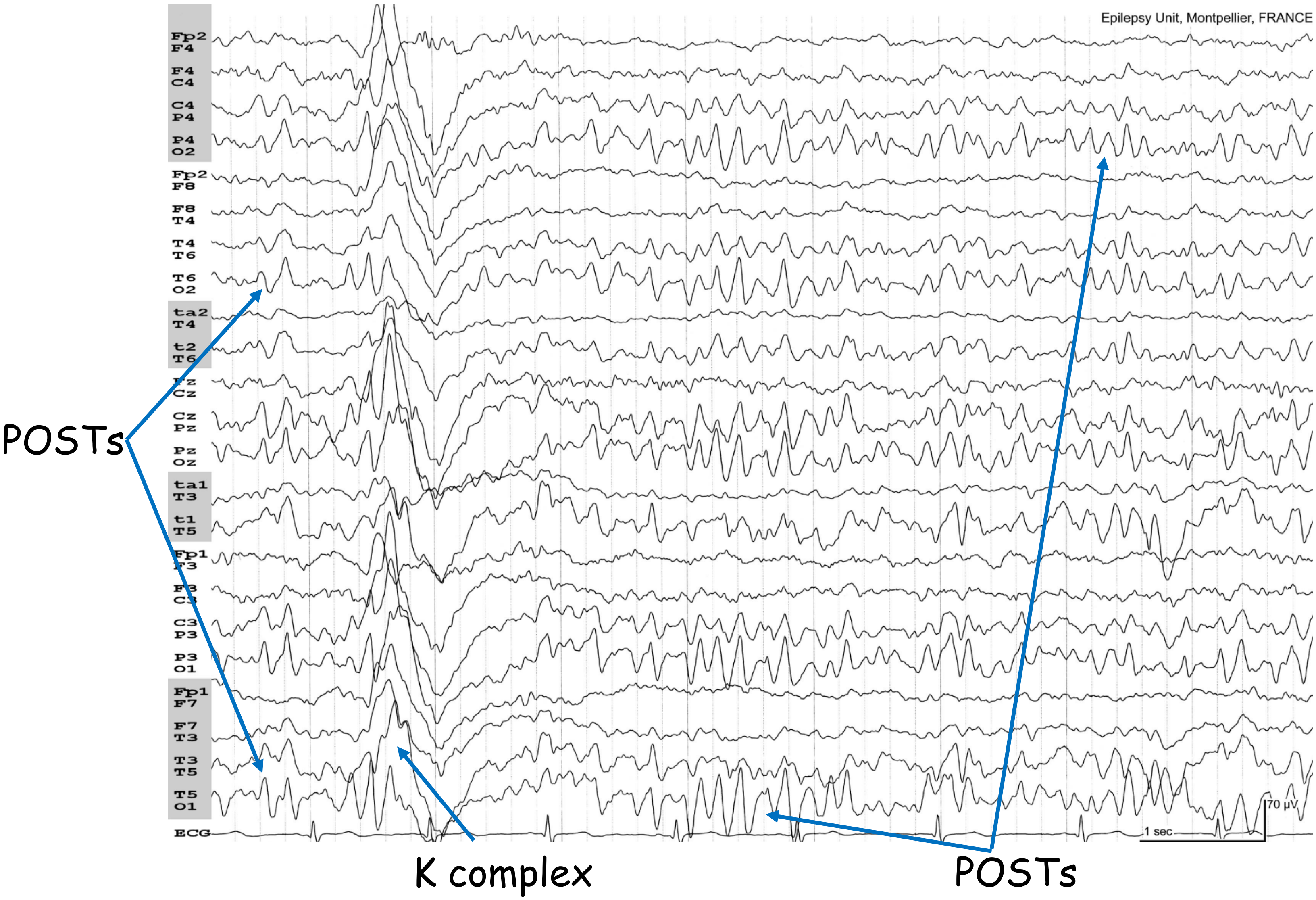

Positive occipital sharp transients of sleep (POSTs) in NREM sleep stage 2

Same EEG panel at an EEG speed of 15 mm/s

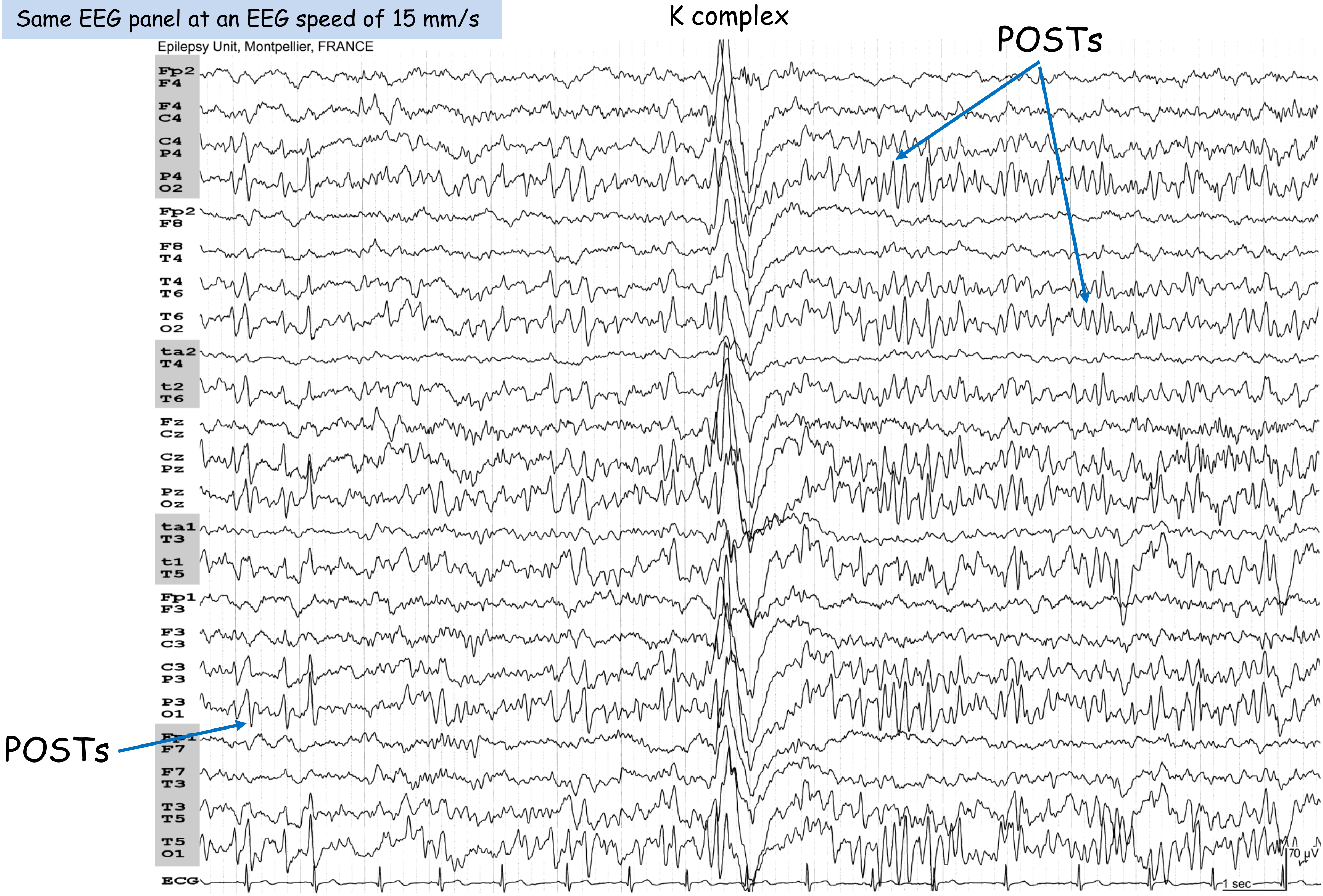

NREM 2 : A train of POSTs

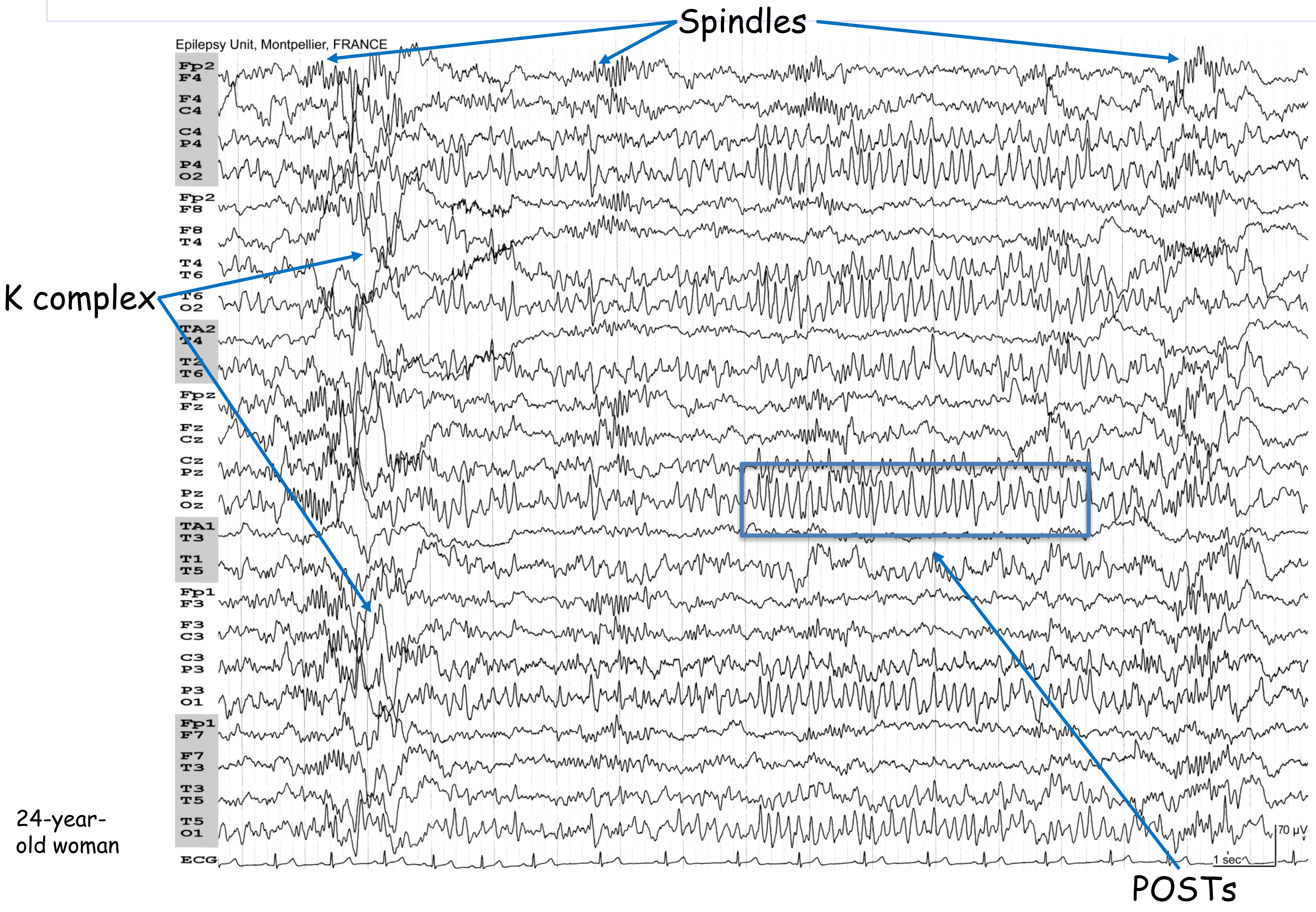

24-year-old woman

Wicket spikes

- Monophasic arciform waves.
- Occur over the temporal regions either bilaterally or independently.
- Occur generally during drowsiness and light sleep and disappear during deep sleep and reappear in REM sleep.

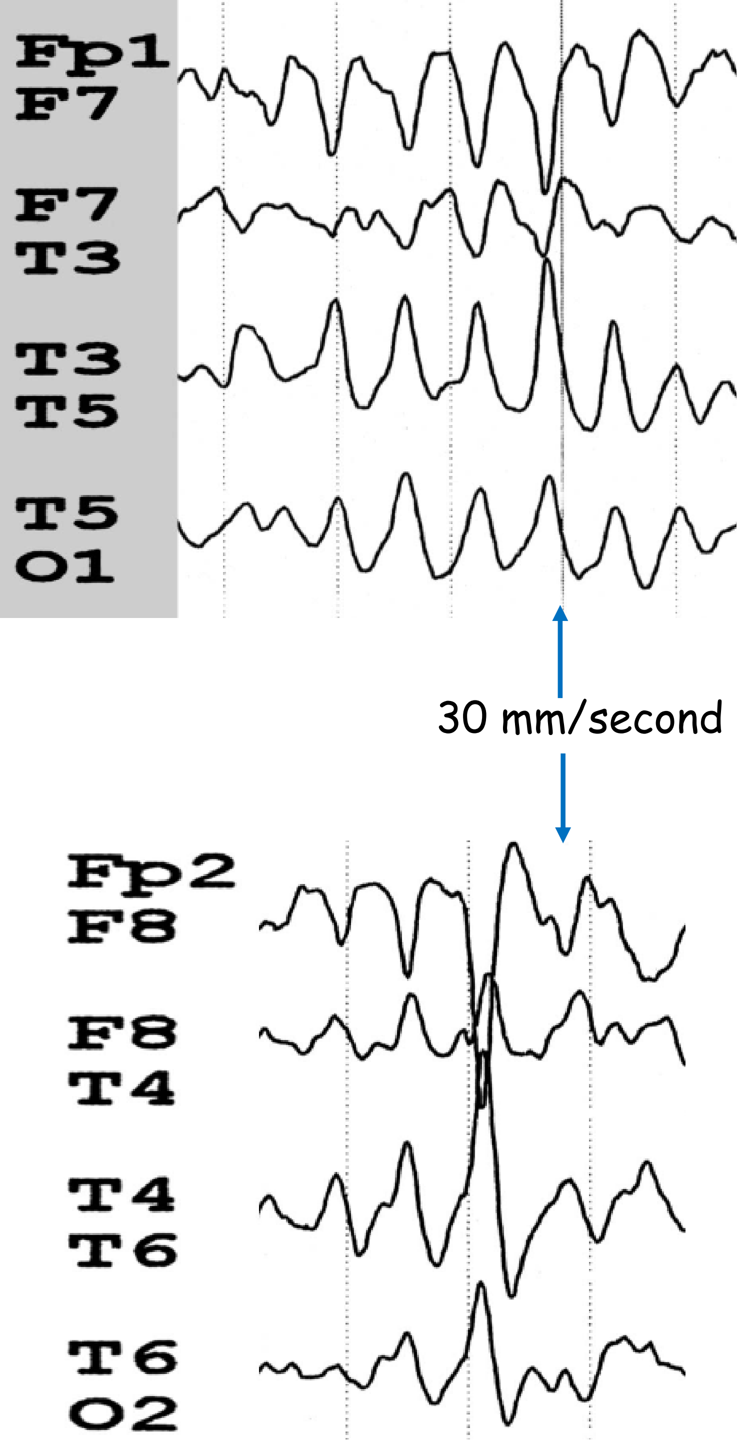

Wicket spikes

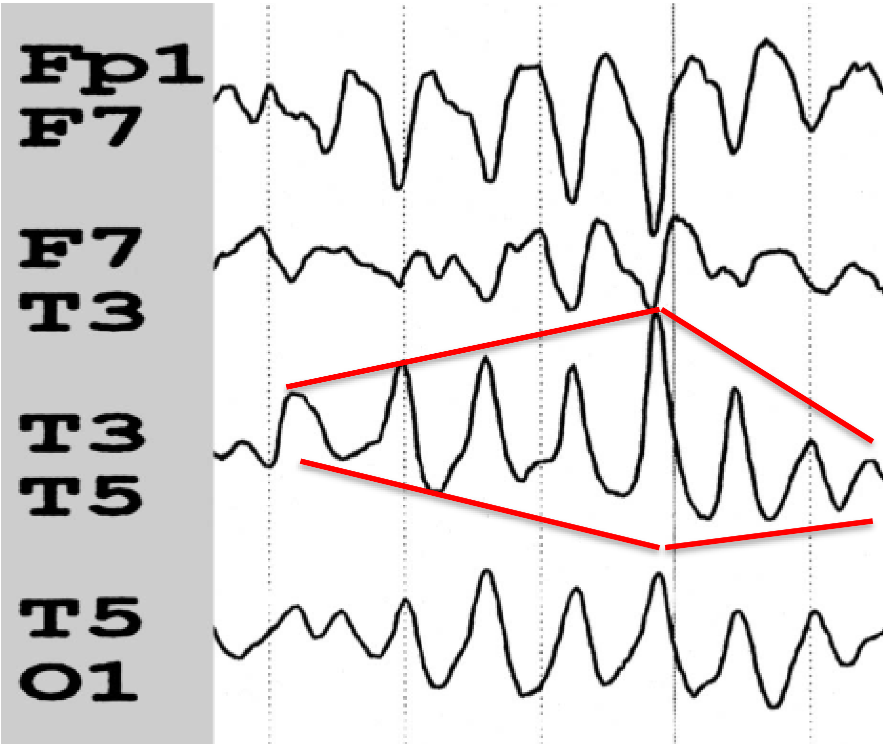

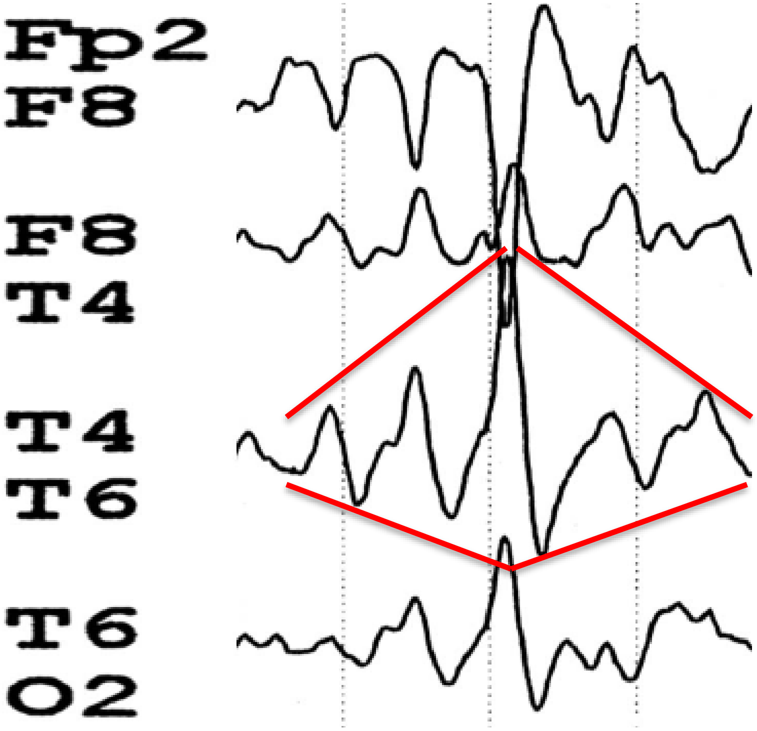

Typical morphology of diamond or lozenge-shaped

30 mm/second

Wicket spikes

WS appear most often in bursts, but occasionally singly, in which case they resemble an epileptiform spike activity.

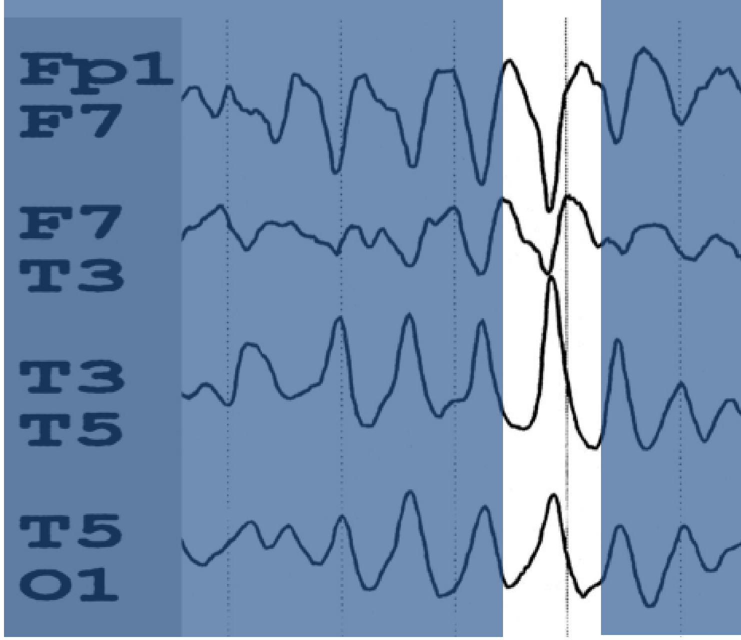

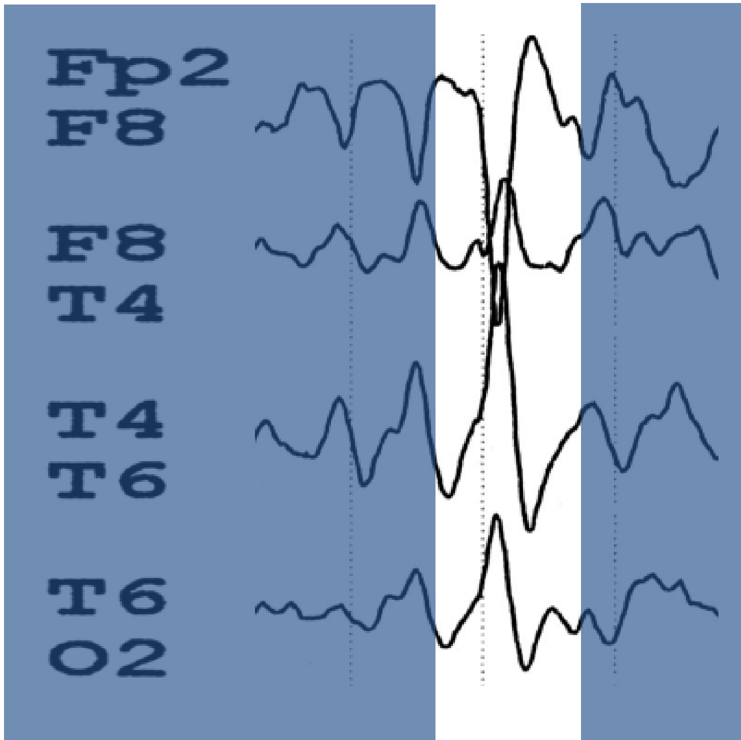

Wicket spikes

WS appear most often in bursts, but occasionally singly, in which case they resemble an epileptiform spike activity.

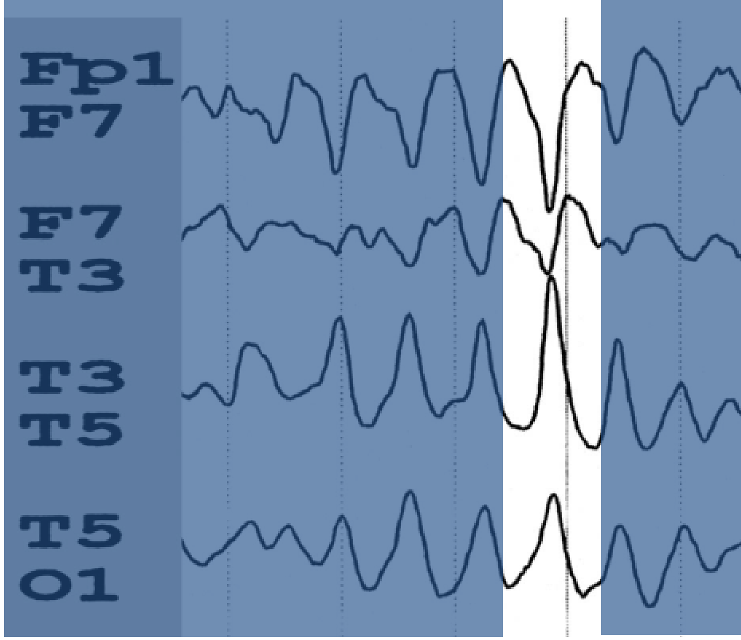

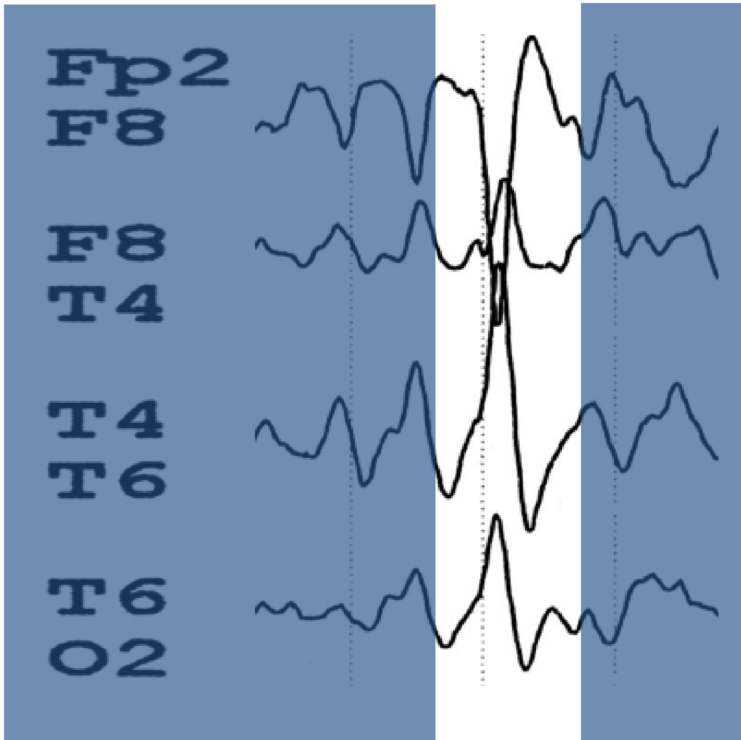

Second recommendation: If there is no wave after the spike, electroencephalographers should be suspicious of artifacts and normal EEG variants/variations of the background activity.

Wicket spikes

70-year-old woman with REM sleep behavioral disorder

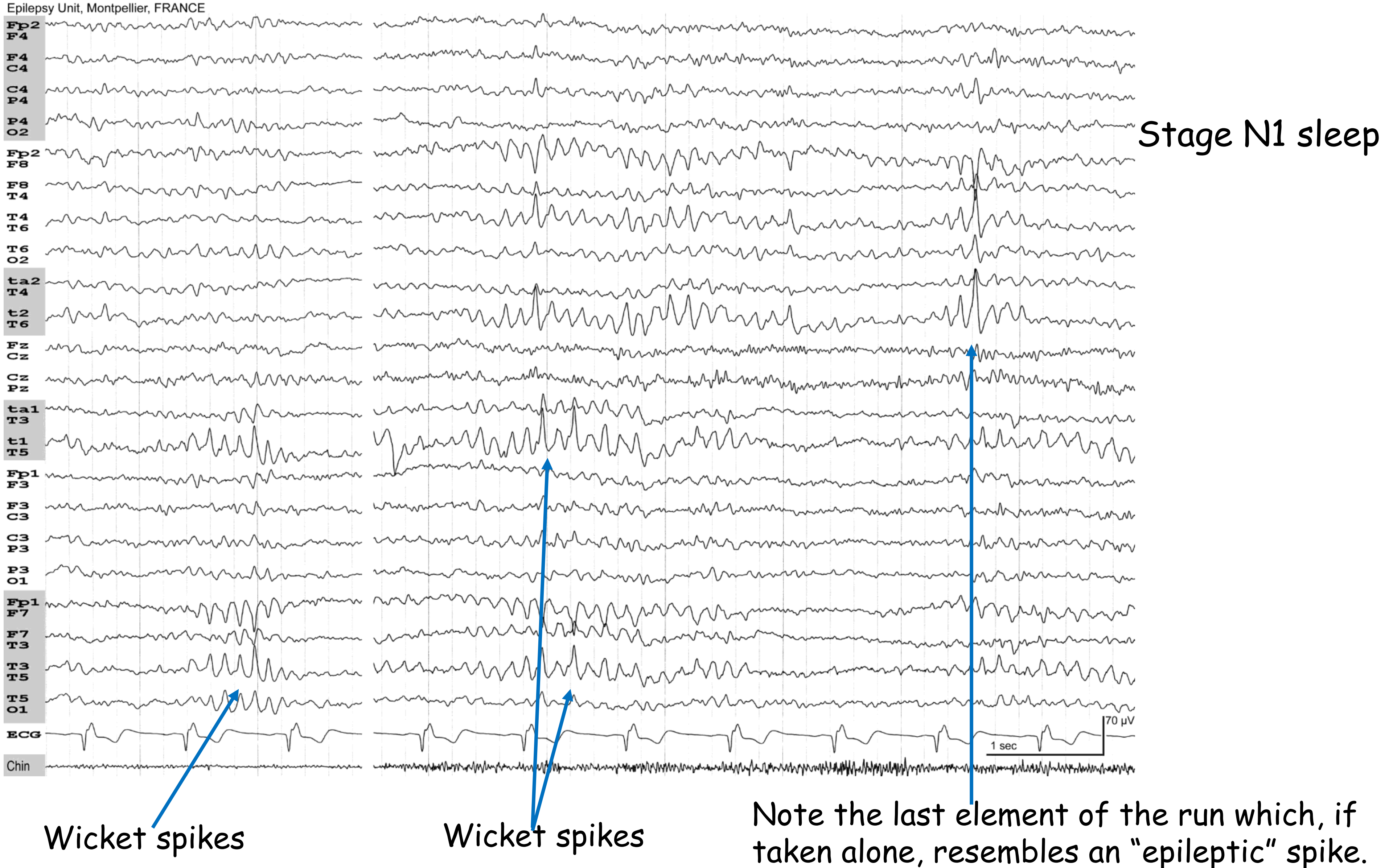

Wicket spikes

70-year-old woman with REM sleep behavioral disorder

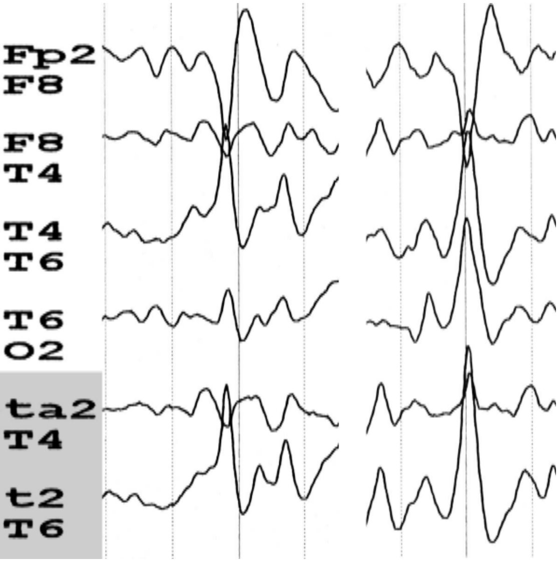

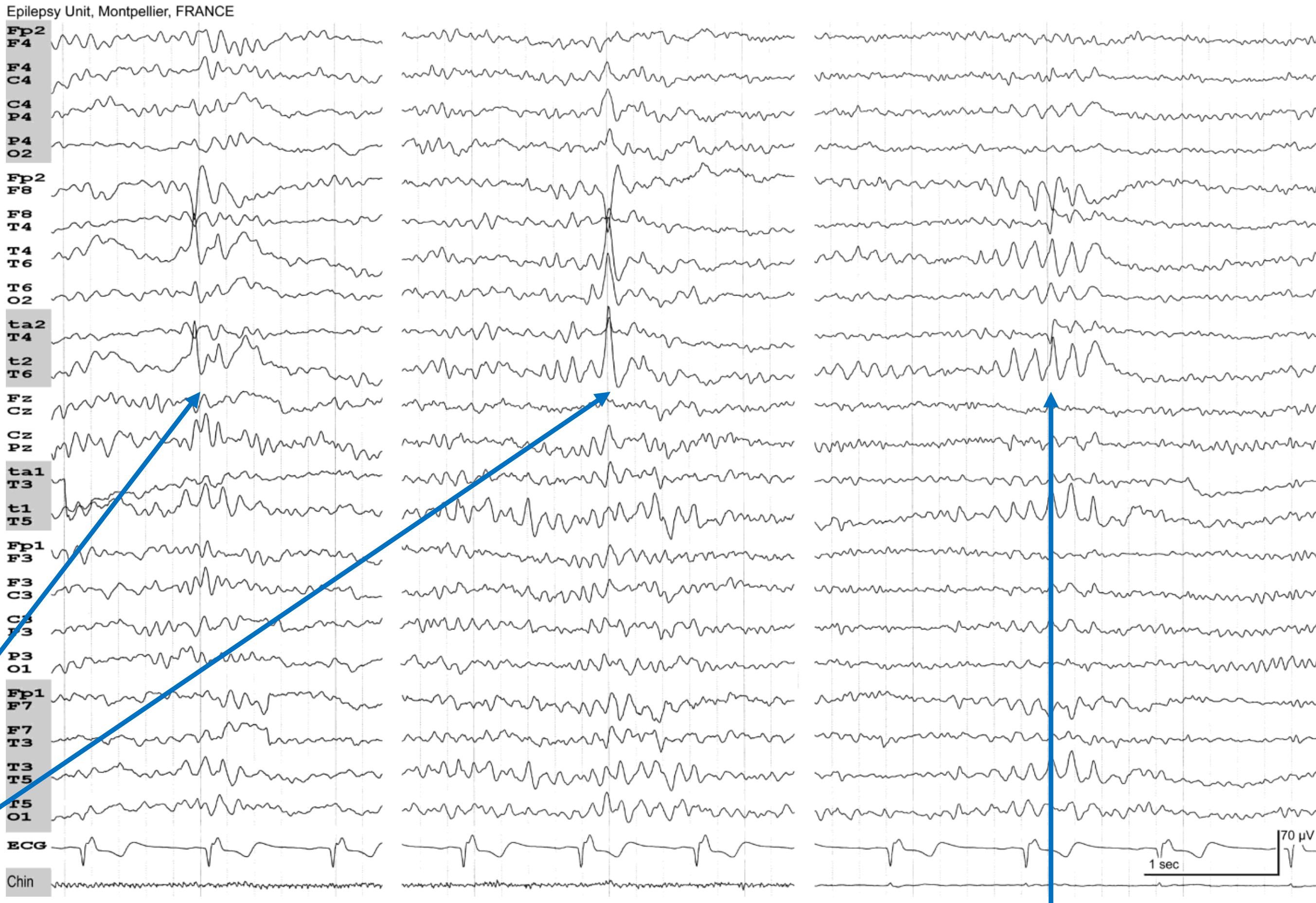

NREM sleep.
Isolated wicket spike which can be mistaken for an "epileptic" abnormality.

REM sleep. Wicket spikes over the right temporal region.

Wicket spikes

70-year-old man, left lateral temporal lobe epilepsy

Stage N2 sleep

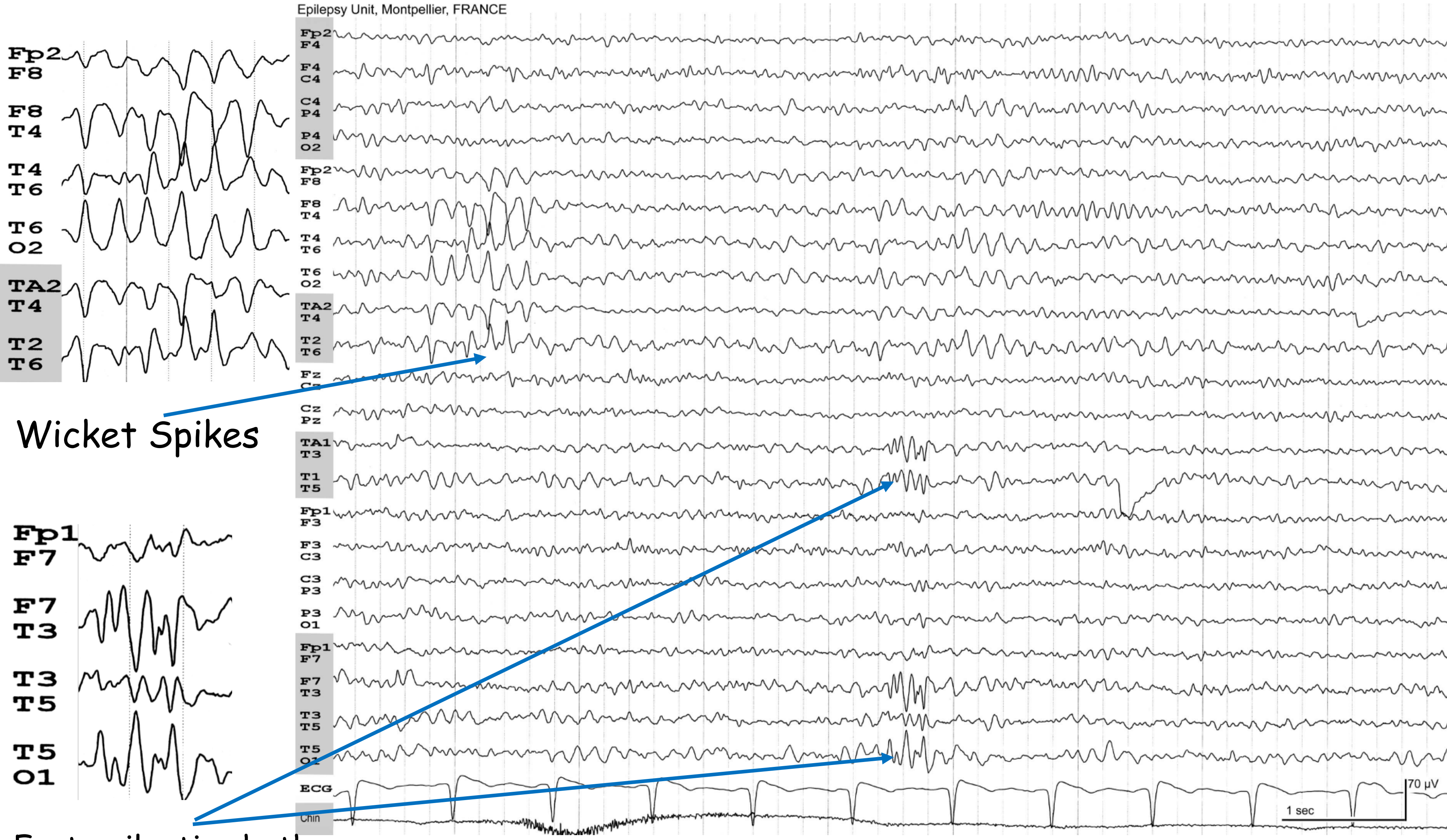

Fast epileptic rhythm

Wicket spikes

42-year-old woman with right temporal lobe epilepsy

Stage N2 Sleep

REM Sleep

Spindle

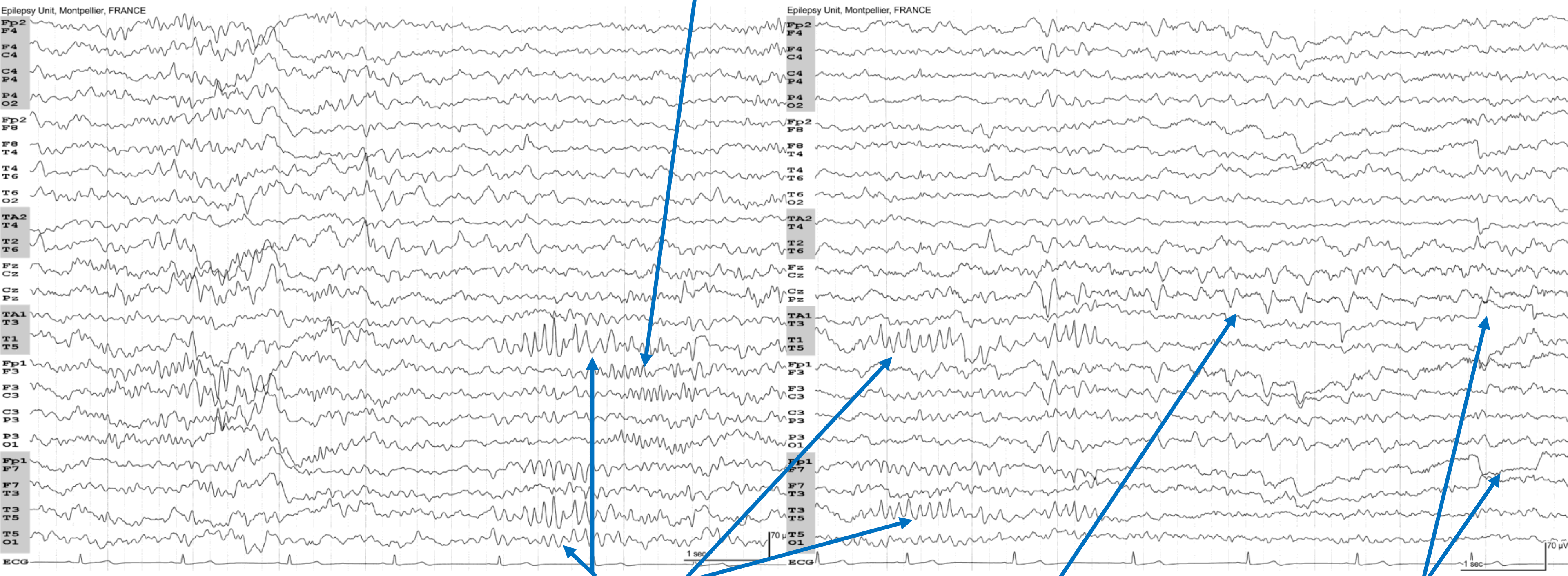

Wicket spikes over the left temporal region

Sawtooth waves

Rapid eye movements

Wicket spikes

42-year-old woman with right temporal lobe epilepsy

Stage N2 Sleep

REM Sleep

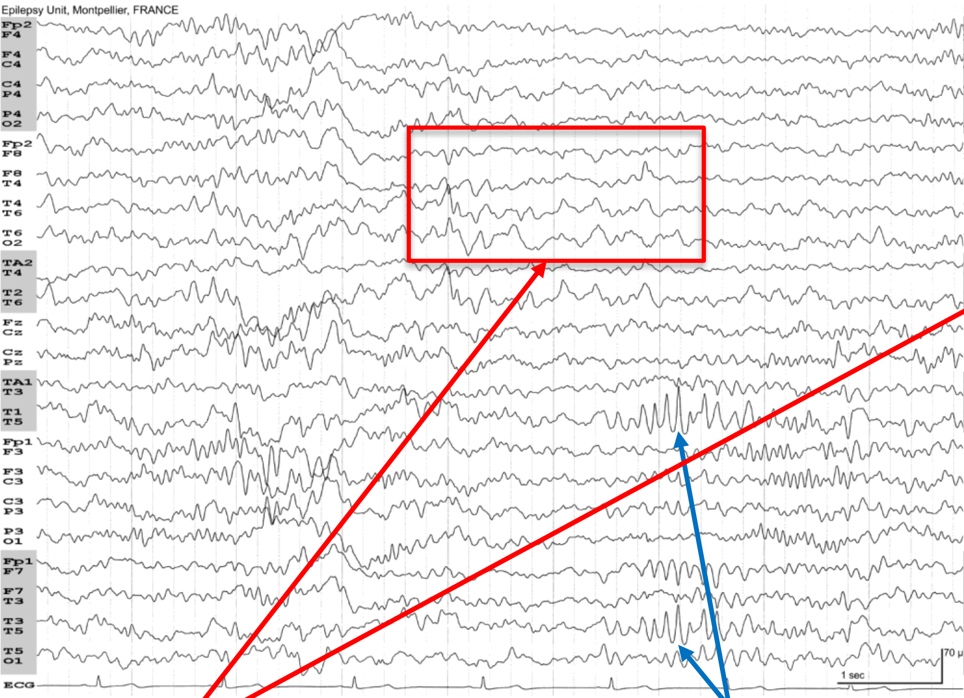

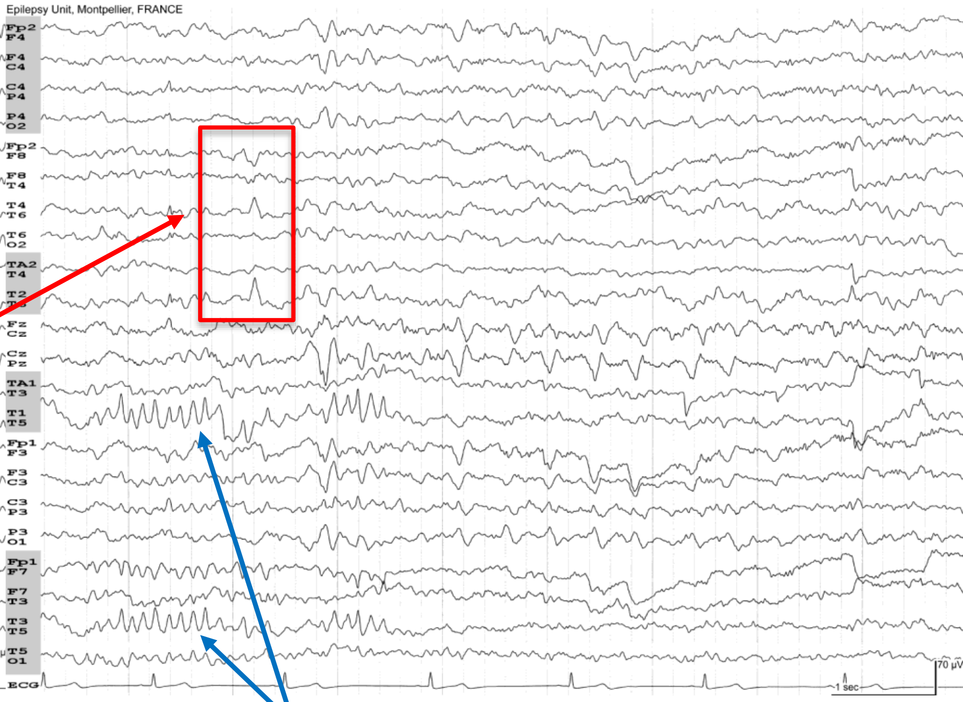

Interictal abnormalities

Wicket spikes over the left temporal region

Wicket spikes over the left temporal region

Wicket spikes

- 51-year-old woman
- Onset of seizure at age 30 with GTCS in the awakening period

NREM sleep

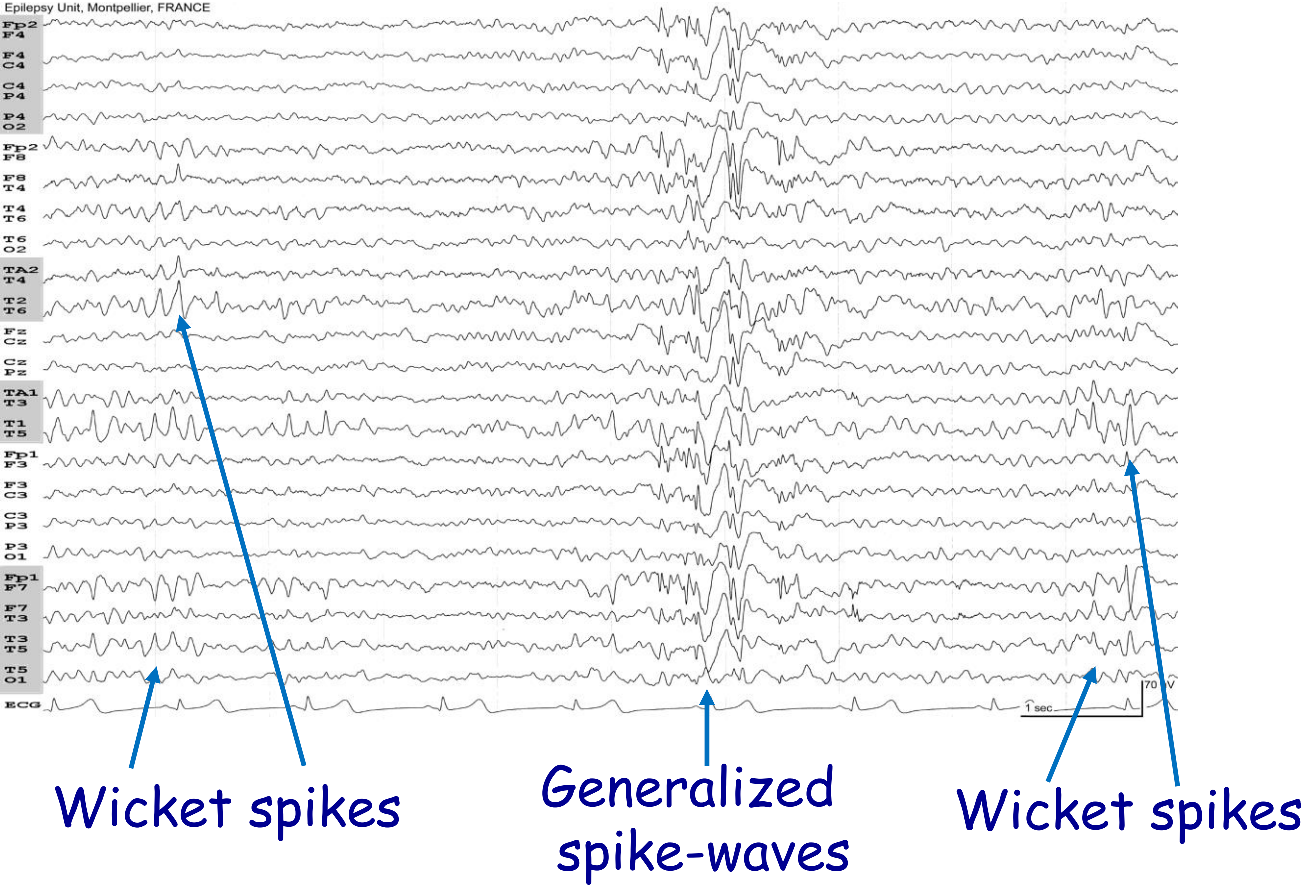

Idiopathic (genetic) generalized epilesy

Wicket spikes

49-year-old woman referred for episodes of loss of consciousness

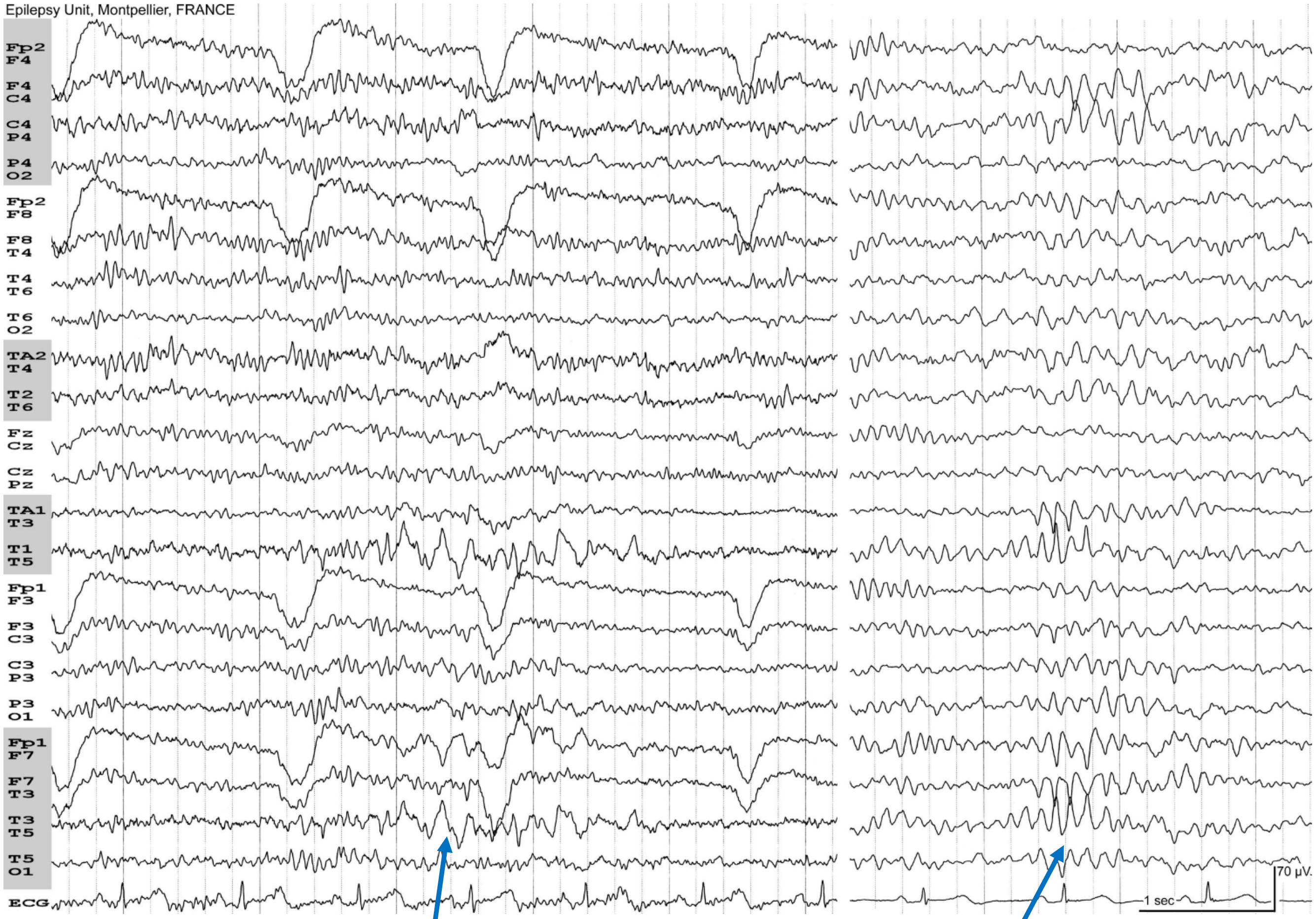

During wakefulness, the wicket spikes are often irregular with a theta-like morphology.

Typical wicket spikes appear during drowsiness and sleep.

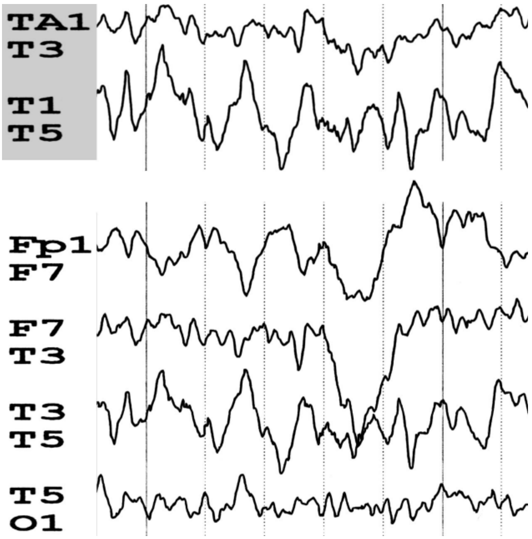

Awake

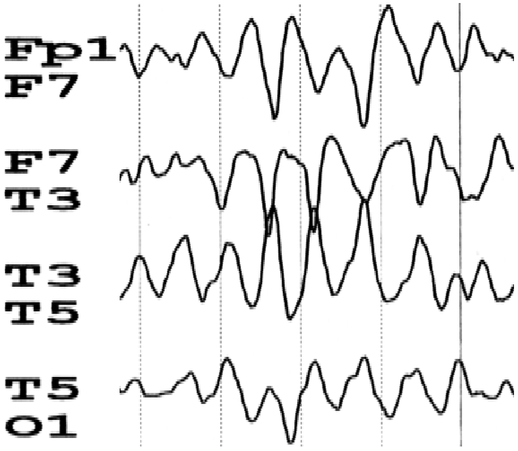

Stage N2 sleep

Wicket spikes

49-year-old woman referred for episodes of loss of consciousness

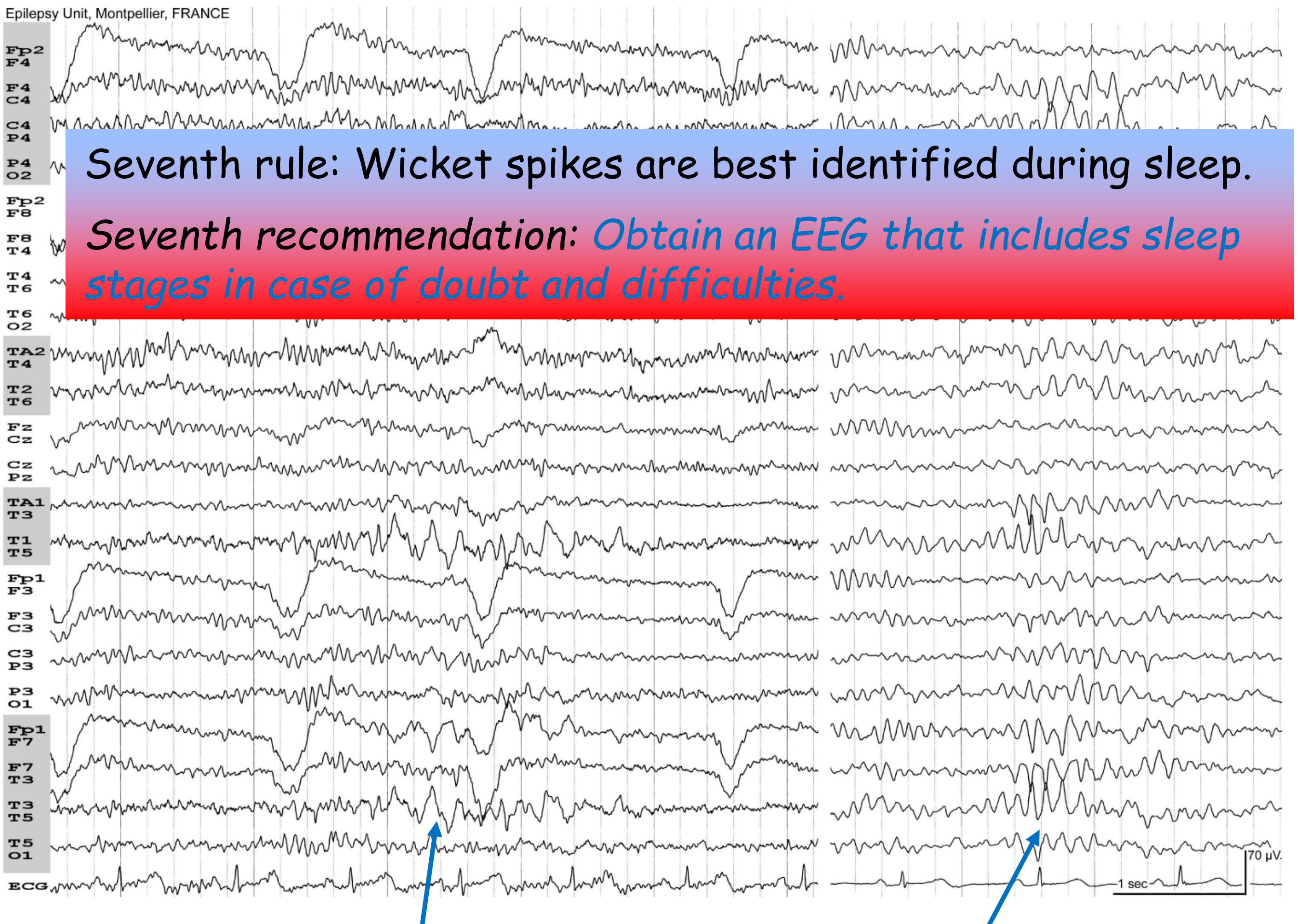

Seventh rule: Wicket spikes are best identified during sleep.

Seventh recommendation: Obtain an EEG that includes sleep stages in case of doubt and difficulties.

During wakefulness, the wicket spikes are often irregular with a theta-like morphology.

Typical wicket spikes appear during drowsiness and sleep.

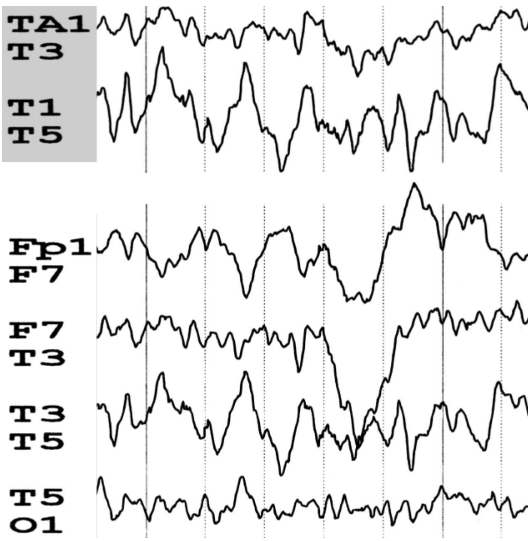

Awake

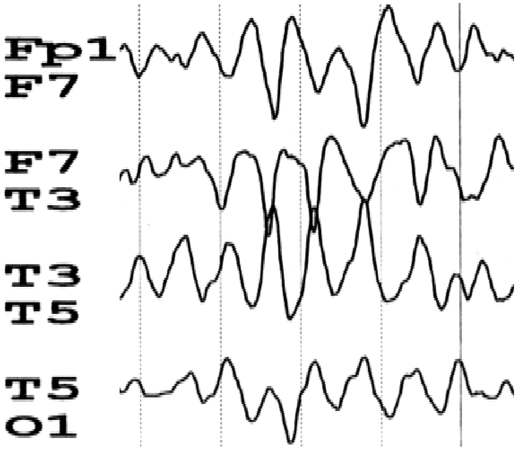

Stage N2 sleep

Rhythmic mid-temporal theta (burst) of drowsiness

- Lipman and Hughes (1969): rhythmic midtemporal discharge.
- Pattern: theta burst over the mid-temporal regions.
- Mostly seen during drowsiness and usually cease in deeper sleep but reappear in REM sleep.

Rhythmic mid-temporal theta (burst) of drowsiness

18-year-old man with syncope

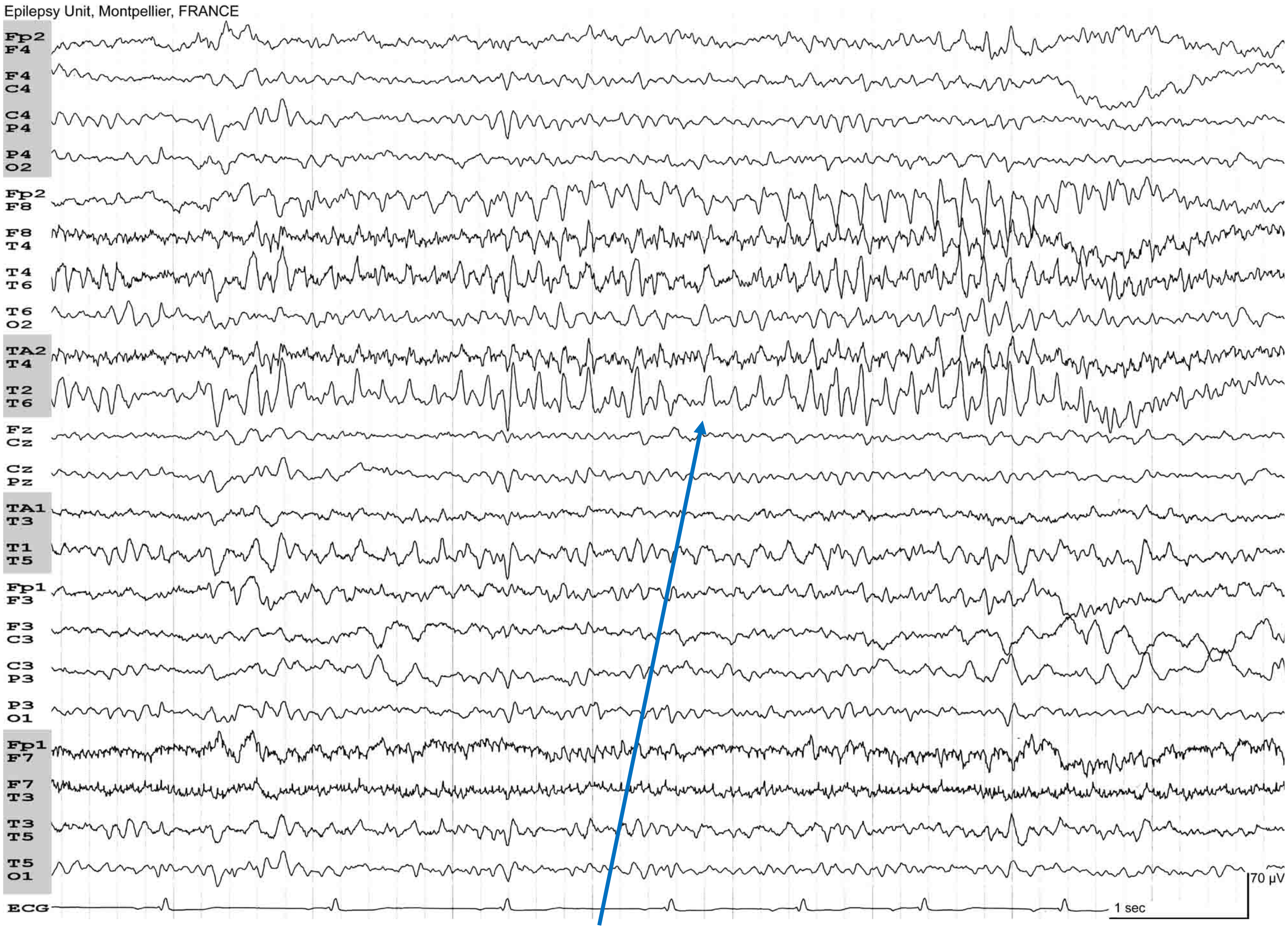

Rhythmic temporal theta burst of drowsiness

Rhythmic mid-temporal theta (burst) of drowsiness

16-year-old woman with Idiopathic (Genetic) Generalized Epilepsy

Drowsiness → Stage N1 Sleep

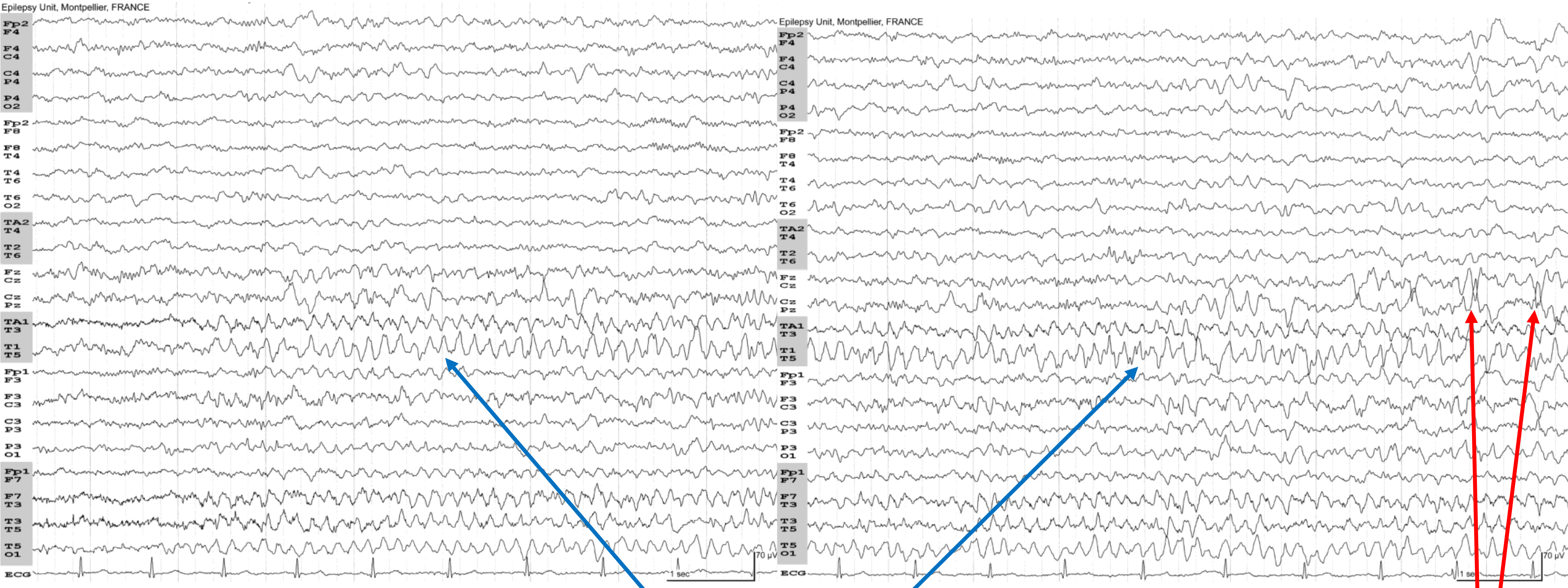

Rhythmic temporal theta burst of drowsiness

V waves

Rhythmic mid-temporal theta (burst) of drowsiness

16-year-old woman with Idiopathic (Genetic) Generalized Epilepsy

Drowsiness → Stage N1 Sleep

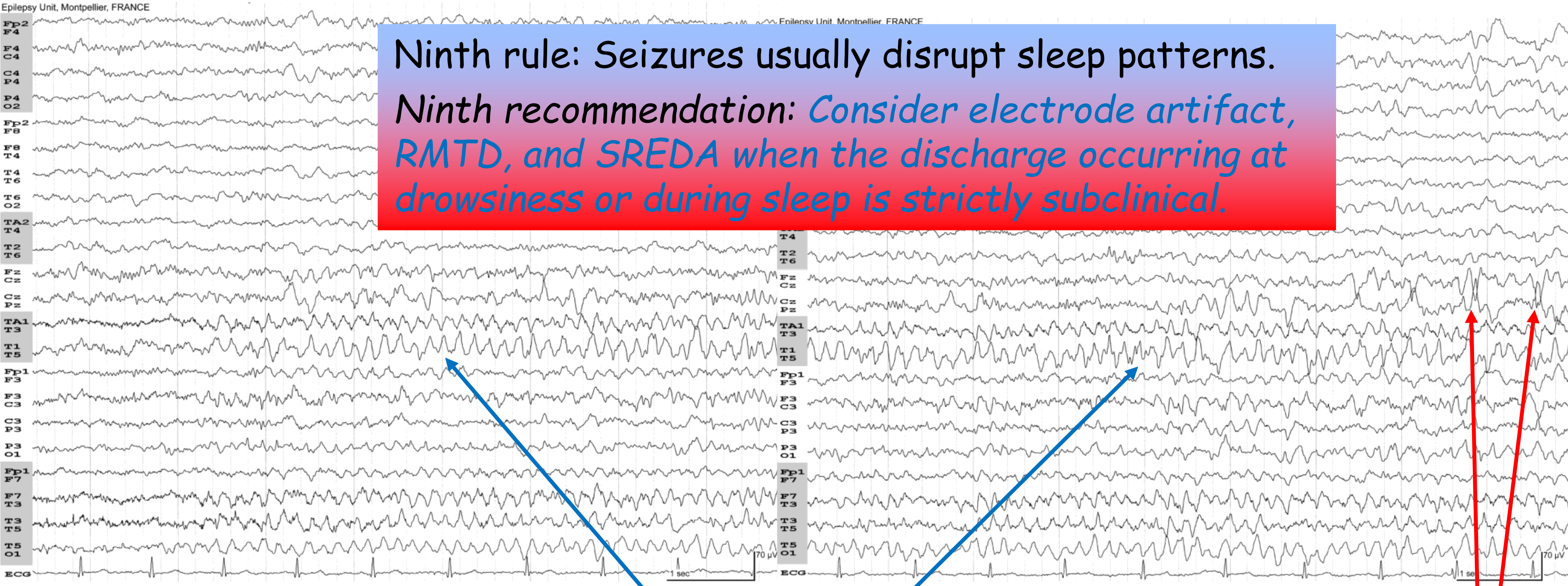

Ninth rule: Seizures usually disrupt sleep patterns.

Ninth recommendation: Consider electrode artifact, RMTD, and SREDA when the discharge occurring at drowsiness or during sleep is strictly subclinical.

Rhythmic temporal theta burst of drowsiness

V waves

Rhythmic mid-temporal theta (burst) of drowsiness

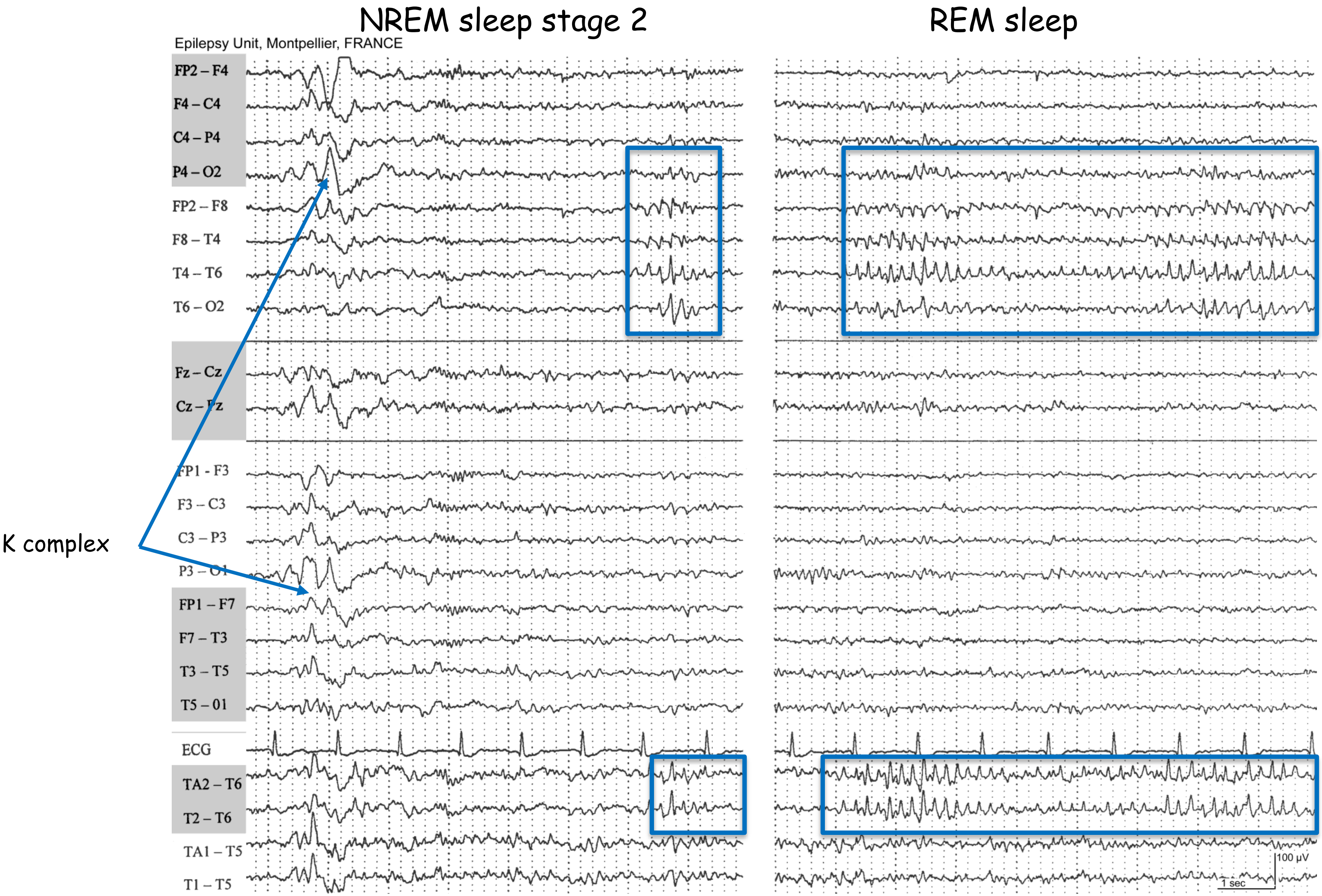

Rhythmic mid-temporal theta (burst) of drowsiness and mu rhythm in REM sleep

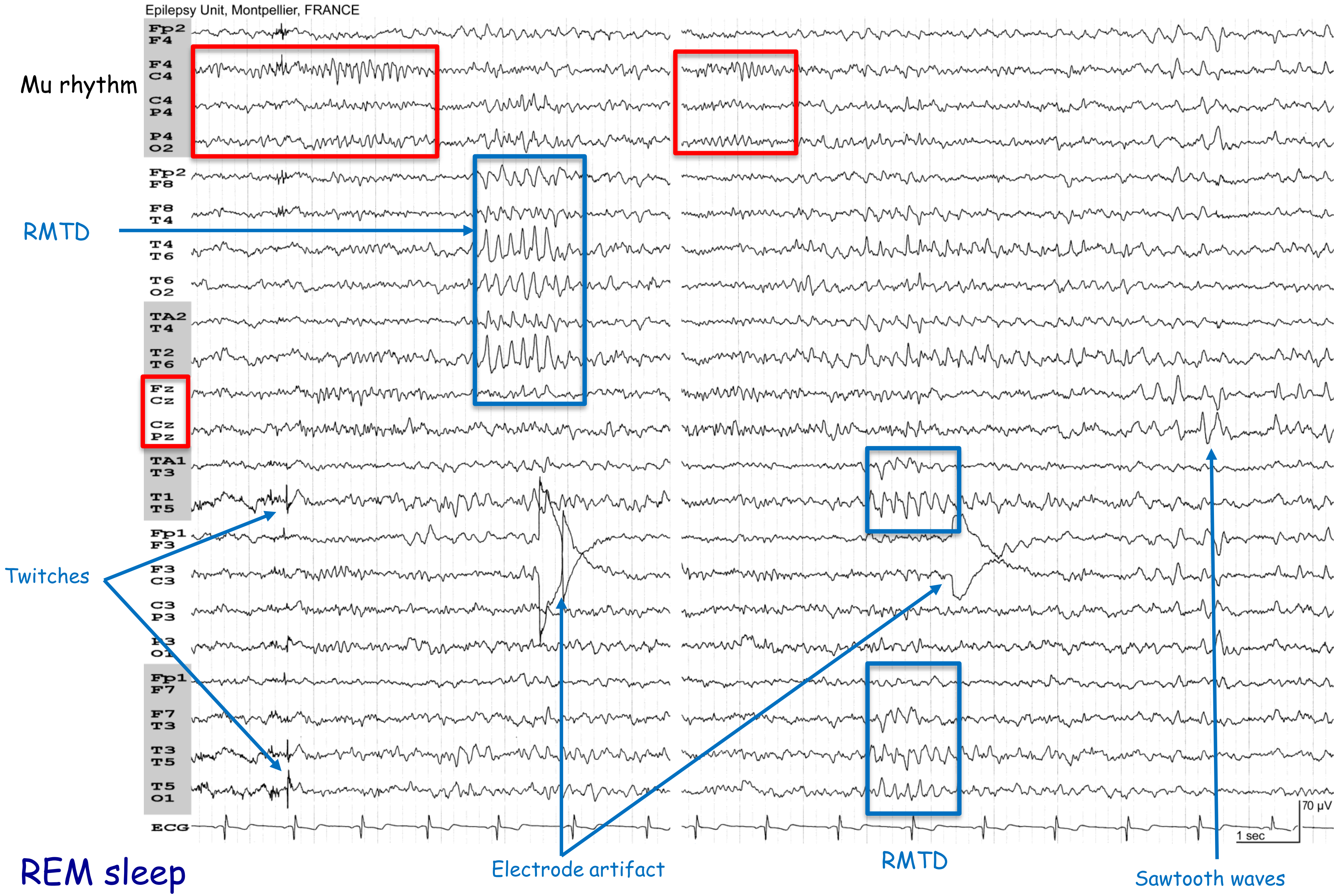

Small Sharp Spikes

- Low-voltage diphasic spikes which occur singly over the anterior or mid-temporal regions.
- SSS generally occur as a unilateral waveform, but can occur bisynchronously or independently.
- They are mainly seen during drowsiness and at the onset of light sleep.

72-year-old woman

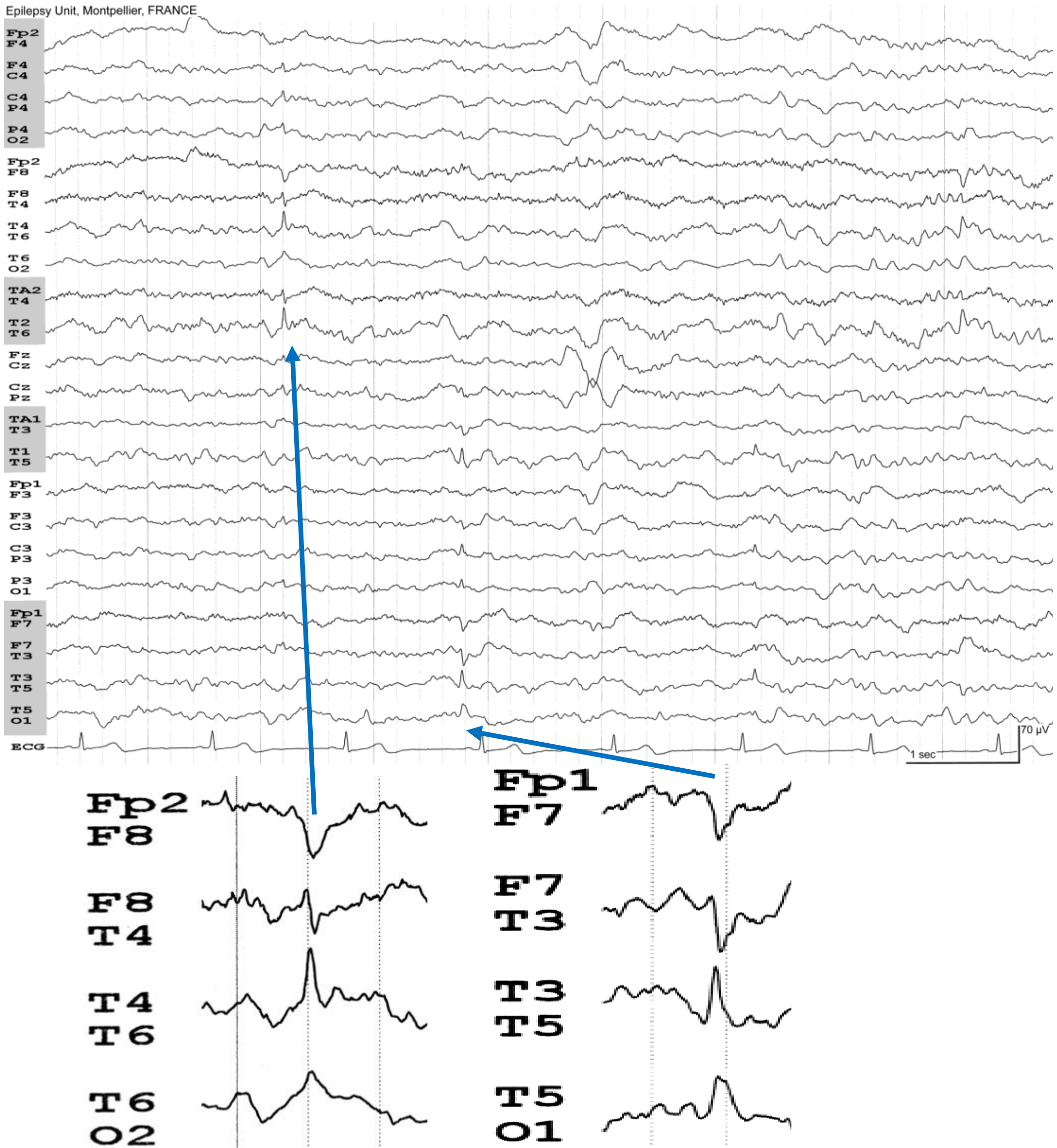

Small Sharp Spikes

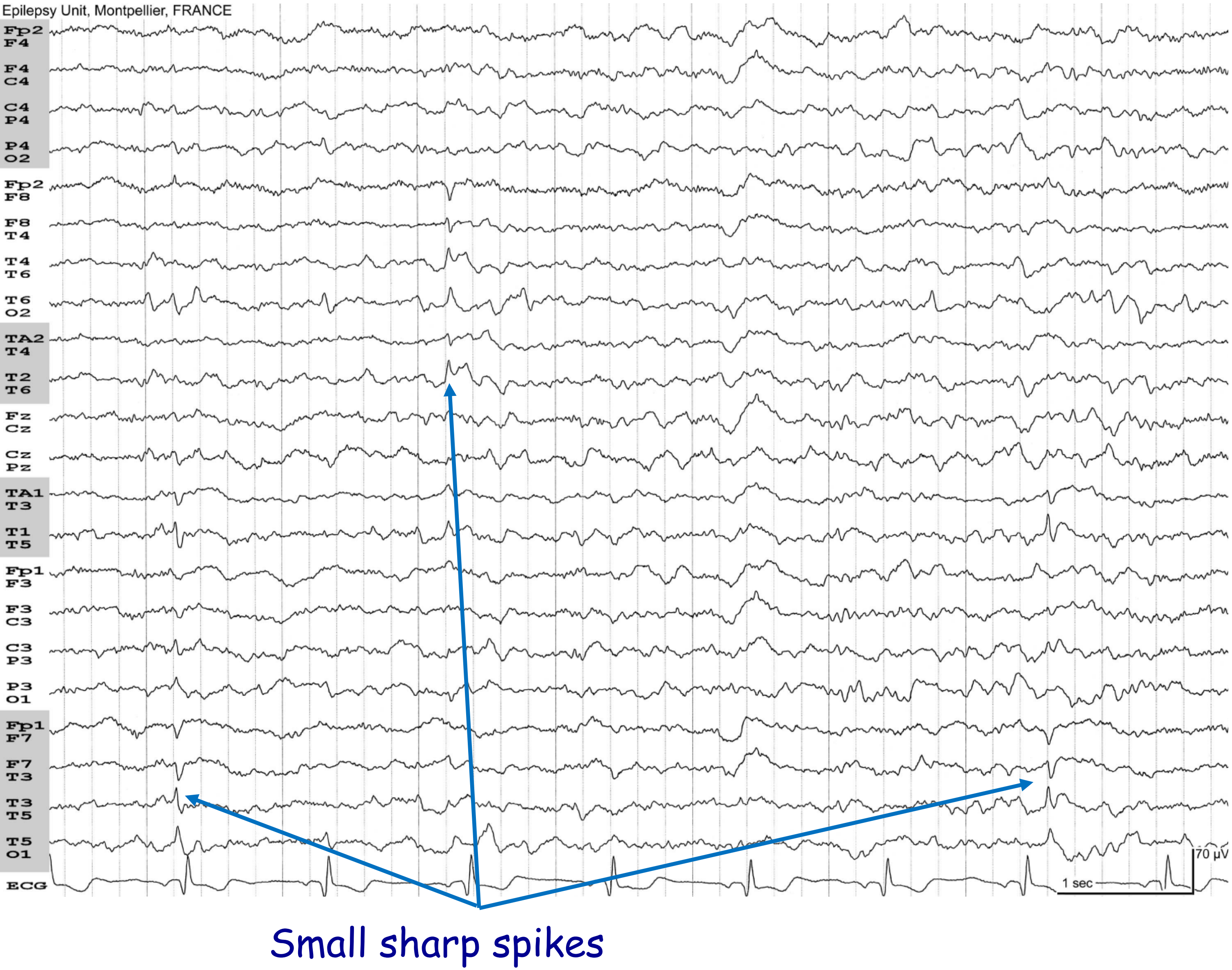

43-year-old man

Fourteen and 6-Hz positive bursts

- As indicated, these bursts occur at a rate of 6 Hz and/or 14 Hz.
- They consist of short trains of low-voltage arch-shaped waveforms.
- They occur during drowsiness and light sleep but are mainly seen during REM sleep.
- They appear in children after 3-4 years, are maximally expressed in the adolescents and young adults, and then progressively decrease in adulthood.

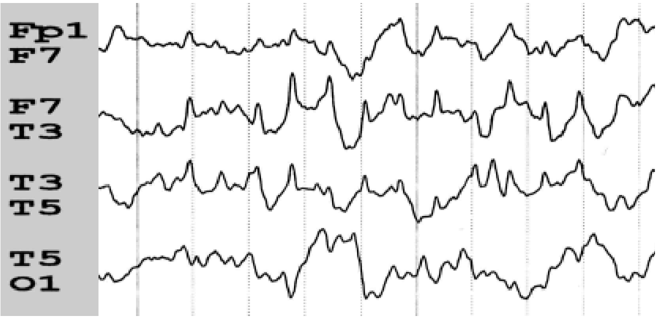

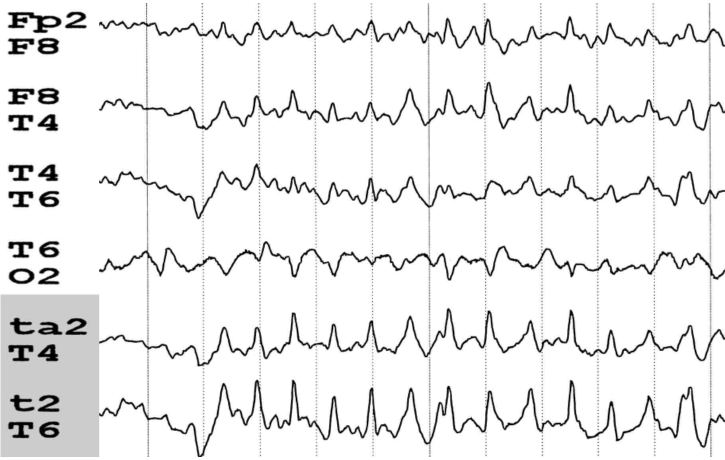

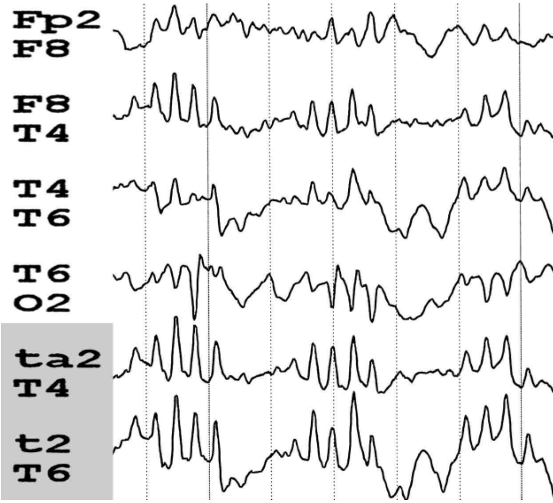

30 mm/second

Fourteen and 6-Hz positive bursts

A new approach for the detection of the fourteen- and six-Hertz positive bursts (6–14 Hz): The lower temporal line

Reana Velizarova
Arielle Crespel
Anna Serafini
Philippe Gelisse *

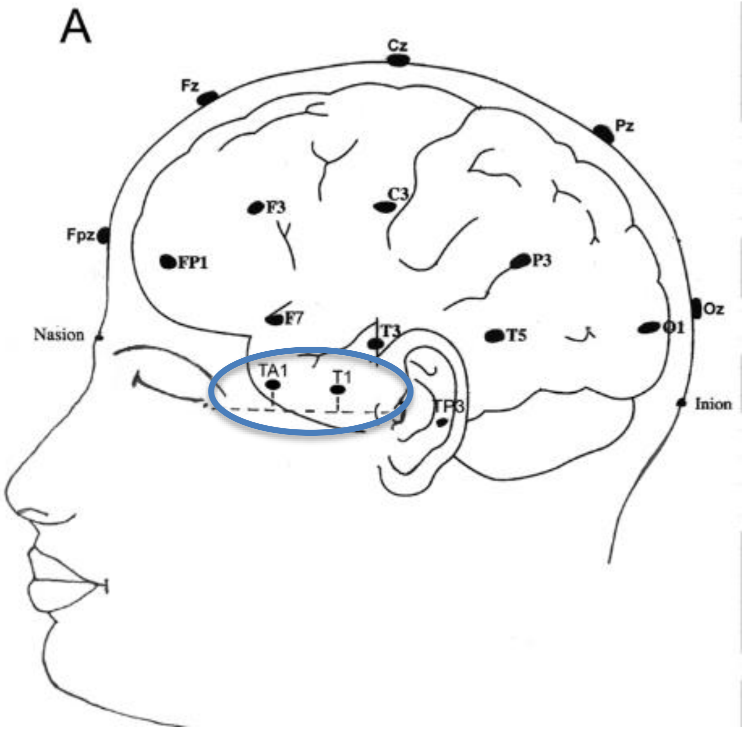

Letters to the Editor / Clinical Neurophysiology 122 (2011) 1268–1273

Table 1
Showing age distribution of the 50 patients with fourteen- and six-Hertz positive bursts seen in our epilepsy center from 2 January 2004 to 15 July 2010.

Age groups (In years)	Number of cases
5–10	2
11–14	3
15–20	21
21–25	10
26–30	4
31–40	5
41–50	4
51–55	1
Total	50

Fourteen and 6-Hz positive bursts during REM sleep in an adult

19-year-old man with psychogenic seizures

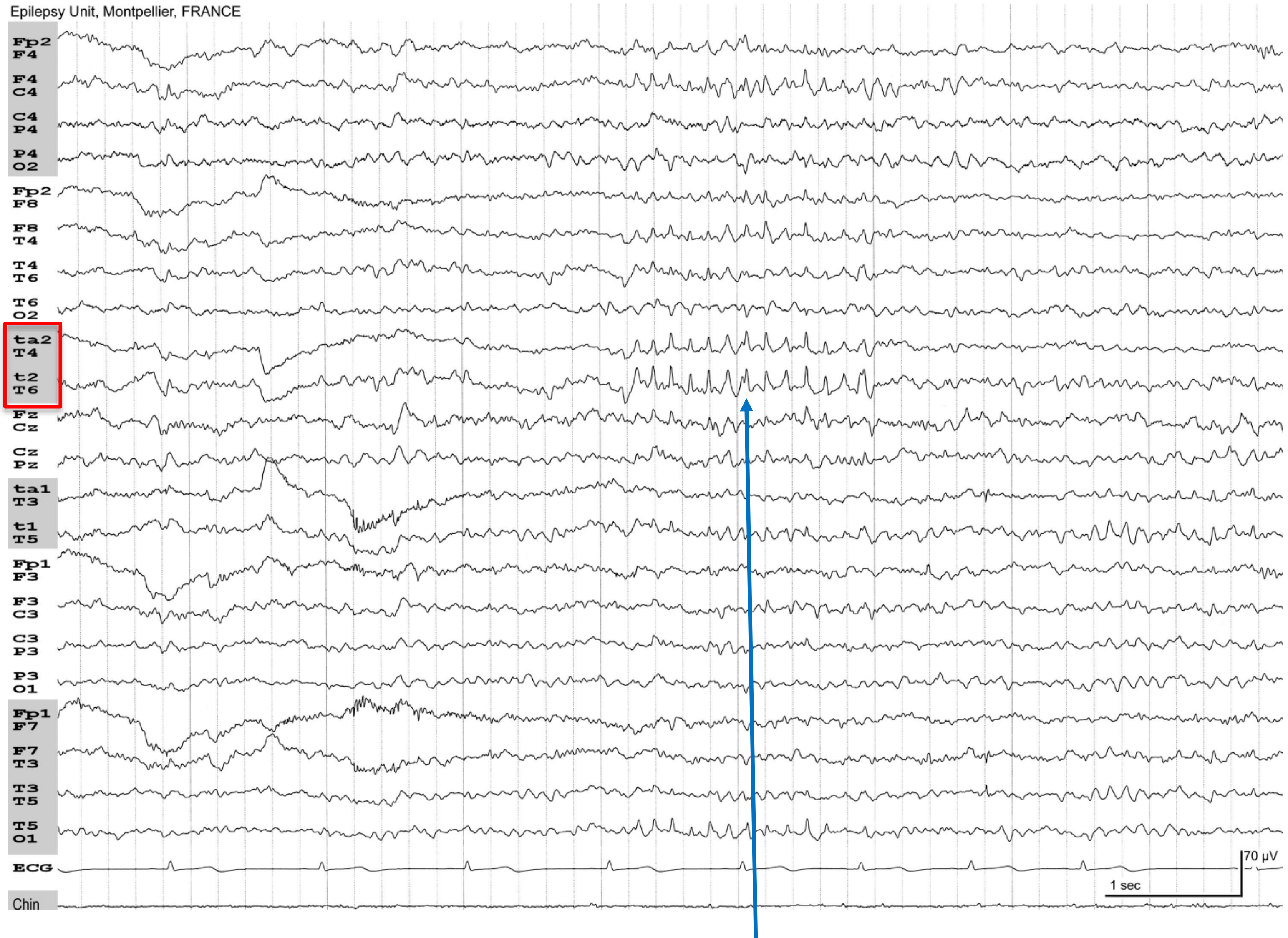

14- and 6-Hz positive burst during REM sleep

Fourteen and 6-Hz positive bursts during REM sleep in an adult

18-year-old woman with idiopathic (genetic) generalized epilepsy

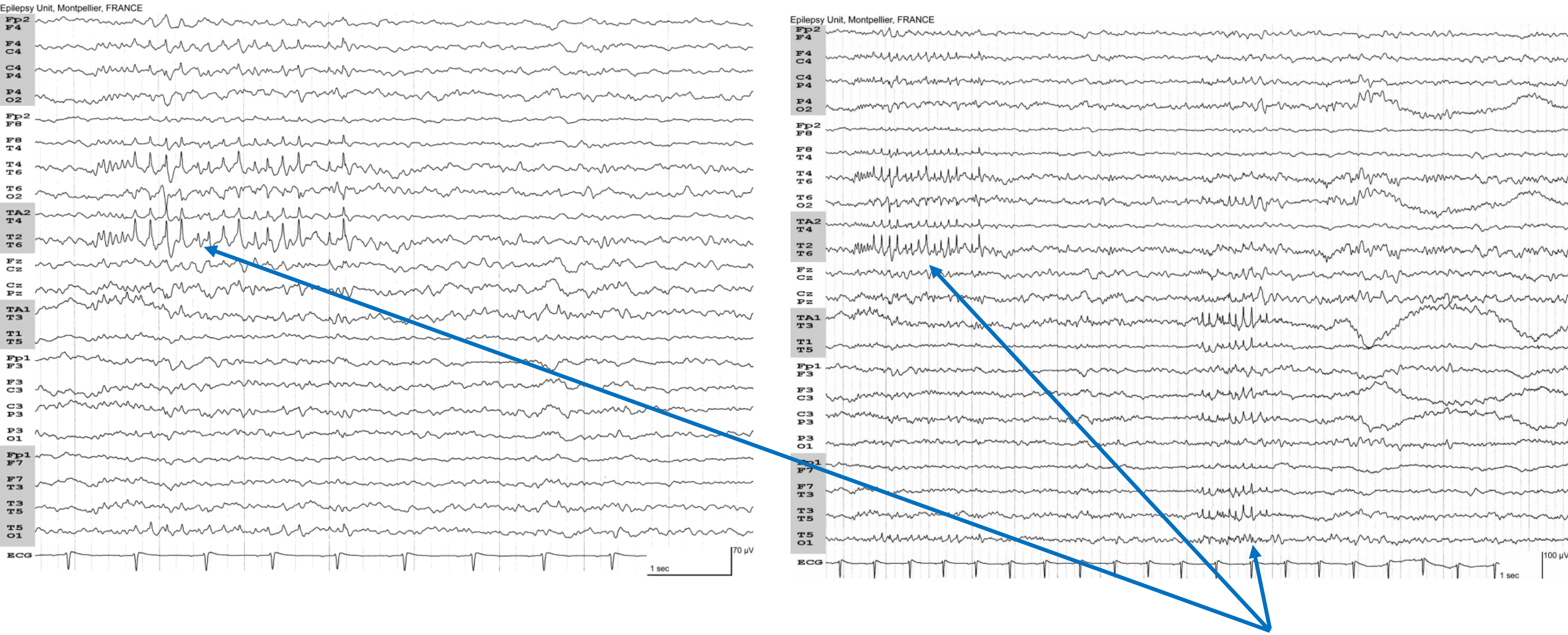

14- and 6-Hz positive burst during REM sleep

Fourteen and 6-Hz positive bursts

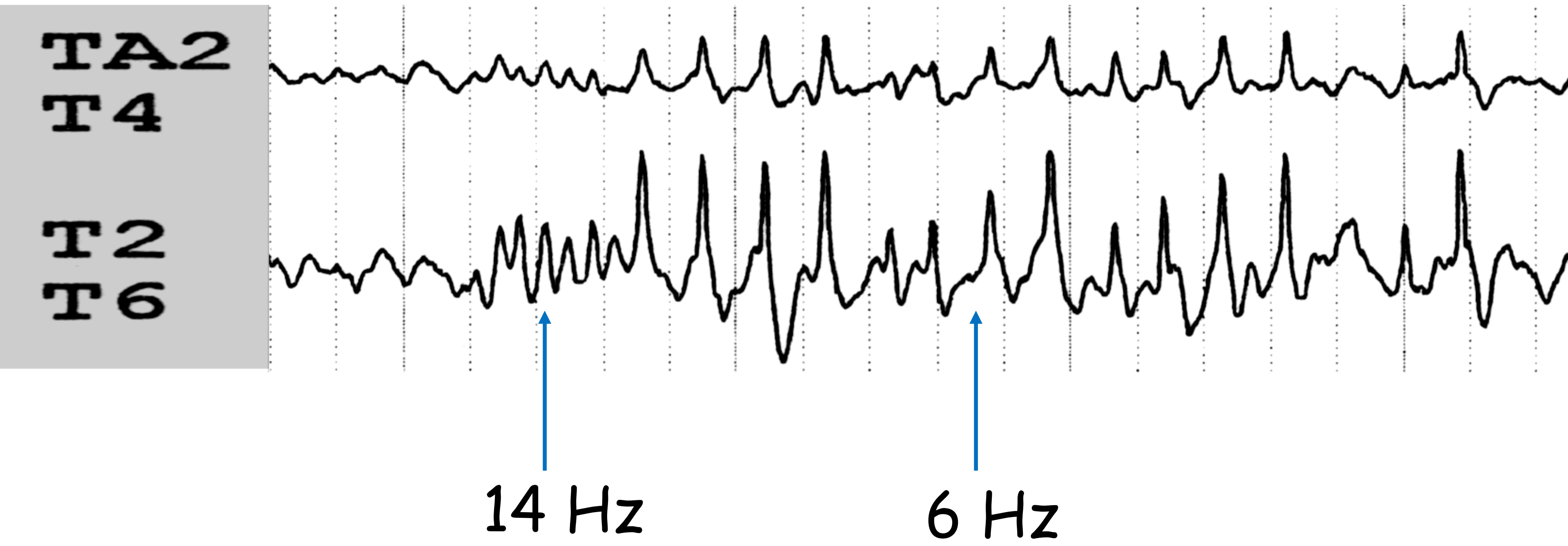

Fourteen and 6-Hz positive bursts during NREM sleep in an adult

18-year-old woman with idiopathic (genetic) generalized epilepsy

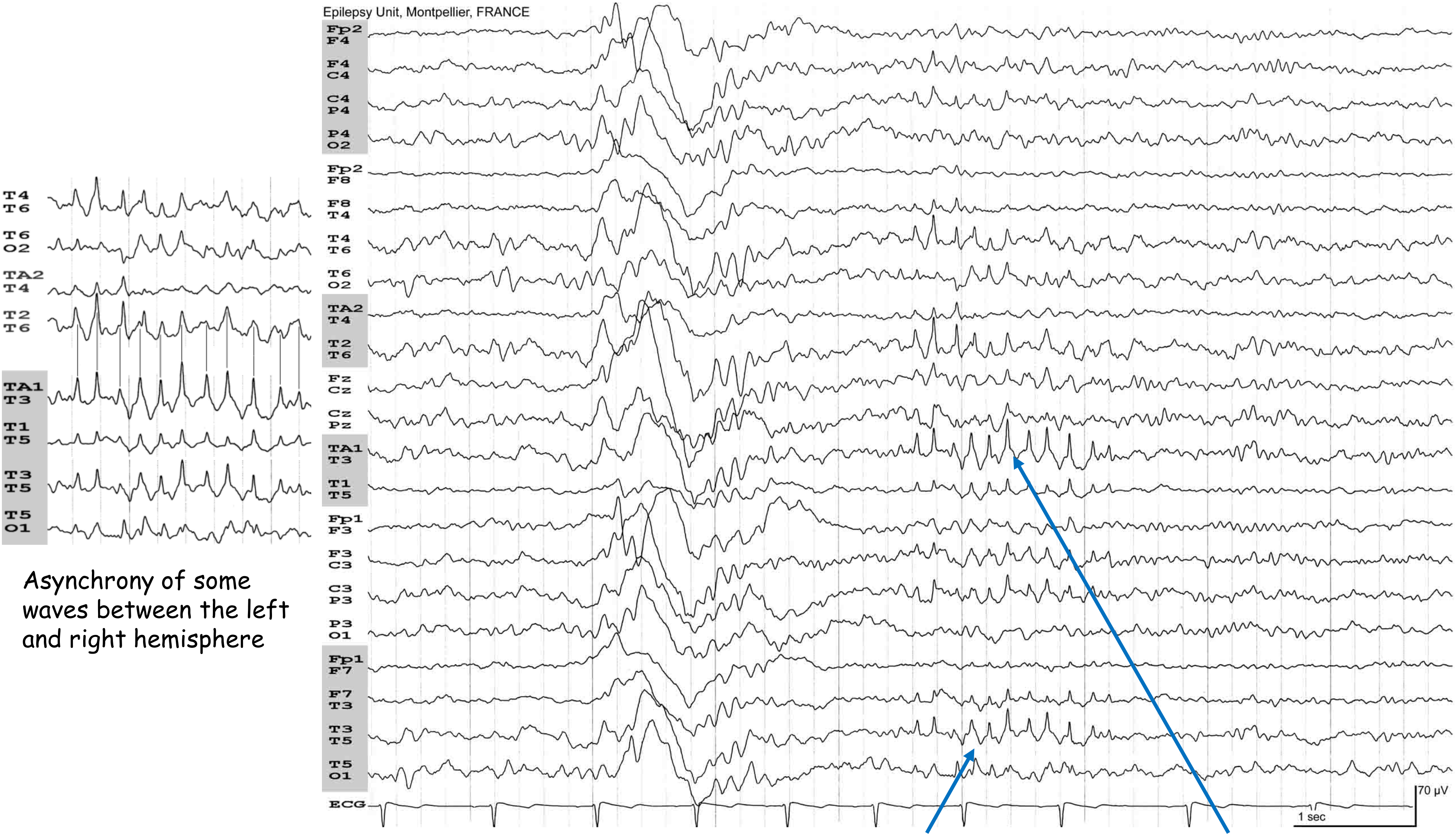

Asynchrony of some waves between the left and right hemisphere

14- and 6-Hz positive burst during NREM sleep

Subclinical Rhythmic Electrographic Discharges in Adults (SREDA)

- SREDA first described by Naquet et al. (1961) using the term of "*paroxystic discharges of the parietal and posterior temporal regions*"
- Onset is abrupt or the onset involves a built-up of slow waves gradually occurring at shorter intervals.
- Pattern frequency at 4-7 Hz is sustained, lasting from a few seconds to a few minutes
- Occur during wakefulness, may be activated by hyperventilation, or photic stimulation. May be observed in NREM and REM sleep
- Occur several times in a single standard EEG

Subclinical Rhythmic Electrographic Discharges in Adults (SREDA) in NREM sleep stage 2

49-year-old woman with psychogenic seizures

K complex

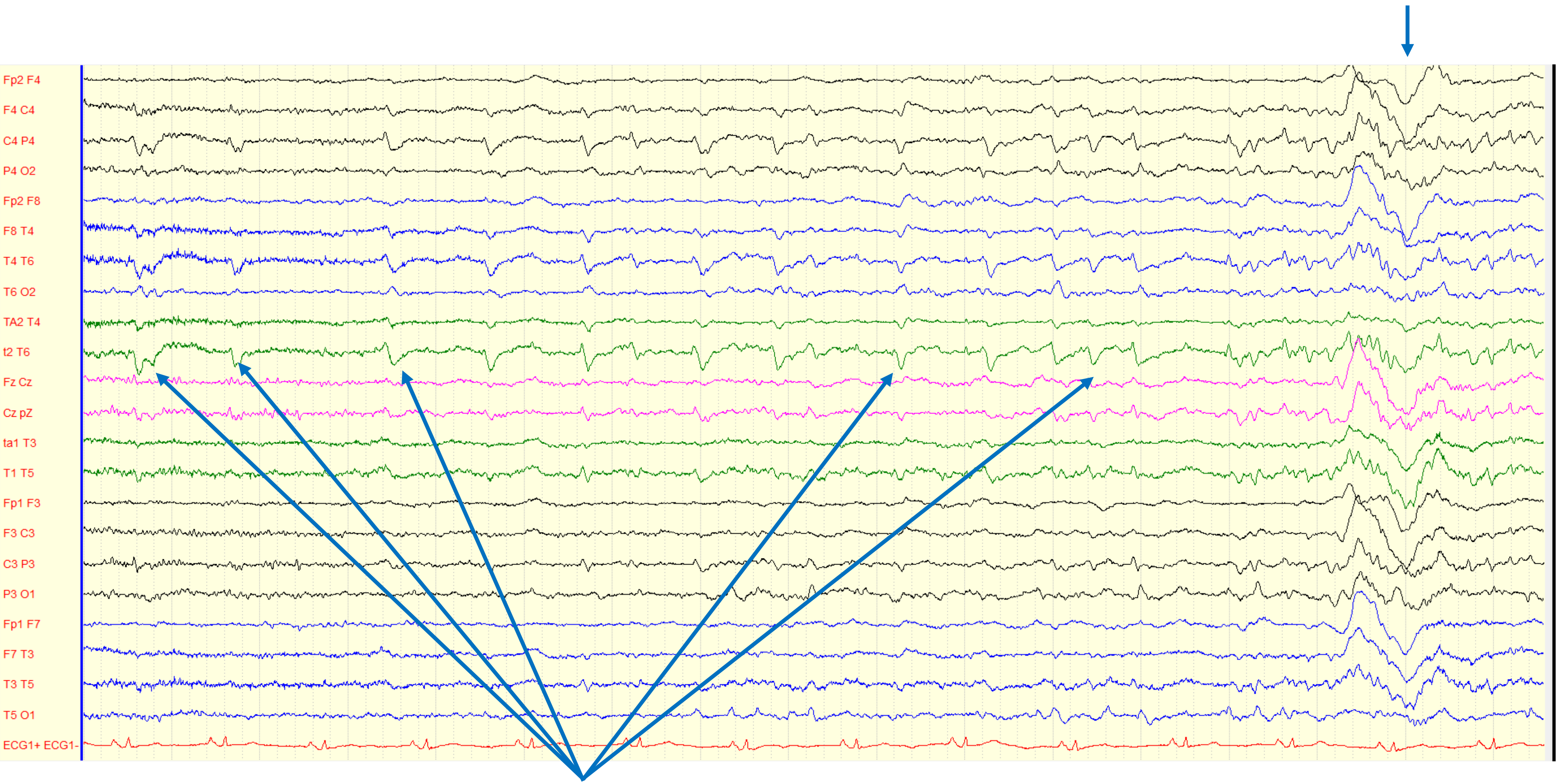

Slow waves appear on the right posterior region at gradually shorter interval until there is a continuous discharge

NREM sleep
30 mm/s

Subclinical Rhythmic Electrographic Discharges in Adults (SREDA) in NREM sleep stage 2

49-year-old woman with psychogenic seizures

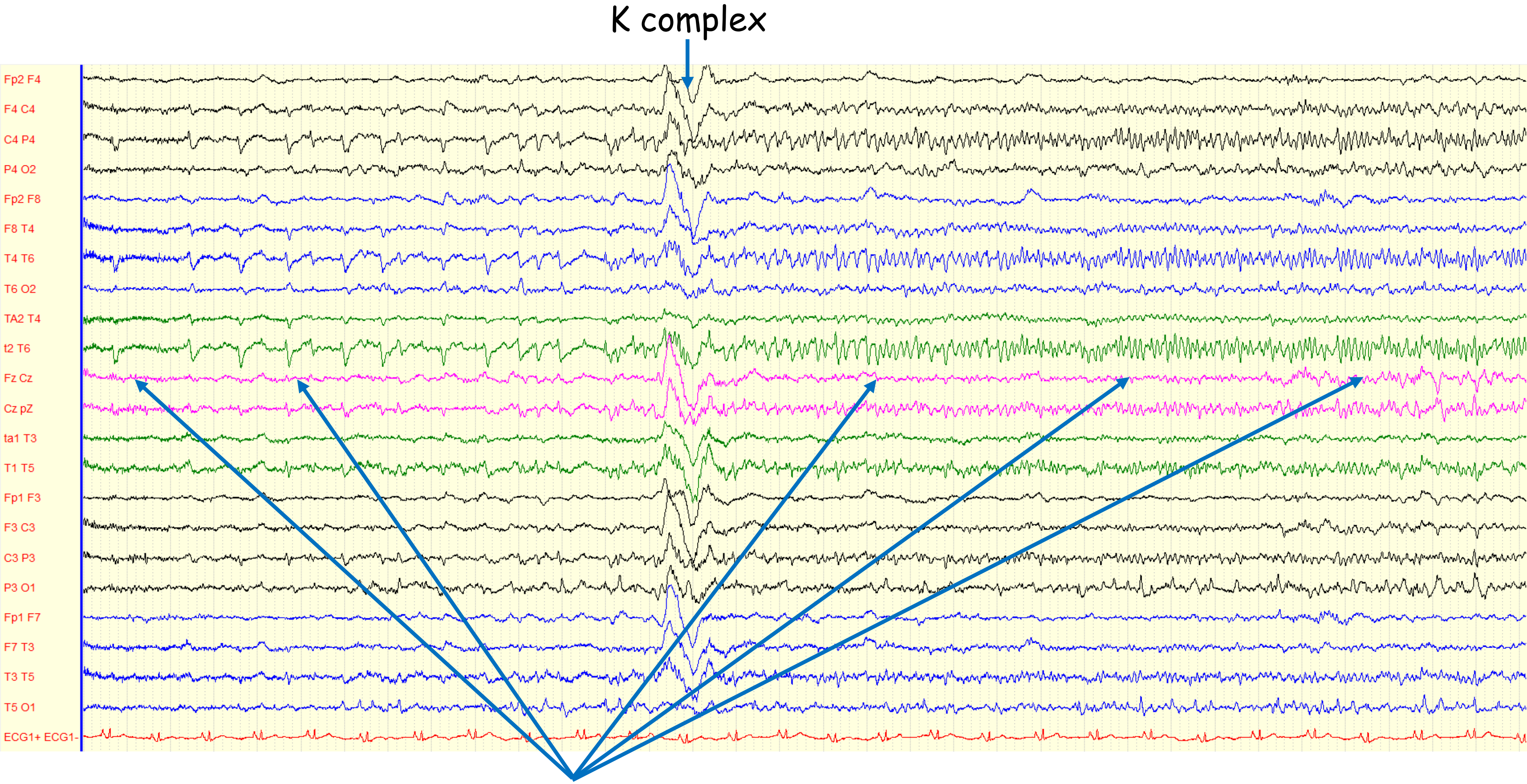

Slow waves appear on the right posterior region at gradually shorter interval until there is a continuous discharge

Same EEG panel at an EEG speed of 15 mm/s

NREM sleep
15 mm/s

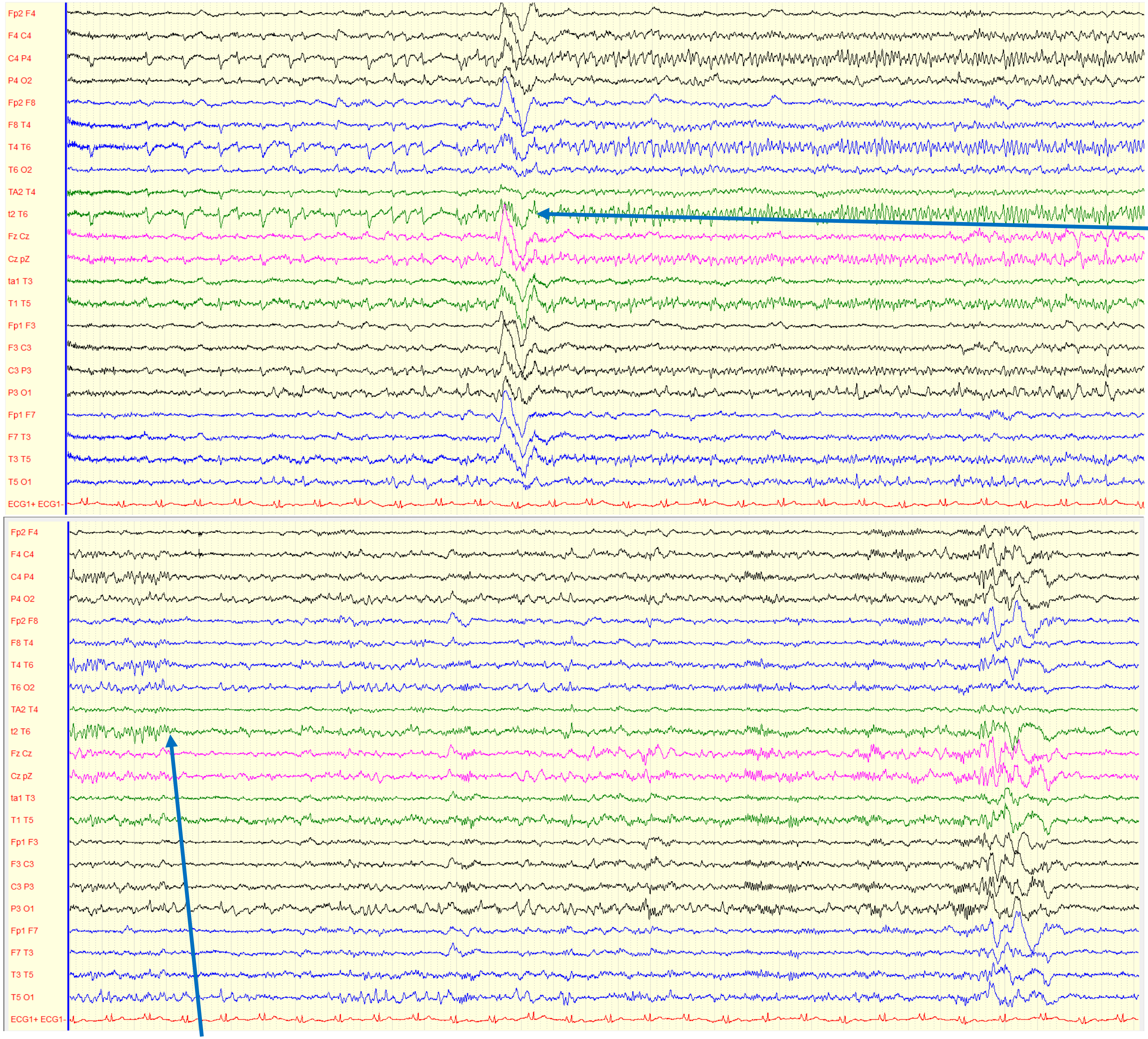

Pay attention to the K complex present inside the discharge

End of the discharge
No arousal

NREM sleep
15 mm/s

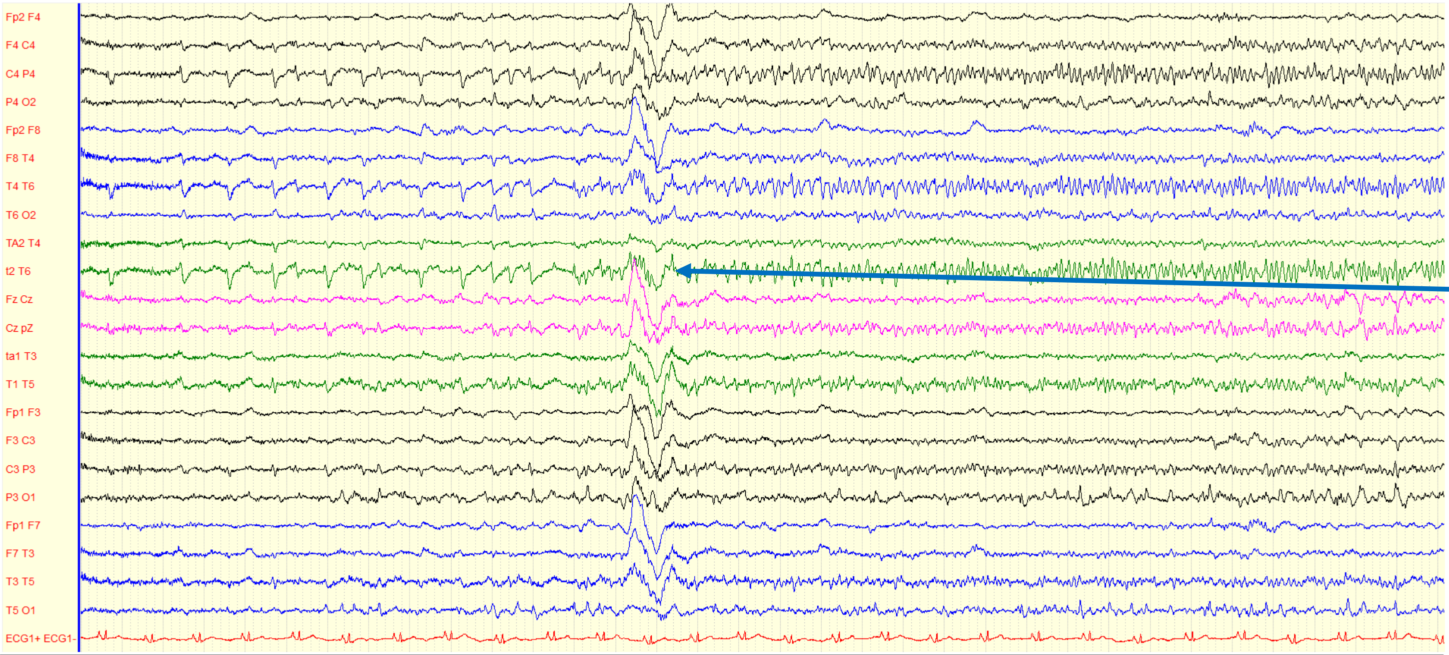

Pay attention to the K complex present inside the discharge

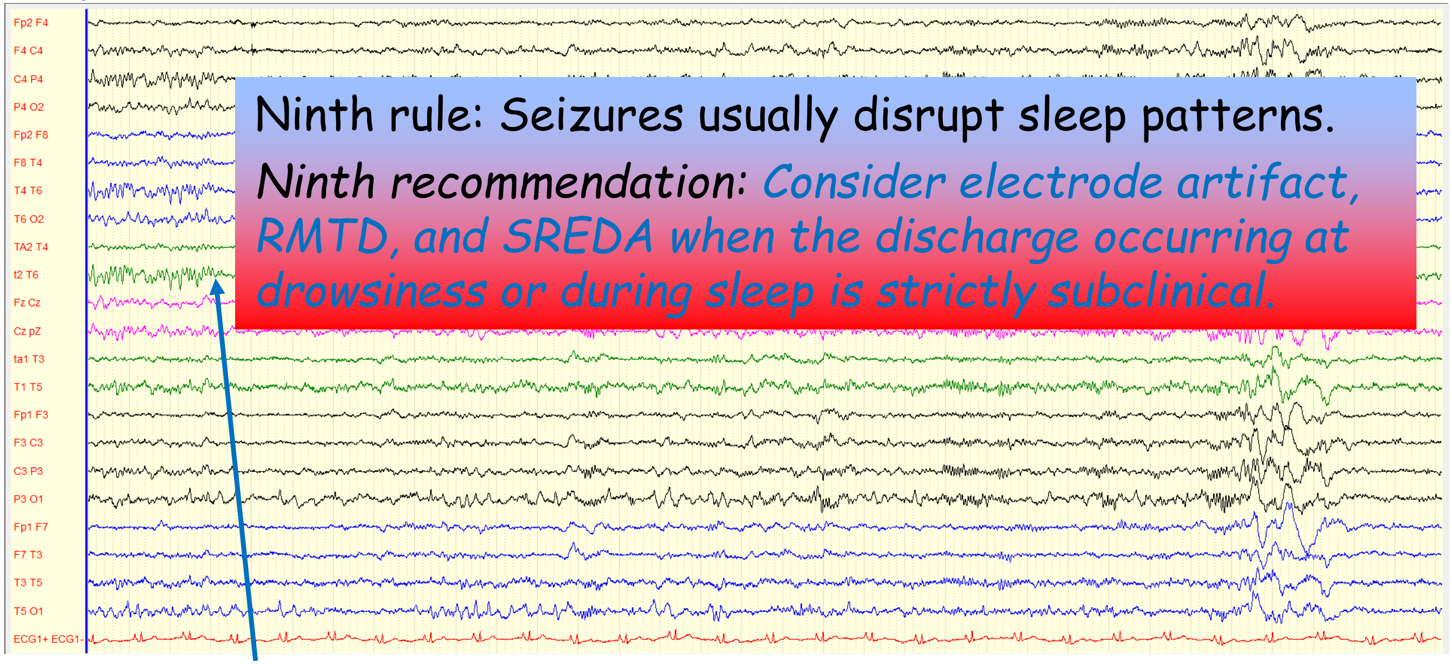

Ninth rule: Seizures usually disrupt sleep patterns.

Ninth recommendation: Consider electrode artifact, RMTD, and SREDA when the discharge occurring at drowsiness or during sleep is strictly subclinical.

End of the discharge
No arousal

NREM sleep
15 mm/s

Subclinical Rhythmic Electrographic Discharges in Adults (SREDA) in REM sleep

49-year-old woman with psychogenic seizures. Same patient.

No arousal

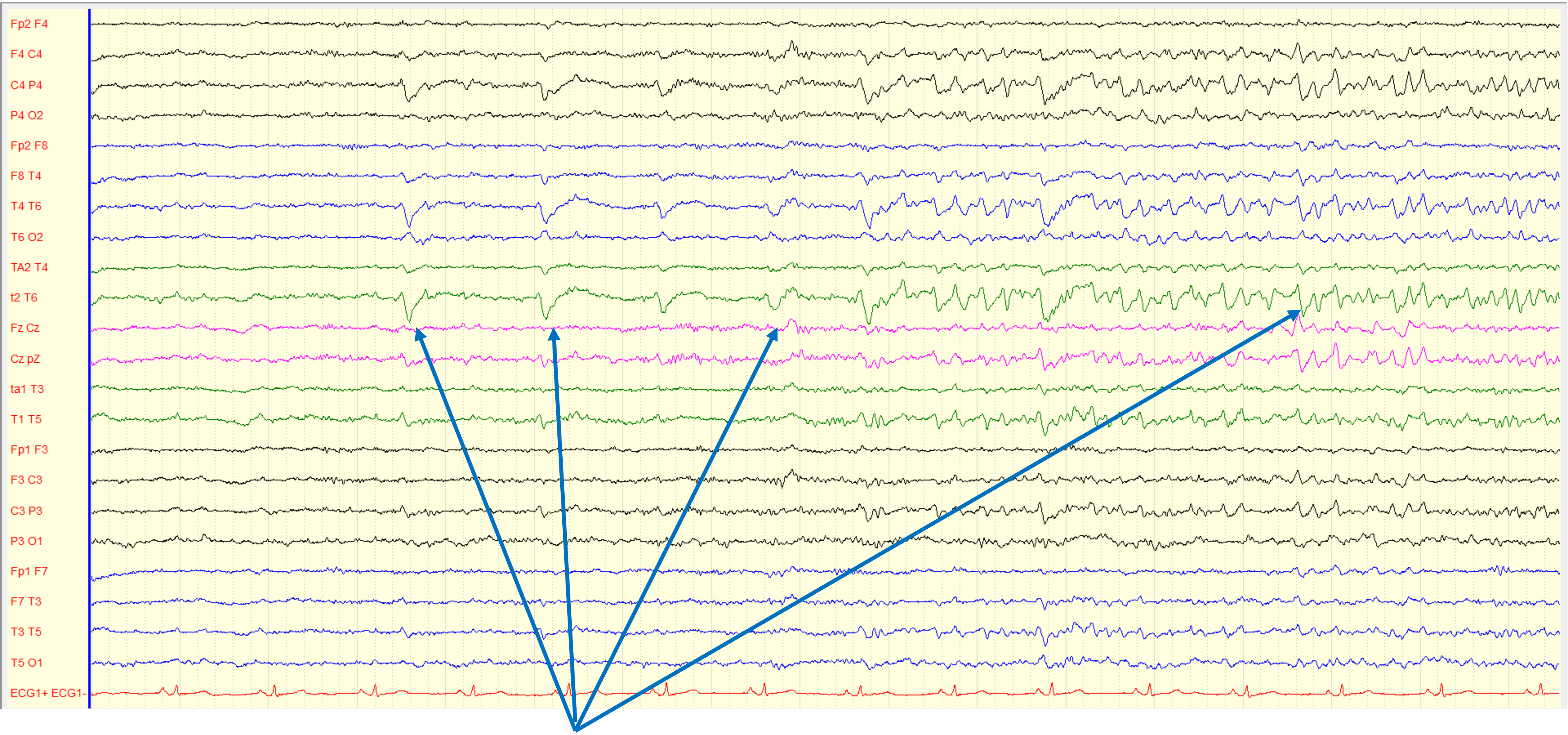

Slow waves appear on the right posterior region at gradually shorter interval until there is a continuous discharge

REM sleep
30 mm/s

Subclinical Rhythmic Electrographic Discharges in Adults (SREDA) in REM sleep

49-year-old woman with psychogenic seizures. Same patient.

Rapid eye movements

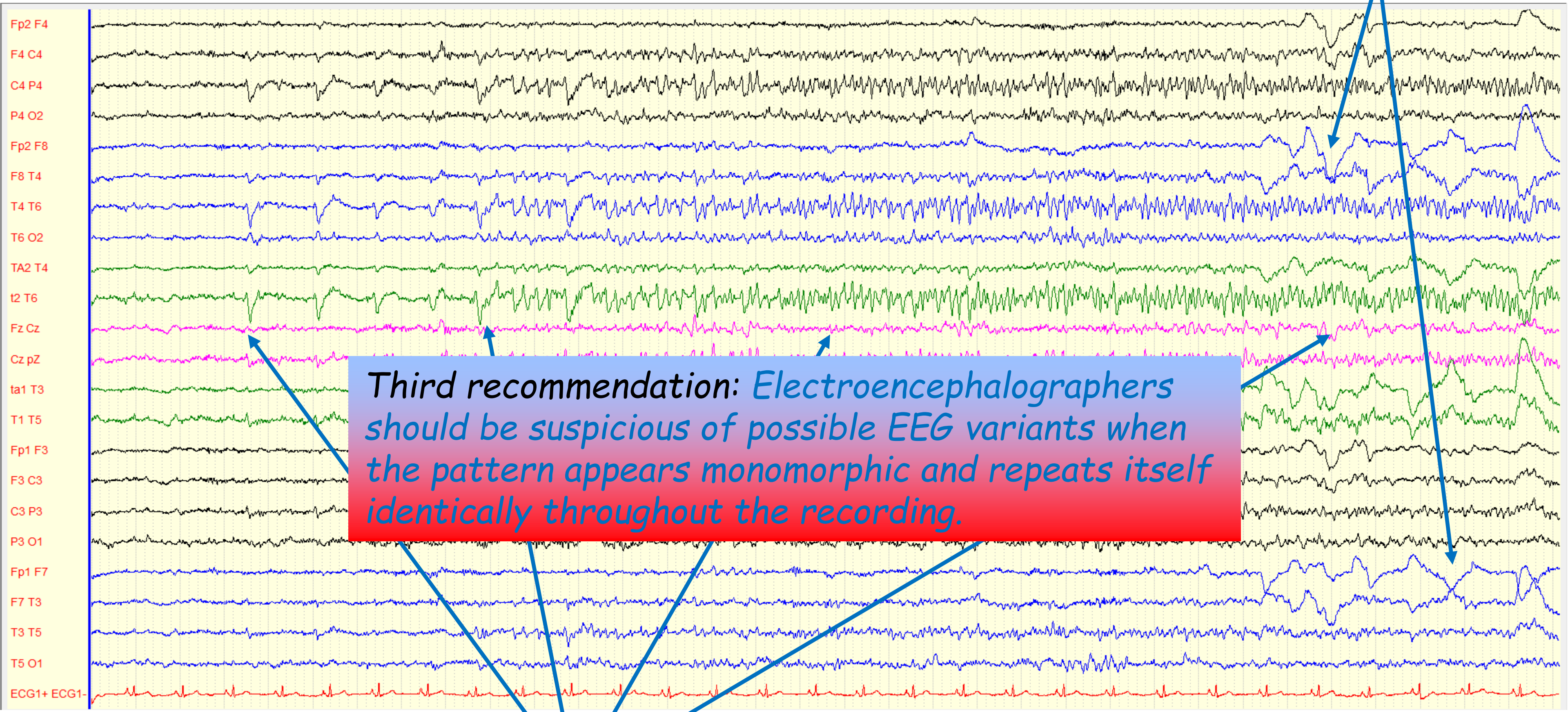

Third recommendation: Electroencephalographers should be suspicious of possible EEG variants when the pattern appears monomorphic and repeats itself identically throughout the recording.

Slow waves appear on the right posterior region at gradually shorter interval until there is a continuous discharge

No arousal

REM sleep
15 mm/s

Same EEG panel at an EEG speed of 15 mm/s

Subclinical Rhythmic Electrographic Discharges in Adults (SREDA) in REM sleep

49-year-old woman with psychogenic seizures. Same patient.

Rapid eye movements

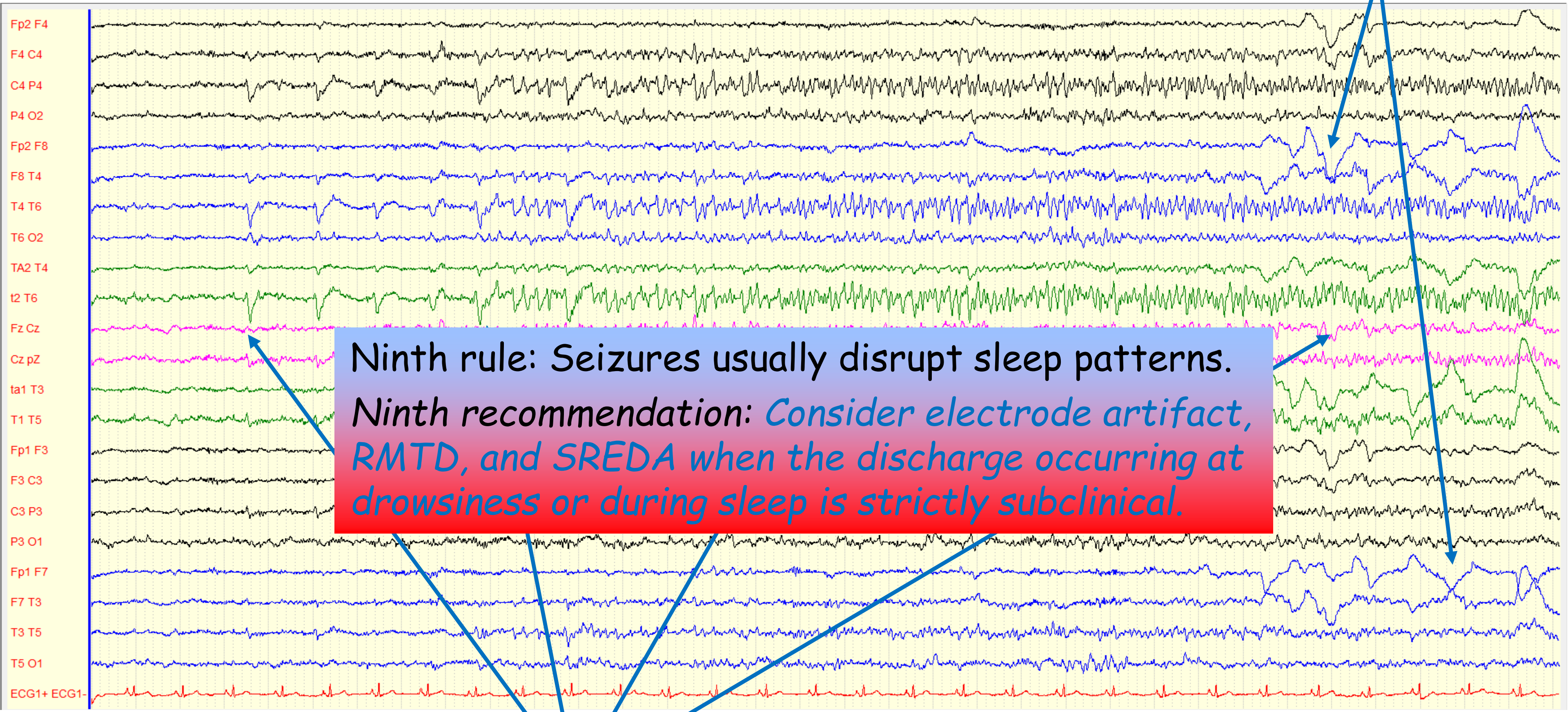

Ninth rule: Seizures usually disrupt sleep patterns.

Ninth recommendation: Consider electrode artifact, RMTD, and SREDA when the discharge occurring at drowsiness or during sleep is strictly subclinical.

No arousal

REM sleep
15 mm/s

Slow waves appear on the right posterior region at gradually shorter interval until there is a continuous discharge

Same EEG panel at an EEG speed of 15 mm/s

Subclinical Rhythmic Electrographic Discharges in Adults (SREDA)

37-year-old woman with right temporal lobe epilepsy

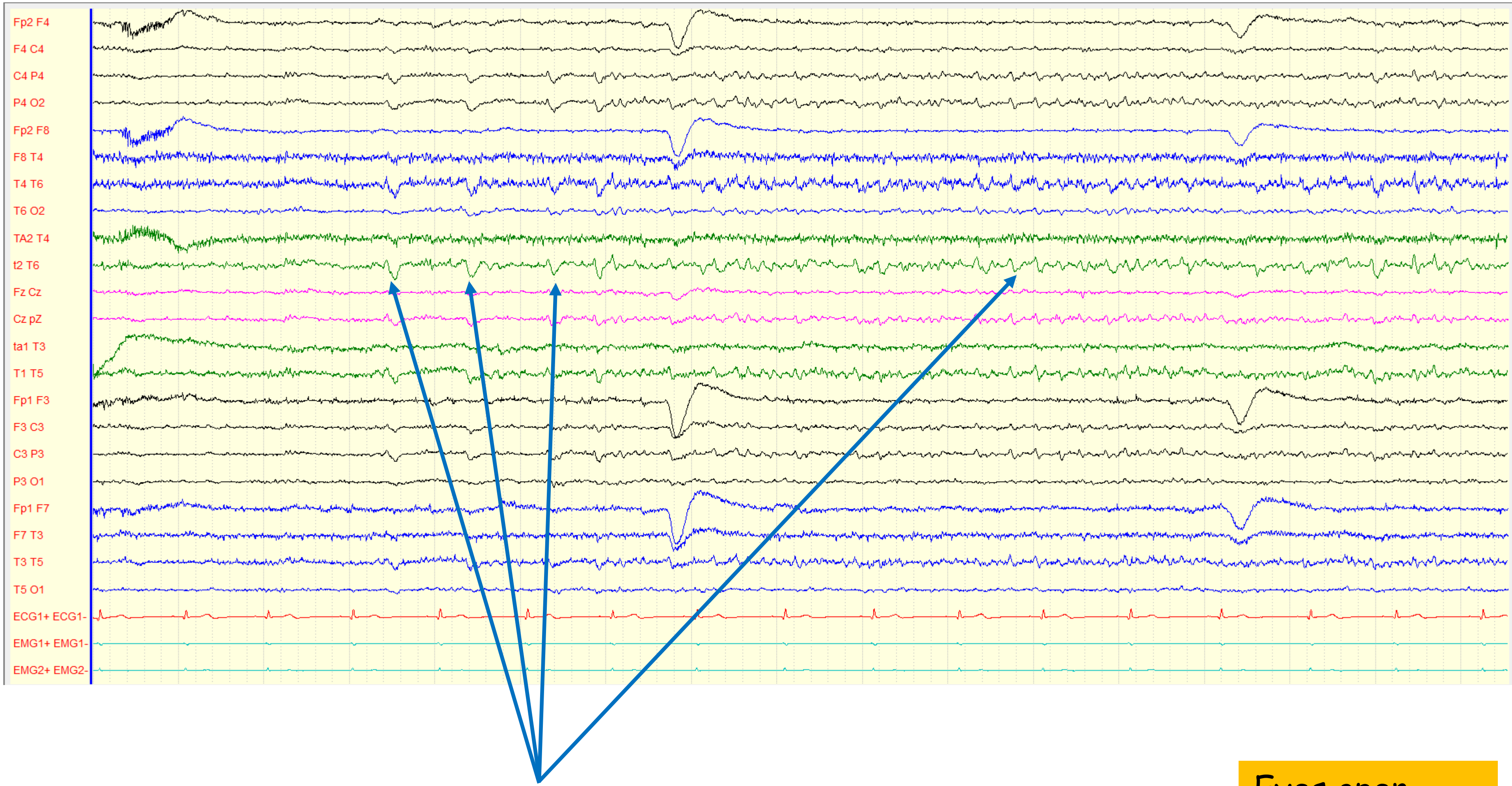

Slow waves appear on the right posterior region at gradually shorter interval until there is a continuous discharge

Eyes open
30 mm/s

Subclinical Rhythmic Electrographic Discharges in Adults (SREDA)

37-year-old woman with right temporal lobe epilepsy

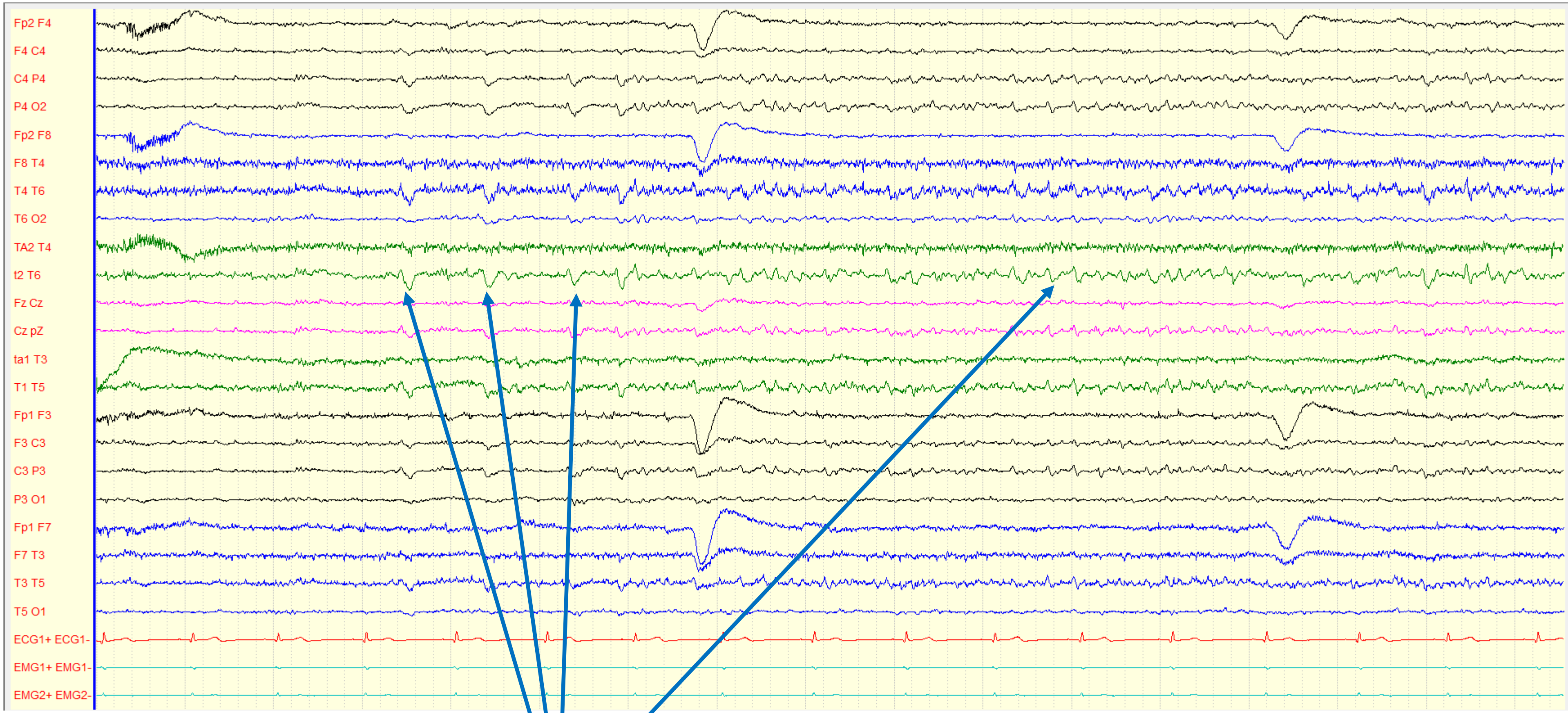

Slow waves appear on the right posterior region at gradually shorter interval until there is a continuous discharge

Eyes open
30 mm/s

Subclinical Rhythmic Electrographic Discharges in Adults (SREDA)

37-year-old woman with right temporal lobe epilepsy

Same EEG panel at an EEG speed of 15 mm/s

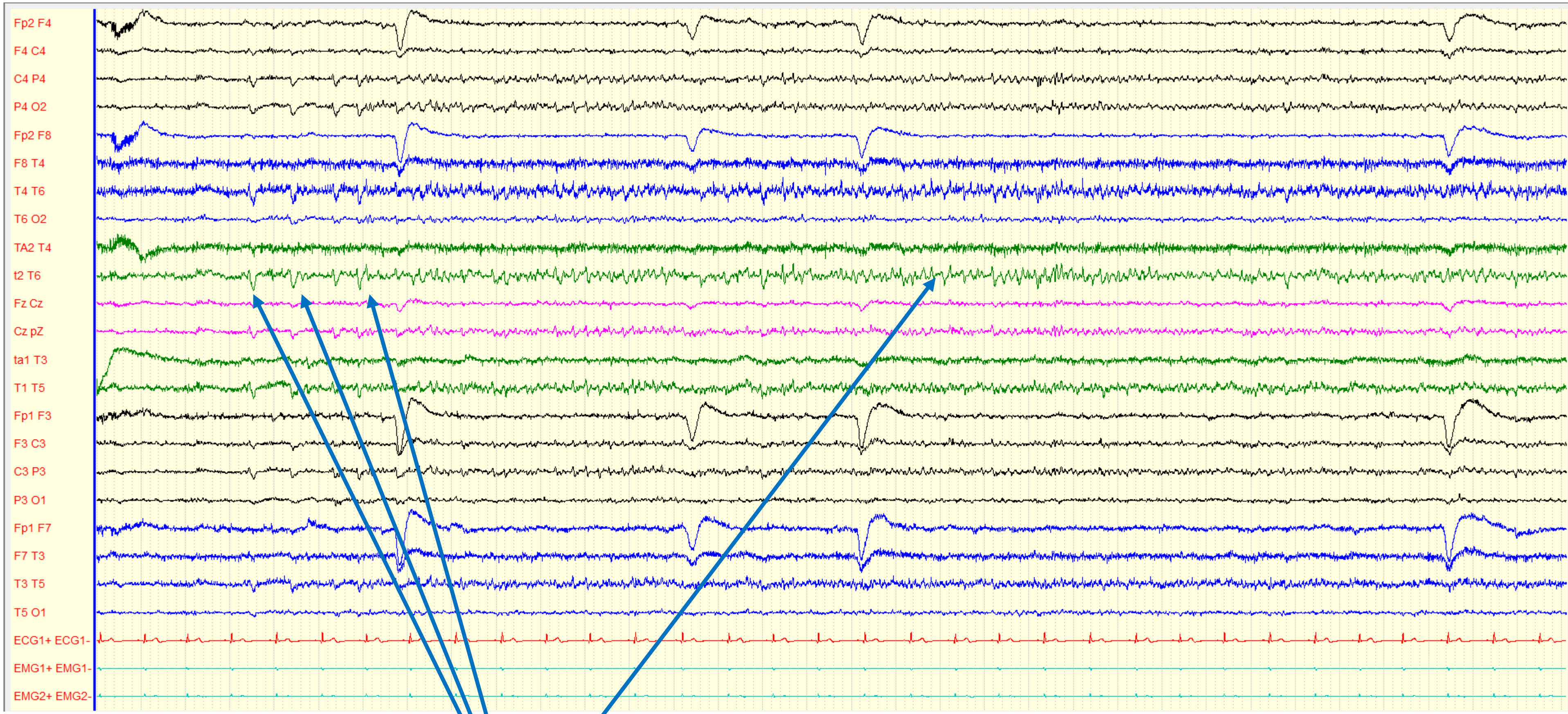

Slow waves appear on the right posterior region at gradually shorter interval until there is a continuous discharge

Eyes open
15 mm/s

Subclinical Rhythmic Electrographic Discharges in Adults (SREDA)

37-year-old woman with right temporal lobe epilepsy

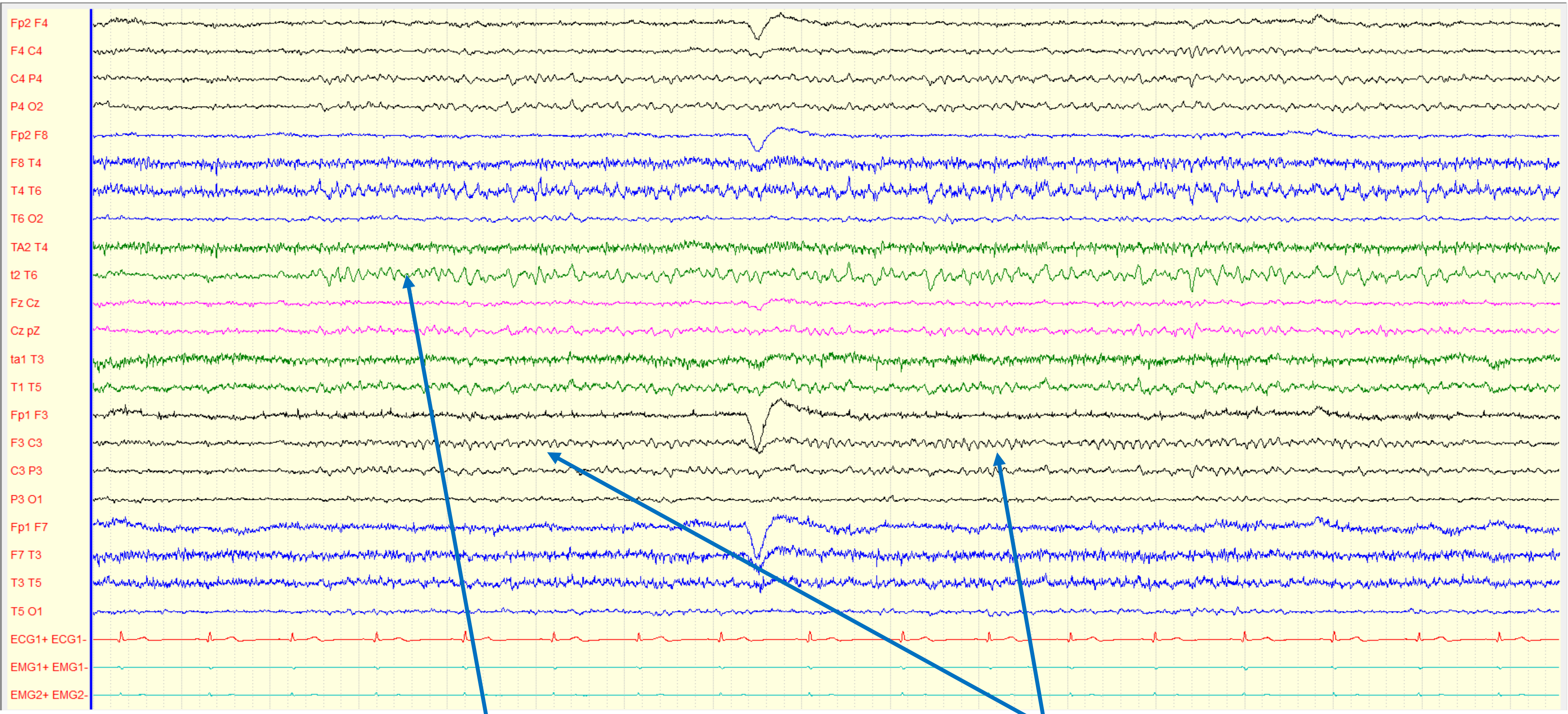

Mu rhythm

Another example of SREDA in the same patient but without build-up of slow waves gradually occuring at shorter intervals

Eyes open
30 mm/s

Subclinical Rhythmic Electrographic Discharges in Adults (SREDA) in REM Sleep

37-year-old woman with right temporal lobe epilepsy

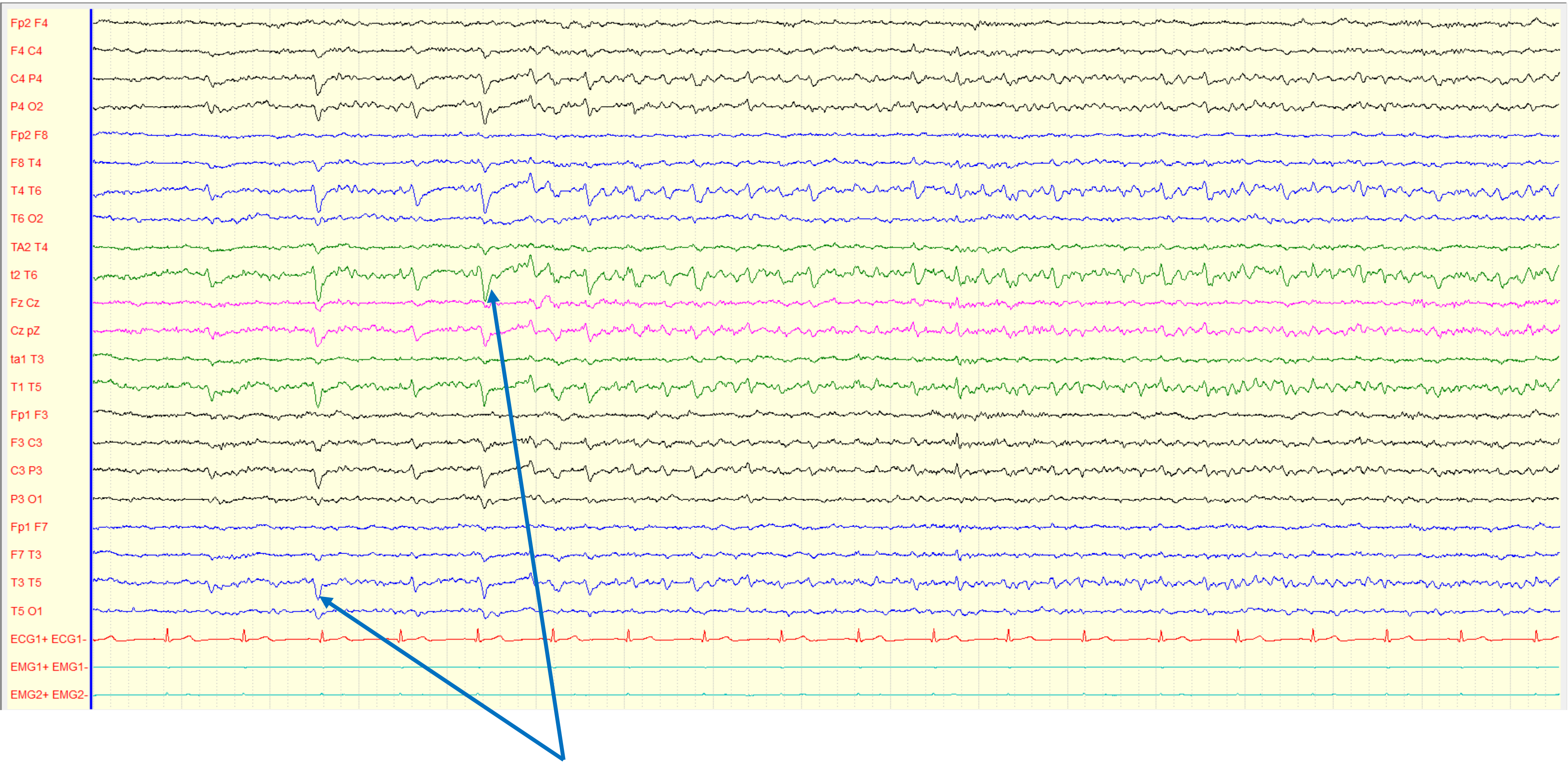

Same patient. Slow waves appears bilaterally in REM sleep at gradually shorter intervals until there is a continuous discharge

REM Sleep
30 mm/s

Subclinical Rhythmic Electrographic Discharges in Adults (SREDA) in REM Sleep

37-year-old woman with right temporal lobe epilepsy

Same EEG panel at an EEG speed of 15 mm/s

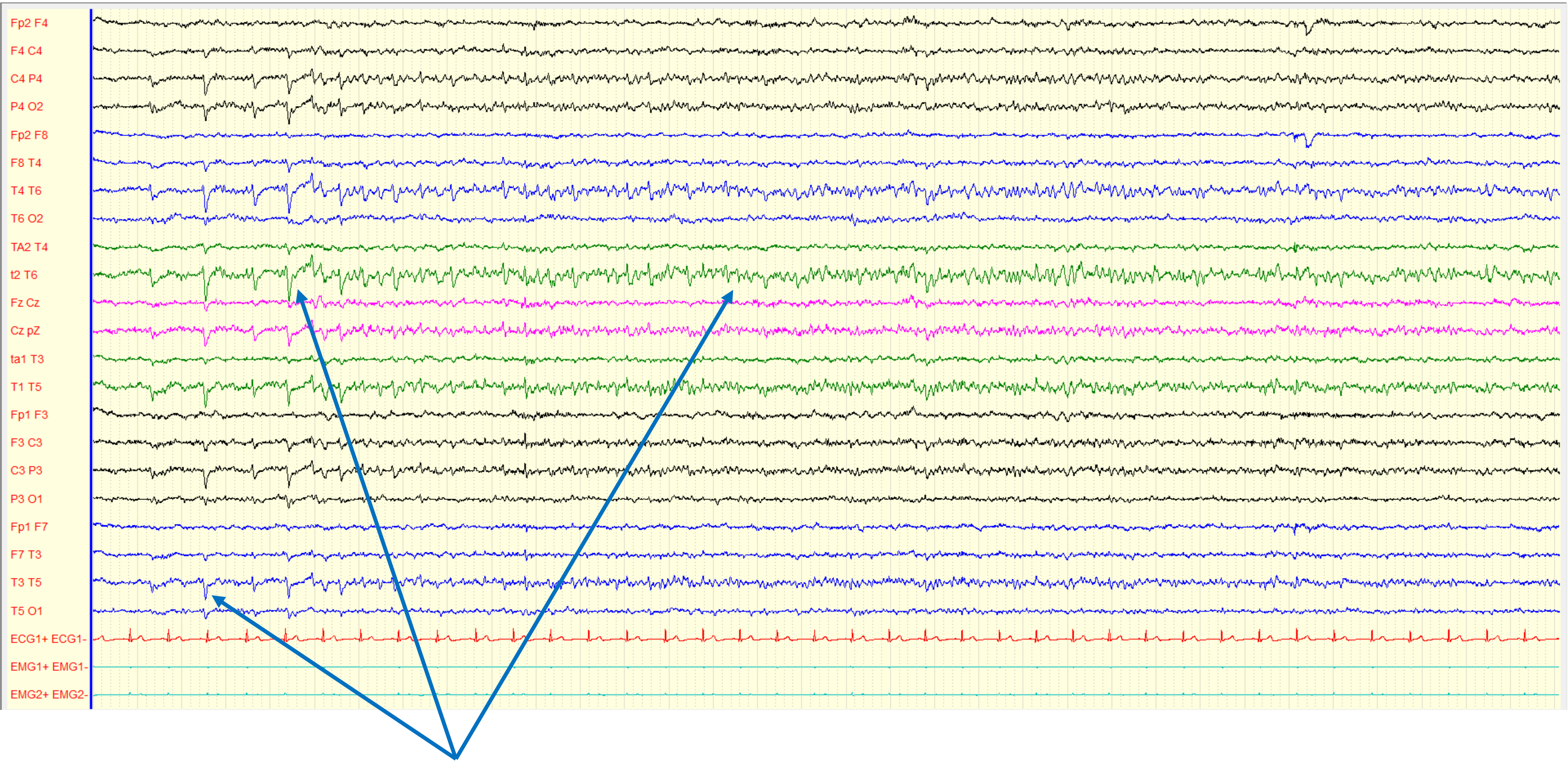

Same patient. Slow waves appears bilaterally in REM sleep at gradually shorter intervals until there is a continuous discharge

REM Sleep
15 mm/s

Temporal lobe seizure in NREM sleep

Same patient. One focal subclinal seizure during NREM sleep.

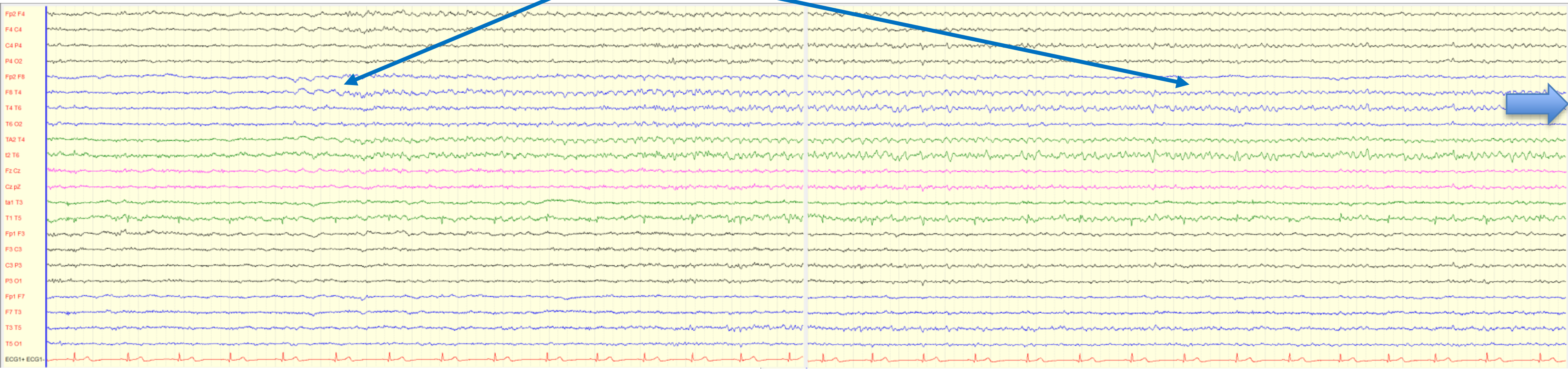

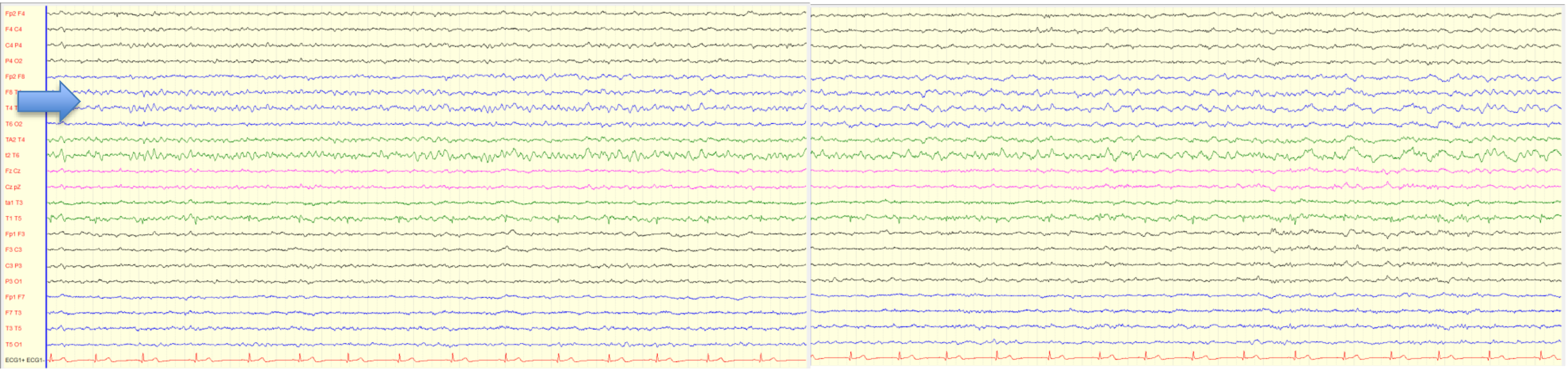

37-year-old woman with right temporal lobe epilepsy.

NREM Sleep
30 mm/s

Temporal lobe seizure in NREM sleep

37-year-old woman with right temporal lobe epilepsy. Same patient

End of the seizure

Post ictal phase with slow wave activity over the temporal region

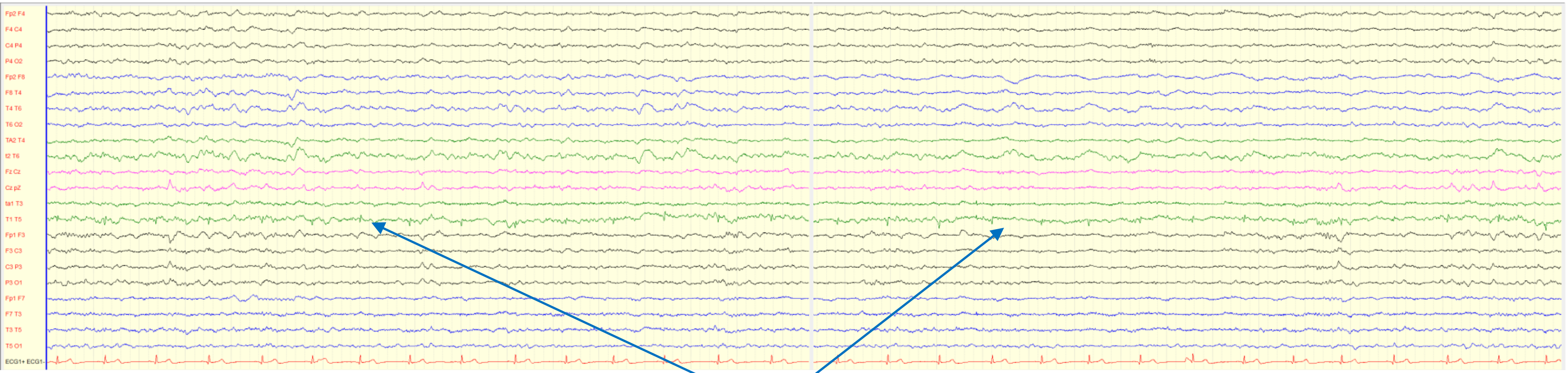

ECG artifact

This patient had both SREDA and temporal lobe epilepsy. Compare the evolution of the EEG pattern in both situations.

NREM Sleep
30 mm/s

Six-Hz Spike-and-Wave bursts (Phantom Spike-and-Wave bursts)

- Consist of brief bursts of spikes in very low amplitude with a repetition range of 5 to 7 Hz but usually occurring with a rate of 6 Hz.

- They are said to be "phantom" because of the low amplitude of the spike in contrast to the slow-wave component that follows and the more widespread distribution of the slow wave.

- This pattern usually occurs bilaterally, generally synchronous during relaxed wakefulness, drowsiness or light sleep.

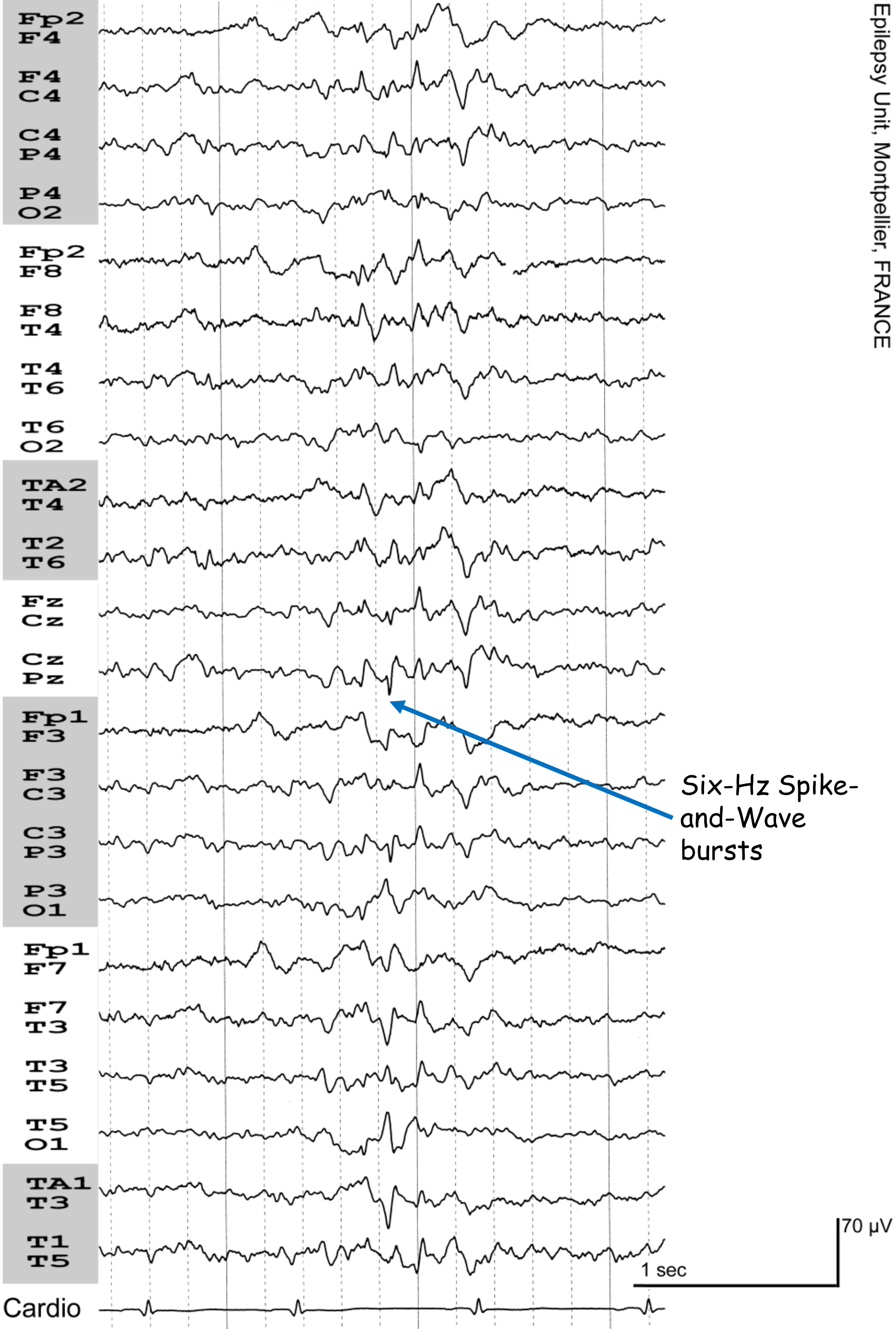

Breach rhythm

- A breach rhythm is an increase in high-voltage activity of alpha, beta, mu rhythm or other elements such as wicket spikes nearby the breach which occur in persons with a skull defect.

- Breach rhythm occur in wakefulness, and is persistent throughout all sleep stages.

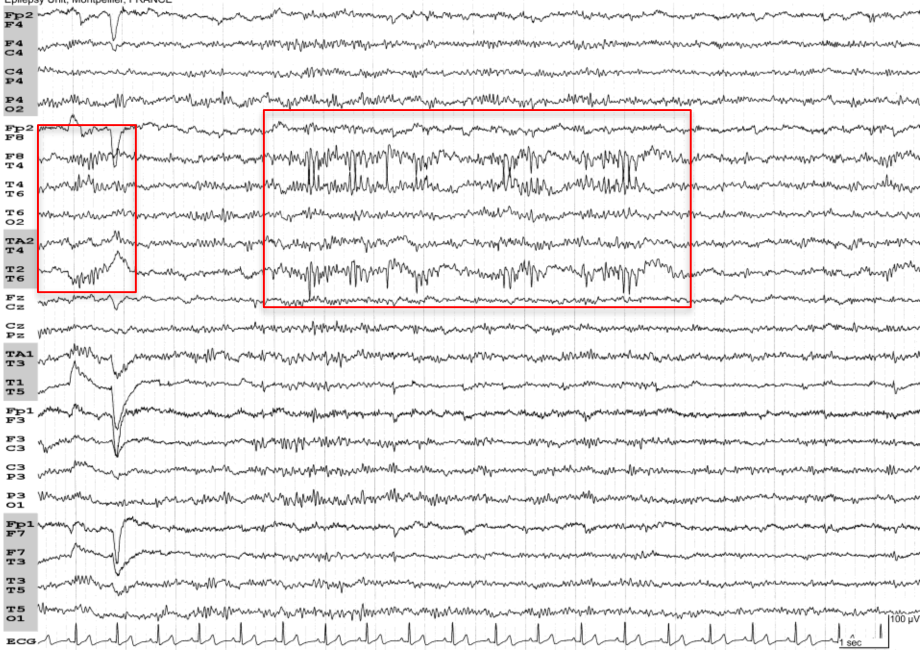

Breach rhythm

49-year-old woman.

5 years after surgery for a right cavernous sinus meningioma

Awake. Eye open

Typical breach rhythm with sharply-contoured waveforms that resemble to wicket-spikes

The morphology is stable with repetition of the same pattern throughout the entire EEG recording (Recommendation IV)

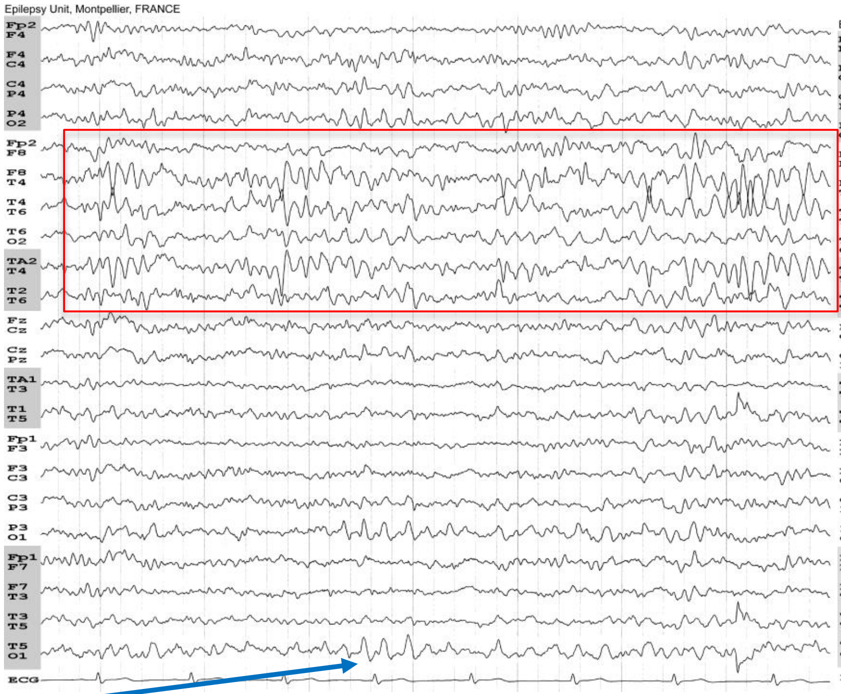

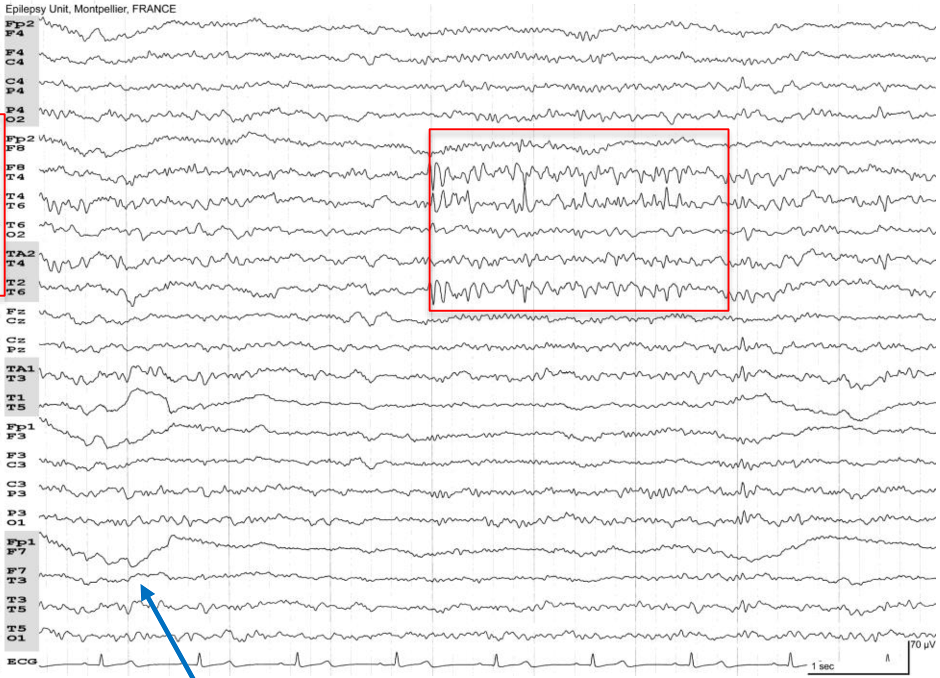

POSTs

Stage N2 sleep

Rapid eye movements

REM sleep

Tenth rule: Epilepsy is and must remain a clinical diagnosis

Tenth rule: Epilepsy is and must remain a clinical diagnosis

When the EEG makes things more difficult!

Typical spike-waves in subjects without epilepsy

3-year-old boy with a typical vasovagal syncope

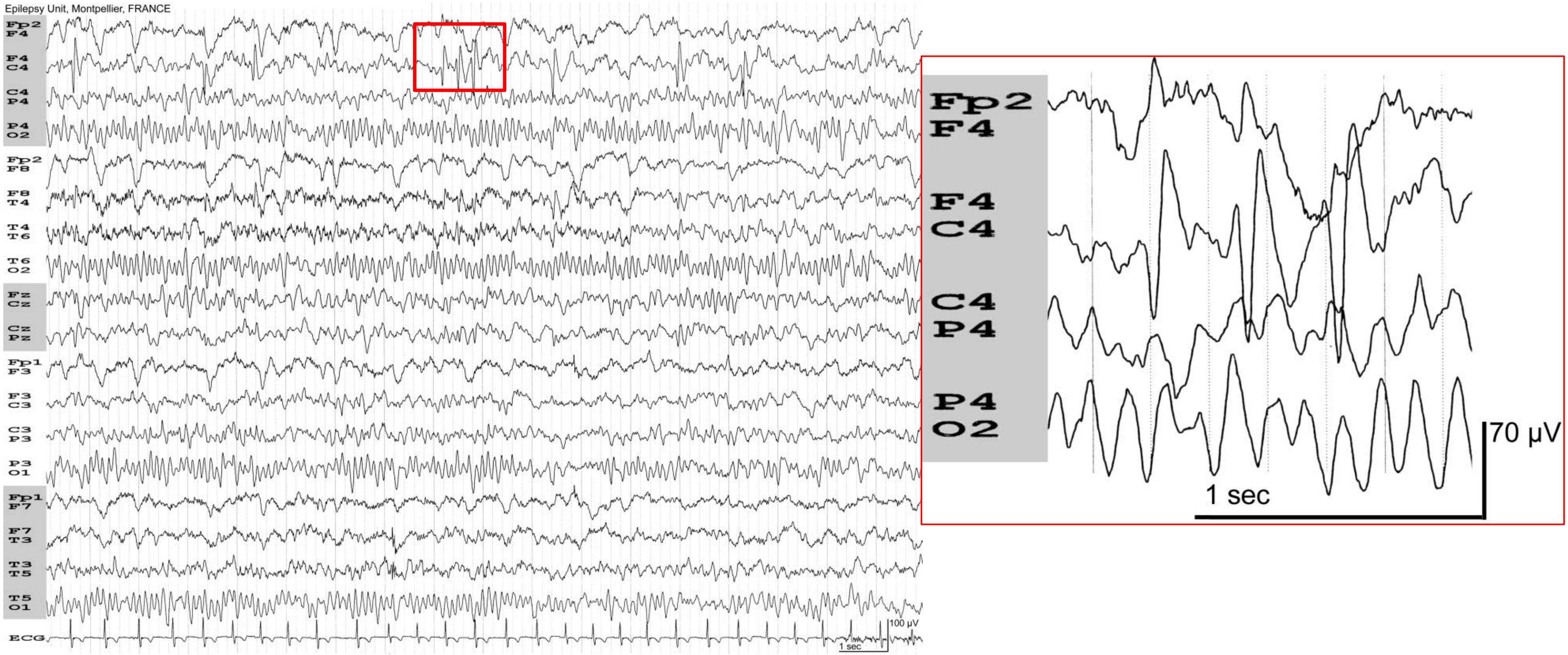

- These spike-waves are characteristic for self-limited epilepsy with centrotemporal spikes formerly known as benign Rolandic epilepsy or benign epilepsy of childhood with centrotemporal spikes but this child had no seizure.
- In this child, the spike-waves observed on the EEG evoke age-dependent functional spikes. They can be observed in children who do not display epilepsy. Beaussart (1972) reported that, in a cohort of 315 children with rolandic spikes, 16% were not epileptic. Cavazzuti et al. (1980) reported rolandic spikes in 2.3% if 3,726 children.
- The changes disappear at puberty.

19-year-old woman with a typical vasovagal syncope

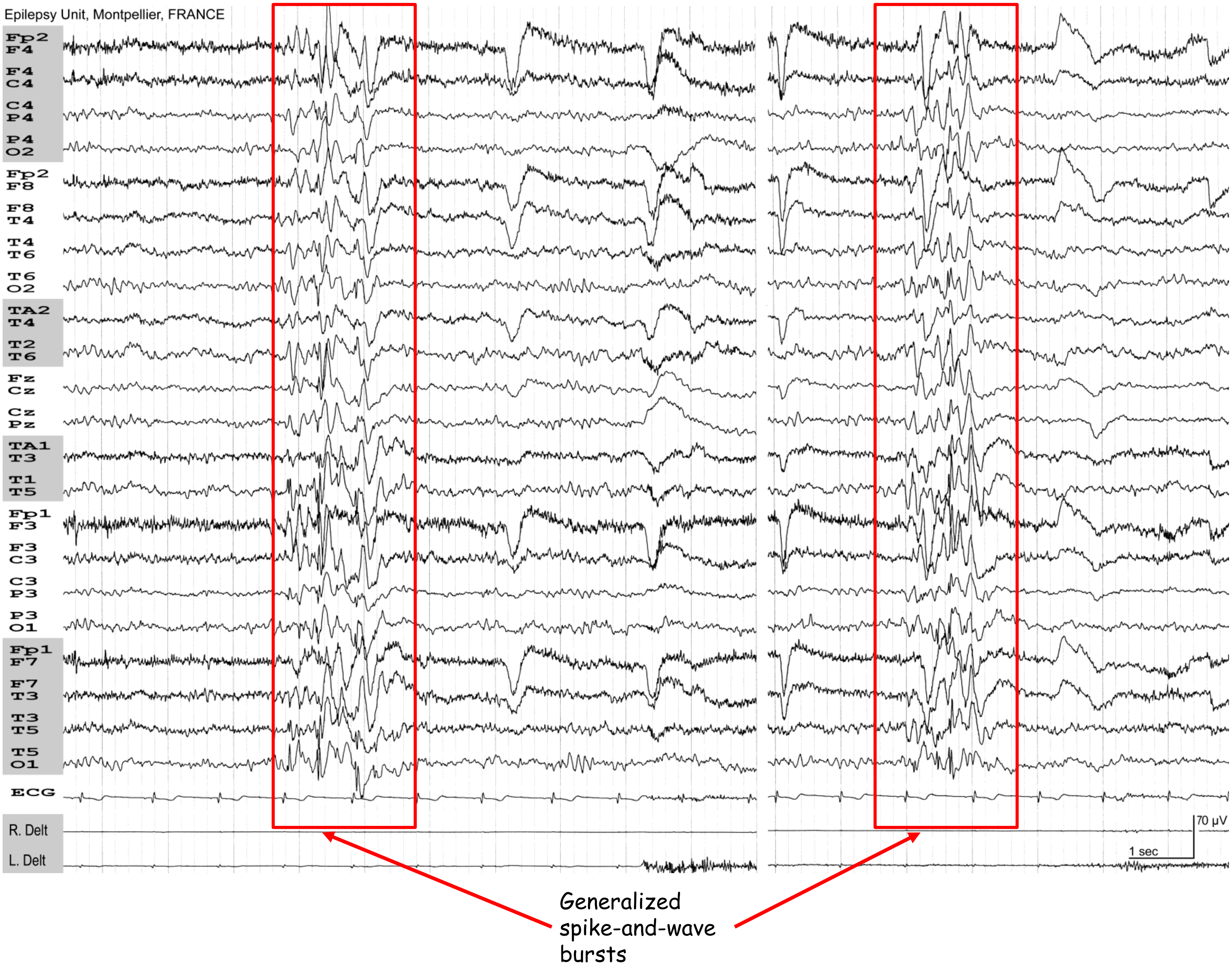

19-year-old woman with a typical vasovagal syncope

- The EEG pattern evokes an genetic (idiopathic) generalized epilepsy but the patient had no seizure (generalized tonic-clonic seizure, myoclonic or absence seizure).
- Her antiseizure medication was stopped after the EEG. But she had a genetic predisposition to genetic generalized epilepsy. She was advised to avoid sleep deprivation and alcohol abuse.
- This example indicates how important the clinical context is when interpreting an EEG. The presence of spike-wave does not necessarily indicate that a patient has epilepsy. There should not have been an EEG.

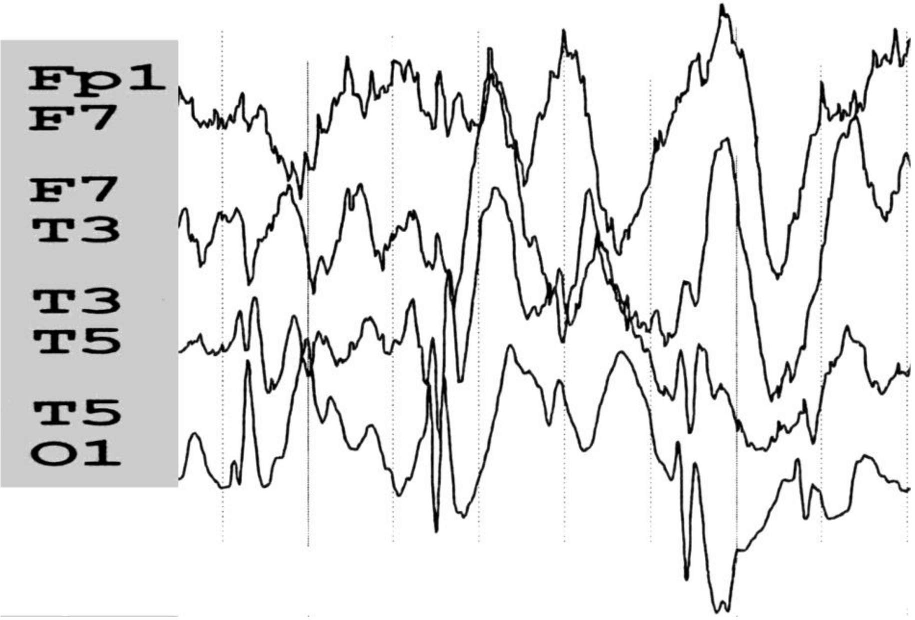

References

- Beaussart M. Benign epilepsy of children with Rolandic (centro-temporal) paroxysmal foci. A clinical entity. Study of 221 cases. Epilepsia 1972;13:795-811. doi: 10.1111/j.1528-1157.1972.tb05164.x

- Cavazzuti GB, Cappella L, Nalin A. Longitudinal study of epileptiform EEG patterns in normal children. Epilepsia. 1980;21:43-55. doi: 10.1111/j.1528-1157.1980.tb04043.x

- Gélisse P, Crespel A. Powerful activation of lambda waves with inversion of polarity by reading on tablet. Epileptic Disord. 2024. doi: 10.1002/epd2.20197. OPEN ACCESS

- Maulsby RL. Some guidelines for assessment of spikes and sharp waves in EEG tracings. Am J EEG Technol 1971; 11:3-16. doi: 10.1080/00029238.1971.11080808

- Naquet R, Louard C, Rhodes J, Vigouroux M. [Apropos of certain paroxysmal discharges from the temporoparieto-occipital junction. Their activation by hypoxia]. Rev Neurol (Paris) 1961;105:203-7.

- Velizarova R, Crespel A, Serafini A, Gelisse P. A new approach for the detection of the fourteen- and six-Hertz positive bursts (6-14 Hz): the lower temporal line. Clin Neurophysiol. 2011 Jun;122(6):1272-3. doi: 10.1016/j.clinph.2010.09.009
